# Polymorphism and surface diversity arising from stress-induced transformations – the case of multicomponent forms of carbamazepine

**DOI:** 10.1107/S2052520620015437

**Published:** 2021-01-16

**Authors:** Gabriela Schneider-Rauber, Mihails Arhangelskis, Andrew D. Bond, Raimundo Ho, Nandkishor Nere, Shailendra Bordawekar, Ahmad Y. Sheikh, William Jones

**Affiliations:** a Department of Chemistry, University of Cambridge, Lensfield Road, Cambridge, CB2 1EW, United Kingdom; b Postgraduate Programme of Pharmacy, Federal University of Santa Catarina, Delfino Conti Street, Florianópolis, 88040-900, Brazil; c Faculty of Chemistry, University of Warsaw, 1 Pasteura Street, Warsaw, 02-093, Poland; d Process Research and Development, AbbVie, Inc., North Chicago, IL 60064, USA

**Keywords:** crystal engineering, stress-induced transformation, carbamazepine, solvate, cocrystal, hydrate

## Abstract

Stress-induced transformations of labile multicomponent organic solids may have a significant impact on industrial manufacturing processes, for example, in the pharmaceutical field. This study considers 15 carbamazepine (CBZ) multicomponent crystal forms, with the aim of identifying the structural and surface features that drive the outcome of thermal stress-induced transformations. Analysis of the crystal structures, and specifically the degree of similarity with the CBZ polymorphs produced by desolvation-like processes, identifies some degree of correlation between structural features. In particular, mutually exclusive supramolecular motifs identified previously within CBZ crystal structures are frequently (but not invariably) preserved, and thereby provide some indication of the anticipated polymorphic outcome. This is broadly consistent with established models relating reactant and product crystal phases. Some of the CBZ multicomponent materials show surface modifications indicative of the formation of a liquid intermediate phase, which provides an alternative dissolution/recrystallization mechanism and different polymorphic outcomes compared to the direct solid–solid transformation pathway. Other cases show intermediates of varying stoichiometry and instances of chemical decomposition. Hence, the product of thermal decomposition is frequently affected by the physical properties of the coformer, such as boiling point and reactivity. This can lead to a dependence on experimental conditions, especially when events such as recrystallization, chemical decomposition of the coformer, solubilization and peritectic melting occur concomitantly. This study highlights that the overall picture is complex, even within this series of closely related materials.

## Abstract 2

Labile multicomponent crystal forms of carbamazepine have been studied with an aim of identifying the structural and surface features that drive the outcome of thermal stress-induced transformations.

## 1. Introduction   

Stress-induced transformations are processes that may involve changes in the internal structure and/or the shape and volume of a crystal as a result of stress and strain being accumulated and released from the crystal lattice. An early observation in organic solids was in the case of 1,8-di­chloro-10-methyl­anthracene under transmission electron microscopy (Jones *et al.*, 1975 ▸). The authors observed crystallites with faulted areas believed to result from the stress associated with rapid cooling of the specimen for study at low temperature (*ca* −173 °C). The diffraction patterns of the resulting domains showed an ordered and coherent lattice rearrangement. The transformation, believed to be martensitic in nature, was reversible following an increase of temperature and a relaxation of the stress. Prior to the 1,8-di­chloro-10-methyl­anthracene study, the generally accepted idea was that structure correlation did not necessarily exist between the parent and product crystal (Mnyukh *et al.*, 1965 ▸; Mnyukh, 1963 ▸; Kitaigorodskii, 1973 ▸). Whether the structural relationship is a result of oriented nucleation or a direct crystallographic relationship, the current most accepted approach for the analysis of solid transformations in organic materials, including desolvation/dehydration, takes into account the structure of the parent and daughter phases and the analysis of various mechanistic aspects (Byrn *et al.*, 1999 ▸; Petit & Coquerel, 1996 ▸; Galwey, 2000 ▸; Petit & Coquerel, 2009 ▸).

Multicomponent crystal forms of pharmaceuticals, especially hydrates, are frequently observed (Stahly, 2007 ▸; Cruz-Cabeza *et al.*, 2015 ▸) and are increasingly common in recently approved drugs (Caspi *et al.*, 2019 ▸; Cink *et al.*, 2020 ▸; Califano *et al.*, 2016 ▸; Brackemeyer *et al.*, 2017 ▸; Pangan *et al.*, 2018 ▸). Hydrates, solvates and cocrystals present challenges during development because of the implications for *in vivo* solubility, manufacturing and storage (Hilfiker *et al.*, 2006 ▸; Pudipeddi & Serajuddin, 2005 ▸; Threlfall, 1995 ▸). For instance, the solubility of a hydrated form can be significantly lower in physiologically relevant media, and the control of water of hydration during manufacturing and storage can lead to complex processing and modified packaging configurations. Furthermore, for relatively flexible molecules which cannot pack efficiently, hydrates and solvates can be the only form that can be crystallized or manufactured. In such cases, dehydration or desolvation is then the only practical option for obtaining (if required) solvent-free solid forms (Griesser, 2006 ▸; Catron *et al.*, 2016 ▸).

**Figure 1:**
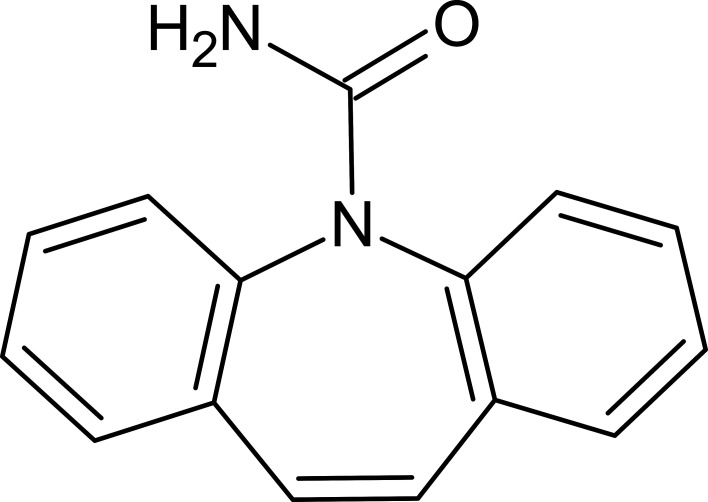
The molecular structure of carbamazepine (CBZ).

Such aspects were the motivation behind the present work. We report here on stress-induced transformations for some multicomponent carbamazepine (CBZ) (Fig. 1 ▸) materials, using temperature as the external driving force. Several characteristics of the solids are evaluated, including the nature of the guest molecules, the hydrogen-bonding strength of the guest and host molecules, crystal packing and the arrangement of the coformer on the outcome of guest loss. Importantly, since the CBZ molecule is rigid and does not show intramolecular hydrogen bonding, stresses deriving from torsions within the host molecule are expected to be less during any transformations. The ultimate aim is to establish structure-to-property relationships for stress-induced transformations which can inform pharmaceutical manufacturing, especially in the case of labile multicomponent organic solids.

## 2. Crystal structure analysis applied to stress-induced transformations   

### 2.1. Background   

Petit & Coquerel (1996 ▸) have proposed a unified model (Rouen 96) for the dehydration of molecular crystals which may also be expanded to solvates and other desolvation-like processes. The model is based on the existence of planes or channels in the structure and whether these are crystallographic features present in the parent phase or which develop during the early stages of reaction. In cases where the structural filiation requirement is met and the crystal lattice energy from the host molecules is greater than the energy contribution from the interactions with the solvent, the processes are classified as co-operative. A co-operative release mechanism can then lead to either a desolvated material with no (or little) structural reorganization or to the co-operative rearrangement of the molecules characterized by a structural filiation relationship. The latter only occurs if the domains formed upon desolvation are above a critical size.

Byrn *et al.* (1999 ▸) also suggested that the outcome of desolvation of a channel-like structure is mainly influenced by the packing around the channels. The authors highlight other characteristics which may affect the reaction in a system-specific way: the channel size, the number of channels per unit area, the density of host molecules, the number and strength of hydrogen bonds to the solvent molecule, the coordination in the case of salts, the direction of the solvent chain relative to weak planes in the structure, the position of the channel related to the host molecule and the tortuosity (degree of twisting) of the channel. These characteristics seem to be correlated to the overall lattice energy and the energy of interaction with solvent molecules considered in the ‘Rouen 96’ model.

Galwey (2000 ▸) also contributed to the discussion on the mechanistic aspects of desolvation reactions by taking diffusion phenomena into consideration. The author highlighted that the gradient of concentration along a structural channel or plane is the driving force towards desolvation. The energy released during the diffusion of one molecule can also be transferred directly to its neighbour in a chain reaction. This chain-type reaction may be catalysed by charge effects and the presence of strain and defects, which may not be directly accounted for in the balance between lattice and solvent interaction energy.

In summary, the literature features two main approaches for comparing the structures of crystals subjected to desolvation-like phase transformations: an analysis of the crystal packing and an understanding of the intermolecular interactions between the molecules in the crystals. While the analysis of packing focuses on a comparison of the parent and daughter phases, and on the arrangement of the guest molecules, the analysis of the intermolecular interactions assesses the strength of contact between host and guest.

### 2.2. Carbamazepine (CBZ) crystal forms   

CBZ has been studied extensively in the field of pharmaceutical materials science. In the present study, we sought to consider a broad set of CBZ multicomponent materials containing coformers amenable to desolvation-like transformations. Salts of CBZ were deliberately excluded. On this basis, 13 CBZ forms were selected, as indicated in Table 1 ▸. New structures with different stoichiometries were obtained during crystallization with benzo­quinone (BZQ) and tri­fluoro­ethanol (TFE), producing a final set of 15 multi-component forms (Table 1 ▸). Amongst the structures, there are several isostructural sets, as indicated in Table 1 ▸ and Tables S2 and S3 in the supporting information.

**Figure 2:**
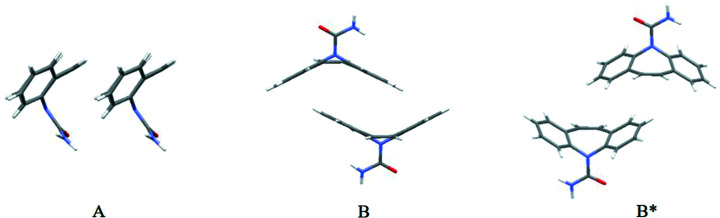
Mutually exclusive motifs **A** and **B** identified by Gelbrich & Hursthouse (2006 ▸). Motif **A** is found in polymorphs I and II. Motif **B** is found in polymorphs III and IV. Motif B* is an offset version of **B**, occurring in CBZ·DMA and CBZ·BZQ.

Crystal structures containing CBZ have been considered in several previous studies (Childs *et al.*, 2009 ▸; Gelbrich & Hursthouse, 2006 ▸; Fleischman *et al.*, 2003 ▸; Cruz-Cabeza *et al.*, 2006 ▸, 2007 ▸). The detailed analysis of Gelbrich & Hursthouse (2006 ▸; abbreviated to G&H) identified a number of core motifs (supramolecular constructs) within CBZ crystal forms, and developed relationships between them. One of the most recognisable motifs is the *R* 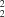
(8) hydrogen-bonded dimer formed between CBZ molecules (denoted **C** in G&H). This is found in most of the polymorphs and multicomponent structures herein, except for CBZ Form V, CBZ·FA, CBZ·ACA and CBZ·TFA. Hence, desolvation of the latter three structures to produce one of the CBZ polymorphs containing the *R* 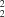
(8) motif between CBZ molecules requires significant rearrangement of the hydrogen bonding between CBZ molecules, which is not required for the other structures. G&H identified two mutually exclusive motifs (denoted **A** and **B**), which occur in all of the structures. Motif **A** comprises CBZ molecules stacked along a short (*ca* 5 Å) axis, while motif **B** is a ‘back-to-back’ arrangement between the dibenzoazepine portions of CBZ (Fig. 2 ▸). CBZ·DMA and CBZ·BZQ show a distorted version of motif **B** (denoted B* in Table 1 ▸) which possibly should be considered to be distinct; however, the motif clearly resembles **B** rather than **A**. Notably, CBZ polymorphs I and II include motif **A**, while polymorphs III and IV include motif **B**. Given the mutually exclusive nature of **A** and **B**, we might expect transformations preserving motif **A** or **B** to be most facile.

**Figure 3:**
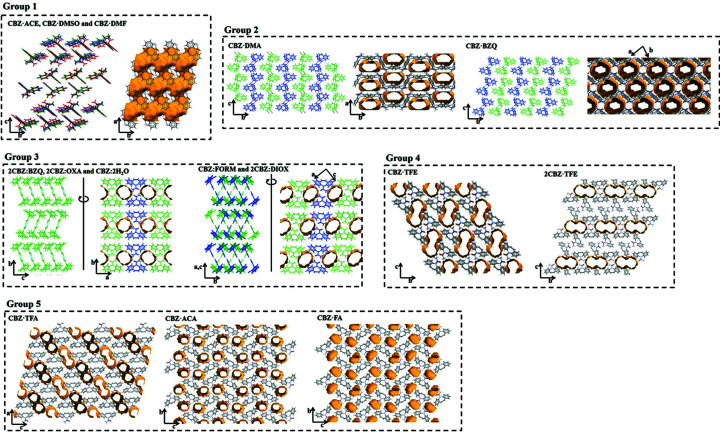
Structural comparison of the studied carbamazepine multicomponent materials. Guest molecules removed for clarity.

Of specific interest here is the nature of the void space occupied by the coformer molecules. On this basis, we classify the structures into five groups (Table 1 ▸ and Fig. 3 ▸). Group 1 comprises the isostructural ACE and DMSO solvates, and the closely related DMF solvate, all of which contain two-dimensional (2D) intersecting channels parallel to the crystallographic *ab* planes (Fig. 3 ▸). The structures show 2D similarity with polymorph III, where layers of molecules in the *ab* planes in III correspond to those in the *ac* planes of the multicomponent forms. Group 2 comprises the DMA and 1:1 BZQ structures, which display 2D similarity with each other (in their *ac* planes) and contain one-dimensional (1D) channels formed principally between the dibenzoazepine portions of CBZ. The planar DMA/BZQ molecules accept hydrogen bonds from the carboxamide groups and are sandwiched between the aromatic rings of neighbouring CBZ molecules. Group 3 comprises five compounds, forming two distinct structure types with 1D channels between the carboxamide groups of CBZ. The channels run parallel to the short axis defined by motif **A**. The two structure types within the group, {2CBZ·OXA, CBZ·2H_2_O, 2CBZ·BZQ} and {CBZ·FORM, 2CBZ·DIOX} (OXA is oxalic acid, FORM is formamide and DIOX is 1,4-dioxane), are polytypes, containing identical layers stacked either by translation or in a herringbone manner (Fig. 3 ▸). Group 4 comprises two different structures containing TFE, both of which contain the solvent molecules in 1D channels parallel to the translational motif **A**, as in Group 3. Locally, the positions of the TFE molecules with respect to the CBZ molecules are identical in the two structures, but the relative arrangements of the *R* 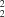
(8) CBZ dimers is different. CBZ·TFE resembles more closely Group 3, while 2CBZ·TFE displays a herringbone-type arrangement in projection along the channels (Fig. 3 ▸). Finally, Group 5 comprises CBZ·FA, CBZ·ACA and CBZ·TFA, where the solvent molecules disrupt the CBZ *R* 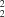
(8) hydrogen-bonded dimers.

### 2.3. Interaction energies and IR spectroscopy   

The nature of the interactions between CBZ and coformer molecules was assessed through a combination of intermolecular energy calculations (Table 2 ▸) and IR spectroscopy (Figs. S2–S5 in the supporting information). The calculations were applied using the UNI force-field potential (Gavezzotti, 1994 ▸; Gavezzotti & Filippini, 1994 ▸) within *Mercury* (Macrae *et al.*, 2020 ▸), which gives an indicative picture of the relative interaction strength. In all cases, the coformer molecules accept N—H⋯O hydrogen bonds from the CBZ carboxamide groups, side-on to the *R* 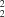
(8) CBZ dimers, or CBZ–coformer pairs in Group 5. For the coformers with hydrogen-bond donors, O—H⋯O hydro­gen bonds are also formed to the CBZ molecules. As would be expected, stronger interactions are found between CBZ and the coformer molecules linked *via* O—H⋯O hydrogen bonds (CBZ·TFE, 2CBZ·TFE, CBZ·TFA, CBZ·ACA and CBZ·FA). In the case of Group 5, the interactions are even stronger owing to the formation of multipoint *R* 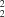
(8) motifs between CBZ and the coformer molecules. This is manifested by markedly different IR spectra, in comparison to the CBZ polymorphs and the other multicomponent forms. Starting from CBZ·FA, to CBZ·ACA and then to CBZ·TFA, the CBZ carbonyl stretching band shifts to higher frequencies and the N—H bending bands change substantially and are also shifted to higher energy. It has been suggested in the literature that the interaction between CBZ and TFA holds an intermediate ionic character (Eberlin *et al.*, 2013 ▸), which is consistent with the observed high-frequency C=O stretch for CBZ·TFA. It is possible that CBZ·FA and CBZ·ACA [p*K*_*a*_ ≈ 3.8 (FA) or 4.8 (ACA)] also display some degree of ionic character, although not as significant as TFA (p*K*_a_ ≈ 0.2). Strong interactions between CBZ and the coformer are also observed in Group 2 and for CBZ·DMF in Group 1 as a result of the planar coformer lying across the dibenzoazepine portions of the CBZ molecules. The intermolecular energies in Table 2 ▸ quantify the *total* interaction between the coformer molecule and CBZ, which must also include a significant dispersion contribution in these cases.

CBZ·2H_2_O, CBZ·TFE and 2CBZ·TFE show significantly different magnitudes of the CBZ–coformer interactions in the same structure, reflecting distinct environments for the coformer molecules. It is also notable that the BZQ and TFE systems present structures with alternative CBZ:coformer stoichiometries. In CBZ·TFE and 2CBZ·TFE, the local interactions between TFE and CBZ molecules are similar, but 2CBZ·TFE contains additional CBZ molecules that are not involved in direct hydrogen bonding to TFE. Similarly, in CBZ·BZQ, one carbonyl group of each BZQ molecule accepts an N—H⋯O hydrogen bond from CBZ, while the other does not. In 2CBZ·BZQ, both carbonyl groups accept N—H⋯O hydrogen bonds. These differences are clearly seen for the carbonyl stretching band in the IR spectra. The contribution of weak or ‘unsatisfied’ interactions during desolvation may be significant.

The interactions between coformer molecules are, in general, much weaker than those between coformer and CBZ, except where the coformer molecules form hydrogen bonds to each other, *i.e.* CBZ·2H_2_O, CBZ·FORM, CBZ·TFE and 2CBZ·TFE.

## 3. Thermal decomposition studies of carbamazepine multicomponent forms   

**Figure 4:**
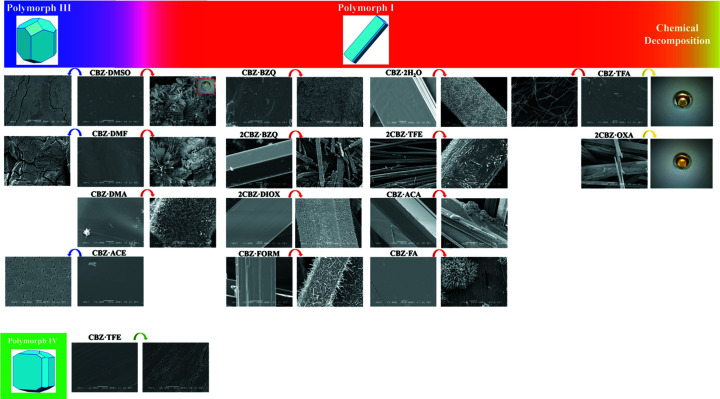
Summary of the outcomes of the thermal stress-induced transformations of CBZ multicomponent materials. Detailed data is available in §S3 of the supporting information.

The study of desolvation-like processes upon thermal treatment (McCrone, 1957 ▸) was selected as an example of stress-induced transformations representative of events which may take place in the manufacturing of pharmaceuticals, *e.g.* drying, granulation, *etc*. Fig. 4 ▸ and Table 3 ▸ summarize the results of the experiments in this context. The objective is to describe the solid-state properties at play with regard to the surface and structural reorganizations that result from the stress within crystals of labile multicomponent organic solids.

### 3.1. Group 1: CBZ acetone (CBZ·ACE), CBZ di­methyl sulfoxide (CBZ·DMSO) and CBZ *N*,*N*-di­methyl­formamide (CBZ·DMF) solvates   

Single crystals of CBZ·ACE turn opaque on heating, while the original shape of the particles is maintained (see §S3.1 in the supporting information). The *in situ* powder X-ray diffraction (PXRD) experiments agree with the thermal data and demonstrate that desolvation results in the formation of polymorph III, while detectable reflections of Form I are observed above 160 °C. The *ex situ* scanning electron microscopy (SEM) analyses of CBZ·ACE crystals subjected to non-isothermal desolvation at a rate of 10 °C min^−1^ indicate the formation of round domains and holes between a grain-like structure. Cracks are rarely seen, but when detected, the fractures are irregular and do not appear to be correlated to particular crystallographic planes.

In contrast to CBZ·ACE, single crystals of CBZ·DMF and CBZ·DMSO do not lose birefringence uniformly as desolvation proceeds (see §S3.2 and §S3.3). The crystals develop opacity and round domains, which grow throughout the crystal as the temperature is increased. SEM analyses agree and show a substantial surface reorganization with the simultaneous formation of spherulites, which are composed of grouped needles, with round surface domains and more isotropic particles grown from the original crystals. These results are considerably different from the outcome of CBZ·ACE desolvation at the same heating rate. Non-isothermal *in situ* PXRD and differential scanning calorimetry/thermogravimetric anal­ysis (DSC/TGA) indicates that the desolvation at a rate of 10 °C min^−1^ is likely to be complex, with the formation of a peritectic mixture in which the solid CBZ·DMF/CBZ·DMSO and anhydrous CBZ mix with liquid DMF/DMSO. The rapid decrease of peak intensities in the diffractograms, the varying slopes throughout the desolvation event seen in the thermal curves and the imaging of desolvated materials are indicative of such a phenomenon.

It is suggested that DMSO and DMF are released from the crystal and remain as a liquid layer around the crystal due to their high boiling points. When a peritectic is formed it is possible that pure desolvation of CBZ·DMF and CBZ·DMSO results in Form III, while CBZ dissolved in the liquid at high temperature accounts for the recrystallization of Form I. This hypothesis is supported by the fact that the rate of desolvation is found to affect the outcome of the transformation. Experiments performed under milder conditions may provide an environment in which the solvent is more likely to be released into the vapour phase and no peritectic is formed. Indeed, the desolvation of CBZ·DMF and CBZ·DMSO at a rate of 1 °C min^−1^ results in Form III particles with surfaces containing round domains and holes, similar to the desolvated CBZ·ACE surfaces at a rate of 10 °C min^−1^. In the case of CBZ·DMF and CBZ·DMSO, however, cracks are seen more frequently. While the fractures do not appear to be correlated to crystallographic planes in the solvate lattice, they appear to propagate along the edges of the surface of freshly formed domains and through holes.

### 3.2. Group 2: CBZ *N*,*N*-di­methyl­acetamide solvate (CBZ·DMA) and 1:1 CBZ benzo­quinone cocrystal (CBZ·BZQ)   

As in the cases of CBZ·DMSO and CBZ·DMF, single crystals of CBZ·DMA and CBZ·BZQ do not lose birefringence uniformly but develop opaque and round domains which grow throughout the crystal as the temperature increases (see §S3.4 and §S3.5). *Ex situ* SEM analysis of CBZ·DMA illustrates the reorganization of the surface after non-isothermal desolvation at a rate of 10 °C min^−1^ and shows the development of whiskers and spherulites to the point that crystals lose their original shape. In the case of CBZ·BZQ, the product apparently nucleates from defective regions on the surface and the formation and growth of these domains leads to a substantial surface reorganization but does not cause loss of the crystal shape as seen for CBZ·DMA.

*In situ* PXRD shows that desolvation of CBZ·DMA results in Form III, although the outcome of desolvation suggested by surface imaging does not show, curiously, the morphology usually associated with this polymorph. The reasons why PXRD and SEM analyses are not consistent are unclear. It is possible that the results of desolvation of the samples are distinct. In this case, differences in sample size may change the local environment during desolvation and kinetically affect the process.

*In situ* PXRD and thermal analyses of CBZ·BZQ show gradual changes with temperature increase. The experiments demonstrate that removal of BZQ first leads to the formation of the lower stoichiometric 2CBZ·BZQ cocrystal. Further removal of BZQ then results in the conversion of 2CBZ·BZQ into CBZ Form I, indicating that although CBZ·DMA and CBZ·BZQ are structurally similar, they result in different CBZ polymorphs upon heating. It is suggested that the existence of a stable cocrystal form with an intermediate stoichiometry in the case of CBZ/BZQ influences the mechanism by which the structure rearranges. In this case, a slower and progressive transformation is observed for the sublimation of CBZ·BZQ. Also, the similarities between 2CBZ·BZQ and Form I may drive the outcome of sublimation of the resulting BZQ cocrystals.

### 3.3. Group 3: 2:1 CBZ 1,4-benzo­quinone (2CBZ·BZQ) and oxalic acid (2CBZ·OXA) cocrystals, CBZ dihydrate (CBZ·2H_2_O), and CBZ formamide (CBZ·FORM) and 1,4-dioxane (2CBZ·DIOX) solvates   

As expected, the analysis of 2CBZ·BZQ (see §S3.5) demonstrates that decomposition starts at a higher temperature than for CBZ·BZQ. Although the thermal curves and the polymorphic outcome of decomposition of both BZQ cocrystals are the same, they have significantly different morphology and surface characteristics. Single crystals of 2CBZ·BZQ show a needle-like morphology and turn uniformly opaque on heating and maintain the original shape of the particles, while whiskers progressively form on the surface. The *ex situ* SEM analyses of the crystals resulting from 2CBZ·BZQ after sublimation show that the domains of the newly formed product are oriented along the needle axis. This anisotropic surface rearrangement differs quite significantly from the behaviour of CBZ·BZQ subjected to heating experiments. It was nevertheless seen consistently in other Group 3 multicomponent structures.

Regardless of their composition, the product resulting from the decomposition of 2CBZ·BZQ, CBZ·2H_2_O (§S3.7), CBZ·FORM (§S3.8) and 2CBZ·DIOX (§S3.9) show similar characteristics. The crystals uniformly turn opaque as a result of heating and the desolvated samples show the formation of oriented needle-like structures, which are characteristic of CBZ Form I. In fact, *in situ* PXRD analysis shows that polymorph I is the outcome of all CBZ multicomponent forms classified in Group 3, except 2CBZ·OXA. Differences in the morphology of CBZ·2H_2_O samples do not affect the polymorphic outcome of dehydration. Only the kinetics of the process have changed, possibly because of variations of the particle size of the dihydrate samples. In addition, the results demonstrate that the dihydrate and the formamide solvate undergo isostructural dehydration/desolvation and lattice modification only after the release of a significant amount of solvent. 2CBZ·BZQ and 2CBZ·DIOX crystals, in turn, show recrystallization and coformer evolution in a concomitant step and do not appear to form an isostructural desolvate as for CBZ·2H_2_O and CBZ·FORM.

Optical micrographs taken during hot stage analysis of 2CBZ·OXA show that the crystals become opaque and whiskers grow on the crystals above 130 °C (see §S3.6). Interestingly, the images demonstrate that the resulting product concomitantly liquefies and sublimes above 160 °C. *In situ* PXRD heating experiments did not detect the formation of CBZ polymorphs upon heating, although the diffractograms show evidence of lattice collapse above 160 °C (*i.e.* no remaining diffraction peaks), perhaps because of the formation of a liquid phase. The thermograms of 2CBZ·OXA provide further insight about its decomposition. The shape and slopes of the TGA curve above 150 °C are similar to the characteristics of the thermal decomposition of CBZ alone. In the case of 2CBZ·OXA, however, these events are shifted to lower temperatures. The results may demonstrate that the physical decomposition of the cocrystal accelerates the chemical decomposition of CBZ without a change in mechanism. Visual observations of the remaining molten residues evidence that not only pure sublimation is taking place, but also a chemical reaction.

The comparison of the characteristics of benzo­quinone (which sublimes without decomposition) and oxalic acid (which decomposes with product sublimation) may explain the difference in the thermal behaviour of 2CBZ·BZQ and 2CBZ·OXA. It demonstrates that, although the crystal forms in Group 3 are structurally related, their thermal behaviour is greatly influenced by the properties of the guest molecule.

### 3.4. Group 4: CBZ 2,2,2-tri­fluoro­ethanol (CBZ·TFE and 2CBZ·TFE) solvates   

Single crystals of CBZ·TFE show evidence of peritectic formation at the initial stages of desolvation, which takes place below the boiling point of tri­fluoro­ethanol (see §S3.10). As desolvation proceeds, the crystals become opaque under polarized light, while the original shape of the crystals is maintained. The surface of the product of desolvation appears as elongated domains, with holes between neighbouring grain boundaries. The alignment and the contact between domains frequently result in the formation of cracks that do not appear to follow any specific crystallographic orientation. *In situ* PXRD experiments show a sudden lattice change above 80 °C, resulting in the formation of CBZ polymorph IV. In contrast to the behaviour of the benzo­quinone cocrystals, the experiments demonstrate that the solvate with lower stoichiometry is not an intermediate of the decomposition of CBZ·TFE.

Single crystals of 2CBZ·TFE heterogeneously lose birefringence upon heating, while the original shape of the particles is maintained (see §S3.10). The surface domains of the newly formed product are oriented along the needle axis. The results show that the 1:1 and 2:1 tri­fluoro­ethanol solvates do not only show dissimilar surface characteristics, but also exhibit different thermal curves and polymorphic outcome upon desolvation. *In situ* PXRD analysis shows the decomposition of 2CBZ·TFE starts at higher temperature than observed for CBZ·TFE and the solvate rapidly rearranges into CBZ polymorph I upon heating above 100 °C.

### 3.5. Group 5: CBZ tri­fluoro­acetic acid (CBZ·TFA), CBZ acetic acid (CBZ·ACA) and CBZ formic acid (CBZ·FA) solvates   

The crystals of CBZ·TFA turn opaque on heating and visual observation shows that the reaction product was frequently molten (see §S3.11). Indeed, *in situ* PXRD heating experiments performed at a rate of 10 °C min^−1^, show evidence of lattice collapse and the formation of a liquid phase above 130 °C. Similar to the observations for 2CBZ·OXA, the CBZ·TFA thermograms demonstrate that physical decomposition of the cocrystal accelerates the chemical decomposition of CBZ as the events are shifted to lower temperatures. A difference in the behaviour of 2CBZ·OXA and CBZ·TFA is seen in the effect of heating rate on their thermal characteristics. The thermograms of 2CBZ·OXA do not differ as a function of heating rate, but the CBZ·TFA thermograms do. Unlike the experiments performed at 10 °C min^−1^, the de­sol­vation event at 1 ^o^C min^−1^ is clearly separated from the other weight-loss events corresponding to the decomposition of CBZ. In general, the results show that the rate of desolvation of CBZ·TFA affects the rate and mechanism of chemical decomposition of CBZ.

Crystals of CBZ·ACA (§S3.12) and CBZ·FA (§S3.13) turn opaque upon heating and the overall shape of the particles is lost. Similar to CBZ·2H_2_O and CBZ·FORM, the results demonstrate that the acetic acid solvate undergoes isostructural desolvation and shows a rapid lattice modification into Form I only after the release of a significant amount of solvent. CBZ·FA, in turn, desolvates to give CBZ polymorph I, following a gradual reaction. In both cases, additional analyses suggest heating leads to concomitant events, such as de­solv­ation, recrystallization and melting, or the formation of a peritectic mixture. In fact, evidence of melting was sometimes observed and characterized as spherulite growth on the crystal surface, in these materials and in others. In general, however, *ex situ* SEM analyses of the desolvated samples show the formation of oriented needle-like domains on the surface of the crystals.

## 4. Crystal-to-property relationships   

### 4.1. The effect of crystal packing on the outcome of stress-induced transformations   

The analysis in §2 highlights the similarities and differences between the CBZ polymorphs and in the multicomponent materials. In broad terms, the structural similarities are translated into similar thermal properties. For example, the structures classified in Group 1 transform directly to polymorph III, in accordance with 2D structural similarity, retaining motif **B** (Fig. 2 ▸). Similarly, CBZ·DMA (Group 2) contains the offset motif B* and transforms directly to polymorph III. By contrast, the structures classified in Groups 3, 4 and 5 contain motif **A** and transform predominantly to polymorph I, preserving motif **A**. The two principal exceptions to these general rules are the BZQ and TFE materials, both of which display variants with different stoichiometry. For CBZ·BZQ (Group 2), the initial transformation to 2CBZ·BZQ does not follow any obvious structural similarity beyond the CBZ *R* 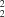
(8) hydrogen-bonded dimers present in all of the polymorphs, although it may be significant that the transformation enables both carbonyl groups of BZQ to accept N—H⋯O hydrogen bonds in 2CBZ·BZQ. Once 2CBZ·BZQ is produced, the transformation to CBZ polymorph I is consistent with that seen for 2CBZ·BZQ itself (Group 3). In Group 4, 2CBZ·TFE follows the anticipated transformation to polymorph I, but CBZ·TFE is unique in transforming directly to polymorph IV, with no evidence of the intermediate 2CBZ·TFE. The CBZ·TFE→polymorph IV transformation is the only example in the studied set where a structure containing motif **A** transforms to a structure containing motif **B**.

The appearance and orientation of initial surface domains vary quite significantly depending on the polymorphic form that is obtained after transformation. The surfaces of the resulting crystals characterized as CBZ polymorph III or IV were smoother and, in general, showed holes and round grains which are characteristic of the morphology of Forms III and IV. By contrast, the domains on the surface of those crystals correlated to the formation of CBZ polymorph I were characterized as acicular or whisker-like. Frequently, the surface domains were oriented along the dominant particle axis itself, suggesting some correlation with the underlying arrangement of CBZ molecules. In some cases, it was also observed that surfaces of the same crystal had domains oriented in different directions. This orientation effect illustrates that transformations are strongly influenced by structural anisotropy and could be correlated to the direction of molecular transport, lattice rearrangement and/or crystallite growth.

The effect of structure on the course of the transformations appears to be more important than the thermodynamic relationship between the various CBZ polymorphs. CBZ Form I is the most stable form at high temperature, while Form III is the most stable form under ambient conditions (Behme & Brooke, 1991 ▸; Umeda *et al.*, 1984 ▸). The polymorphs are known to be enantiotropically related and our own studies suggest that the transition occurs above 120 °C (see §S3.14 and Fig. S60 in the supporting information) – although earlier studies have reported different transition temperatures. On this basis, no clear correlation was observed between the Form III→Form I transition temperature and the onset temperature of guest evolution. In turn, CBZ Form I was formed because of crystal decomposition below the enantiotropic transition temperature. Such a transformation pathway has been reported previously (Krahn & Mielck, 1987 ▸, 1989 ▸; McMahon *et al.*, 1996 ▸; Han & Suryanarayanan, 1998 ▸; Otsuka *et al.*, 1999 ▸; Kachrimanis & Griesser, 2012 ▸; Khoo *et al.*, 2013 ▸; Scaramuzza *et al.*, 2018 ▸). It may be explained by the effect of lattice/packing templating, but also by the Ostwald Rule of Stages (Ostwald, 1897 ▸). It is expected that such a reaction tends to transform into a metastable crystal form *via* the smallest loss of free energy. Polymorph I is considered, however, to be a transient state and its formation and detection may be strongly affected by kinetic factors.

With regards to kinetics, it has been shown in the literature that the amorphization of CBZ upon dehydration of CBZ·2H_2_O follows different kinetic models below and above the glass transition temperature (*T*_g_ ≈ 53 °C), and the dehydration outcome is affected by relative humidity and exposure to solvent vapour (Li *et al.*, 2000 ▸; Khoo *et al.*, 2013 ▸; Kachrimanis & Griesser, 2012 ▸). Although complete amorphization was not detected in our studies by *in situ* PXRD non-isothermal heating experiments, the products show a tendency of decreased crystallinity that appears to be structure specific. For instance, CBZ I formed upon dehydration shows background halo and broader peaks in comparison to CBZ I formed from 2CBZ·BZQ, CBZ·FORM and 2CBZ·DIOX, which are structurally related to the dihydrate. Form III product crystals show a less pronounced peak broadening, but variations with structural filiation are also seen. These results indicate a difference in crystallinity and crystallite size among the materials produced by stress-induced transformations. More importantly, such microstructure modifications suggest that the path to the formation of CBZ anhydrous polymorphs forms may be varied among the different multicomponent crystal forms. Indeed, the analyses suggest that the coformer properties play an important role in these differences – as discussed in the next section.

### 4.2. The effect of molten or liquid phase intermediates on the outcome of stress-induced transformations   

Some of the CBZ multicomponent materials subjected to heat show surface modifications which are indicative of the formation of a liquid intermediate phase. This is to say that the solvent released from the lattice remains in contact with the solid material and results in its dissolution (and subsequent recrystallization). This mechanism is clearly different from the alternative co-operative solid–solid transformation pathway and appears to be facilitated by a combination of rate of desolvation and the stress resulting from solvent release. This was clear in the desolvation of CBZ·DMSO and CBZ·DMF (Group 1) for which varying the experimental conditions resulted in different polymorphic outcomes. It is shown that higher desolvation rates result in the formation of a liquid phase, thereby affecting the outcome. Specifically, the high boiling points of DMSO and DMF in comparison to the onset temperature of desolvation allow for the formation of a peritectic mixture.

It is possible, however, that the difference between the onset temperature of desolvation and the boiling points of the respective solvent may not be the only factor that plays a role. For instance, the arrangement of solvent molecules within the crystal lattice appears to be significant. Evidence for the formation of a liquid phase (without chemical degradation of the guest) was also seen in CBZ·DMA; CBZ·FORM; CBZ·TFE; and CBZ·TFA, CBZ·ACA and CBZ·FA. In the case of CBZ·DMA, CBZ·TFE and CBZ·TFA, the volume of the cavities is relatively large and the solvent forms approximate layers within the crystal, while CBZ·FA has guest molecules isolated in pockets. In both cases, desolvation is potentially more destructive than in typical channel-like structures. It is noteworthy that CBZ·DMSO and CBZ·DMF also show a different type of solvent arrangement (*i.e.* intersecting channels), which may be coupled to other factors and give rise to the observed thermal behaviour.

Another aspect that might contribute stress to the lattice is the effect of the strength of intermolecular interactions between host and guest molecules. It was shown in §2.2 that in their respective solvates, DMF, DMSO, DMA, H_2_O, OXA, TFE, TFA, ACA and FA strongly interact with the CBZ molecules. In particular, the materials belonging to Group 5 show heterodimers formed between CBZ molecules and the carb­oxy­lic acid group. It is also worth noting that the hydrogen bonds responsible for the formation of the heterodimer possibly involve the ionization of the species and therefore the local stress caused by solvent release may be particularly high.

Such transformations are likely to be a result of a combination of factors that are not, conceptually or experimentally, easily separated. As a common feature though, the reactions that present an intermediate liquid phase at high temperature tend to result in CBZ polymorph I. This finding is in agreement with the experiments performed in this study and with the previous literature (Ceolin *et al.*, 1997 ▸; O’Mahony *et al.*, 2014 ▸; Grzesiak *et al.*, 2003 ▸), which conclude that Form I is obtained from the molten state and is the thermodynamically most stable form at high temperature. Sheikh *et al.* (2019 ▸) have also studied the relationship between solid–liquid phases on the desolvation of parecoxib sodium ethano­late. The authors showed that under experimental conditions which lead to slow desolvation, the partially desolvated crystal kinetically influences the product. For cases of rapid temperature increase, in turn, a crystal is more likely to reach a peritectic point while still fully solvated. In this case, the outcome follows thermodynamics. As obvious as it may seem, this means that the lattice templating effect (*i.e.* structural filiation) does not play a significant role in transformations which are mediated by a highly defective or liquid intermediate.

### 4.3. Lower stoichiometry crystal form intermediates   

The effect of lattice collapse on the outcome of stress-induced transformations may also be illustrated by materials that present a lower stoichiometry. In the case of the TFE solvates, the difference between the desolvation onset in CBZ·TFE and the boiling point of TFE is not large. This characteristic and the general thermal behaviour of these crystal forms are consistent with interesting structural features which may also indicate that solvent release generates significant stress in the lattice. The solvent molecules in CBZ·TFE are strongly bound to one of the two CBZ independent molecules in the characteristic dimer, while the other CBZ molecule is only hydrogen bonded to CBZ. In combination with these characteristics, the tri­fluoro­ethanol molecules form hydrogen bonds between each other along the channels which, in addition, are almost interconnected to form layers.

These factors may contribute to the unexpected results observed in the case of the TFE 1:1 and 2:1 solvates. According to the structure analysis, both solvates present a certain degree of similarity and CBZ Form I would be the expected polymorphic outcome of desolvation. On this basis, it was hypothesized that the 2:1 solvate would be an intermediate in the desolvation of the 1:1 crystal form. Yet, the results have shown that the solvate with the higher stoichiometry transforms directly to CBZ polymorph IV as the major phase, while the lower stoichiometric form gives polymorph I. It is suggested that the outcome of CBZ·TFE desolvation is kinetically driven, as the solvent release promotes a rapid collapse of the lattice before a 2:1 intermediate can be formed.

By way of contrast with the TFE solvates, in the case of the 1:1 and 2:1 BZQ cocrystals, the 2:1 stoichiometric form was shown to be an intermediate of the sublimation process of the 1:1 form, and the product, therefore, resulted in CBZ Form I. Although the interaction of CBZ and BZQ in the 1:1 cocrystal is as strong as in those CBZ multicomponent forms which showed the formation of a liquid phase, the lattice in CBZ·BZQ does not show evidence of such a collapse. It is suggested that the existence of an intermediate stable form, which provides stronger CBZ stacking interactions, coupled with the thermal behaviour of BZQ, affects the decomposition outcome of CBZ·BZQ.

In the case of CBZ·2H_2_O, although a few studies in the literature have suggested the presence of a monohydrate form (McMahon *et al.*, 1996 ▸; Surana *et al.*, 2003 ▸; Khoo *et al.*, 2013 ▸), in none of the experiments was such an intermediate observed. It was noted, however, that depending on the particle size, the dihydrate crystals can undergo complete dehydration and then further recrystallize into anhydrous CBZ, or show simultaneous dehydration and recrystallization events. The results highlight the influence of experimental conditions on the kinetics of water loss and lattice rearrangement during dehydration. The observation is also consistent with the results by Kachrimanis & Griesser (2012 ▸), who reported that dehydration occurs in two distinguishable steps not related to a monohydrate but representing the kinetics of the reaction.

### 4.4. Chemical decomposition as a function of the rate of transformation   

CBZ·TFA and 2CBZ·OXA clearly resulted in molten and decomposed material after sublimation/desolvation. Chemical degradation beyond solvent loss is suggested by the weight-loss curve (although the resulting product has not been identified). In the case of CBZ·TFA, the heating rate is found to drive the behaviour of the material upon heating either towards desolvation or to chemical decomposition. It is suggested that TFA *per se*, or the species formed during the decomposition of TFA, may react with CBZ, although the exact mechanism involved in these reactions is unclear. The literature reports that TFA thermally decomposes mainly into carbon dioxide, di­fluoro­methyl tri­fluoro­acetate, carbon mon­oxide and tri­fluoro­acetyl fluoride (Blake & Pritchard, 1967 ▸; Franciscot, 1992 ▸). Different studies have also shown the decomposition of salts of tri­fluoro­acetic acid and the use of this solvent as an ionizing agent promoting the degradation of various compounds under ambient conditions and at high temperatures (Blake & Shraydeh, 1981 ▸; Sundberg & Sloan, 1973 ▸; Canning *et al.*, 1999 ▸). We therefore hypothesize that high heating rates change the thermal behaviour of the CBZ·TFA solvate because they shift the desolvation to higher temperatures at which TFA may decompose and/or the chemical reaction of host and guest may occur. Decomposition may also be facilitated in the molten state, which was experimentally observed for CBZ·TFA.

A slightly different behaviour was observed in 2CBZ·OXA. In this case, different heating rates did not affect the thermal behaviour of the cocrystal, but the chemical decomposition of OXA accelerated the decomposition of CBZ molecules in the melt. The catalytic effect of OXA on the rate of decomposition of other compounds has already been reported in the literature. It was previously demonstrated, for instance, that OXA accelerates the decomposition of *m*-nitro­per­oxy­benzoic acid, leading to fusion during decomposition at about 78 °C (Debenham & Owen, 1966 ▸). The study shows that melting in itself is not the reason for the acceleration in decomposition, although the authors were unclear whether the phenomenon was related to a direct reaction of OXA with *m*-nitro­per­oxy­benzoic acid, or if it was mediated by the formation of hydrogen bonds between the reactants. In the case of 2CBZ·OXA, it is proposed that the formation of different species in the chemical decomposition of OXA while held together with CBZ in the cocrystal plays a major role. The literature reports that OXA mainly decomposes into formic acid and carbon dioxide when heated above 130 °C (Wobbe & Noyes, 1926 ▸; Higgins *et al.*, 1997 ▸). As both reaction products are expected to be vapours at these temperatures, the decomposition of OXA may give rise to the observed cocrystal thermal behaviour, which can be misunderstood as simple melting with sublimation.

It is suggested that the differences between CBZ·TFA and 2CBZ·OXA (namely the effect of heating rate variation) may be a consequence of the intermolecular interactions between host and guest. In the particular case of CBZ·TFA, Eberlin *et al.* (2013 ▸) have investigated the protonation state of CBZ·TFA and concluded that the solvate is best described as a salt with the acidic proton located at the mid-point between the acid and base, and this may vary with temperature. It is then suggested that the ionic nature of the interaction between the lattice constituents is another factor which may have affected the thermal behaviour of the TFA solvate. In the case of salts, desolvation may result in point defects (*e.g.* Schottky defects), which are more destructive in nature because the units surrounding the defect tend to move to maintain the overall neutral charge in the lattice (Tilley, 2008 ▸; Kelly & Knowles, 2012 ▸). The rate of desolvation could affect the type and the quantity of such defects, and, in the CBZ·TFA example, the rate of desolvation may also modify the ionization character of the species. Perhaps the properties of the desolvate product, such as melting, sublimation and stability, may vary because of the ionization state during desolvation.

## 5. Stress-induced transformations in the context of pharmaceutical manufacturing   

Pharmaceutical manufacturing of drug products containing crystalline active pharmaceutical ingredients (APIs) involve multiple unit operations, including crystallization, filtration, drying, milling, blending, granulation and compression, to name but some. In each of these unit operations, energy is imparted on the crystals in various forms most prominently as mechanical and thermal energy (Chen *et al.*, 2014 ▸). It is well documented that the pharmaceutical unit processes can generate various types of defects in API crystals (Dialer & Kuesner, 1973 ▸; Saleki-Gerhardt *et al.*, 1994 ▸; Ward & Schultz, 1995 ▸; Koivisto *et al.*, 2006 ▸; Chan & Doelker, 1985 ▸). These crystal defects represent regions of higher disorder and higher energy relative to the average overall energy of the crystalline material (Zhang *et al.*, 2006 ▸). These high-energy regions can ultimately affect the subsequent process *per se*, as well as a number of important pharmaceutical properties of APIs, including dissolution rate (Tawashi, 1968 ▸; Burt & Mitchell, 1981 ▸), chemical stability (Byrn *et al.*, 1994 ▸, 2001 ▸; Shalaev *et al.*, 2002 ▸), mechanical properties (Wildfong *et al.*, 2006 ▸) and moisture sorption (Ahlneck & Zografi, 1990 ▸). The effect of temperature on inducing phase transformations, crystallinity changes and surface defects in carbamazepine multicomponent forms described above adds another significant contribution to the overall process-induced disorder challenge.

The interface free energy and kinetics may also contribute to the course of recrystallization concomitant to the guest evolution that takes place in desolvation phenomena. These factors are illustrated in the effect that experimental conditions, surface chemistry and roughness, particle morphology, crystal size and the presence of seeds (and other templating surfaces from excipients, for example) may have during manufacturing. Yet, what is the balance in the relationship of thermodynamics and kinetics affecting organic solids and their interactions with the environment? For instance, the removal of residual liquid from a pharmaceutical ingredient subjected to drying involves different types of liquid states, unbound and bound liquids, that are differently affected by thermodynamics and kinetics. The unbound liquid is adsorbed on the outer surface of the particles and is the first liquid to be removed (Aulton & Taylor, 2013 ▸; Griesser, 2006 ▸). The bound liquid, in turn, consists of structural or capillary solvent entrapped within particles, defects and/or cavities (Aulton & Taylor, 2013 ▸; Griesser, 2006 ▸; Brittain, 2009 ▸). While the release of capillary liquid is mainly influenced by kinetics and is removed through less robust and reproducible reactions, the release of liquid from the crystalline lattice results from a combination of factors, as discussed in the previous sections.

Successful manufacture of pharmaceuticals not only relies on the fundamental understanding of the phenomena governing the transformations described above, but also how they are affected by scale and equipment. Heat and mass transfer and heterogeneity therein, homogeneity of the material itself, variations in equipment design and performance all require detailed studies and adequate controls to ensure consistent manufacture across scales and sites. Even reversible solid–solid phase transitions can irredeemably affect certain bulk and surface features with implications for downstream processing and performance. Specifically, powder flow, wettability and compression profiles can all be impacted. In summary, the added complexity of stress-induced solid–solid conversions studied here further emphasizes the importance of a mechanistic understanding of the relevant phenomena driving the transformations across scales and the implications thereof downstream.

## 6. Concluding remarks and perspectives   

This study of 15 multicomponent CBZ solids identifies some degree of correlation between structural features and the outcome of thermal decomposition processes, but highlights that the overall picture is complex, even within this series of closely related materials. The product of decomposition is frequently affected by the physical properties of the guest, such as boiling point and reactivity. This may give rise to the impact of experimental conditions on the outcome of the reactions, especially when events such as recrystallization, chemical decomposition, solubilization and peritectic melting occur concomitantly.

The results are largely consistent with the ‘Rouen 96’ model, in which Petit & Coquerel (1996 ▸) stated that the polymorphic outcome of crystal decomposition depends on the destructiveness of the process. The authors later added particle size and defects to this model and attributed the formation of a product layer on the surface of large particles to transformations which follow no structural correlation between mother and daughter phases. In this case, the outer layer affects further release of the guest molecules and, as the temperature increases, these particles undergo a destructive transformation. The authors did not, however, discuss the influence of particle dissolution on the outcome.

Our findings for the CBZ system suggest that the formation of a liquid phase may significantly affect the results of de­solv­ation reactions either by the formation of a product layer on the surface or by dissolving the crystal. Knowing the difference between the onset of physical decomposition and the boiling/sublimation temperature of the guest material appears to be crucial in answering whether a peritectic might be formed. In cases where a liquid phase intermediate is likely to develop, the thermodynamic stability relationship between polymorphs at the specified temperature governs the course of the reactions. In cases which are not intermediated by a liquid phase, packing similarities between parent phases and the products may be good parameters to predict the transformation results.

An open question remains as to how – and if – the temperature-mediated increase in molecular vibration differently affects networks which are held together by hydrogen bonds or ionic interactions. To the best of our knowledge, the interplay between desolvation, Schottky defect formation and chemical decomposition are unexplored in molecular crystals and no examples were found in the literature. Although these phenomena are difficult to tackle experimentally, this could be an interesting field of study, especially considering that salts tend often to be hydrated or solvated.

Augmentation of the current understanding of stress-induced transformations in organic materials with computation of strain and stress generated by the increase in temperature could be highly informative. When combined with carefully designed experiments, these calculations could provide solid-state chemists and process engineers with a framework to explain the effect of experimental conditions on the outcome. This might be achieved by combining information on the effect of host–guest strength (and nature) of interactions, the host lattice energy and the defects caused by molecular migration. Such a comprehensive model has not been developed thus far. For the time being, careful crystal structure analysis, solid-state characterization of the materials subjected to stress-induced transformations and the comparison of the physical properties of the guest, as illustrated in the present work, are the main tools to estimate the outcome of such transformations.

## Supplementary Material

Crystal structure: contains datablock(s) I. DOI: 10.1107/S2052520620015437/rm5042sup1.cif

The contents are: (1) Experimental, (2) Crystal structure analysis, (3) Extended data from thermal decomposition studies, and (4) References used in the supporting information. DOI: 10.1107/S2052520620015437/rm5042sup3.pdf

Structure factors: contains datablock(s) I. DOI: 10.1107/S2052520620015437/rm5042Isup2.hkl

CCDC reference: 2013467

## Figures and Tables

**Table 1:** Summary of the multicomponent CBZ forms in this study See text and Fig. 2 ▸ for a description of motifs **A**/**B**/B*. The CSD refcodes were obtained from the Cambridge Structural Database (Groom *et al.*, 2016 ▸).

Group	Motif	Structure	Coformer	CCDC number/refcode	Reference
1	**B**	CBZ·ACE	Acetone	CRBMZA01	Fleischman *et al.* (2003 ▸)
	**B**	CBZ·DMSO	Di­methyl­sulfoxide	UNEYIV01	Cruz-Cabeza *et al.* (2011 ▸)
	**B**	CBZ·DMF	*N*,*N*-Di­methyl­formamide	QANQUS	Johnston *et al.* (2005 ▸)
2	B*	CBZ·DMA	*N,N*-Di­methyl­acetamide	KIWBEY	Johnston *et al.* (2008 ▸)
	B*	CBZ·BZQ	Benzo­quinone	QABHIO	Schneider Rauber *et al.* (2021 ▸)
3	**A**	2CBZ·OXA	Oxalic acid	MOXWUS	Childs *et al.* (2009 ▸)
	**A**	CBZ·2H_2_O	Water	FEFNOT02	Harris *et al.* (2005 ▸)
	**A**	2CBZ·BZQ	Benzo­quinone	UNEYOB	Fleischman *et al.* (2003 ▸)
	**A**	CBZ·FORM	Formamide	UNIBOI	Fleischman *et al.* (2003 ▸)
	**A**	2CBZ·DIOX	1,4-Dioxane	QABHOU	Schneider Rauber *et al.* (2021 ▸)
4	**A**	CBZ·TFE	2,2,2-Tri­fluoro­ethanol	SAPDUJ	Lohani *et al.* (2005 ▸)
	**A**	2CBZ·TFE	2,2,2-Tri­fluoro­ethanol	2013467	This work
5	**A**	CBZ·FA	Formic acid	UNEZOC	Fleischman *et al.* (2003 ▸)
	**A**	CBZ·ACA	Acetic acid	UNEZIW	Fleischman *et al.* (2003 ▸)
	**A**	CBZ·TFA	Tri­fluoro­acetic acid	GINFOZ	Fernandes *et al.* (2007 ▸)

Isostructural sets: {CBZ·ACE, CBZ·DMSO}; {2CBZ·OXA, CBZ·2H_2_O, 2CBZ·BZQ}; {CBZ·FA, CBZ·ACA}; {CBZ·FORM, 2CBZ·DIOX}.

**Table 2:** Comparison of the CBZ–coformer intermolecular interactions in the CBZ multicomponent materials The hydrogen-bond (HB) geometry refers to normalized H-atom positions. *E* ^AB^ values are calculated using the UNI forcefield in *Mercury* (Macrae *et al.*, 2020 ▸).

		HB_CBZ→coformer_			
Group	Structure	Type	*d*(H⋯*A*) (Å), θ(*X*—H⋯*A*) (°)	*E* ^AB^_CBZ→coformer_ (kJ mol^−1^)	*E* ^AB^_coformer→coformer_^6^ (kJ mol^−1^)
1	CBZ·ACE	N—H_CBZ_⋯O	2.17, 139.9	−25.4	−8.1
	CBZ·DMSO^1^	N—H_CBZ_⋯O	2.19, 143.6	−27.9	−8.5
	CBZ·DMF	N—H_CBZ_⋯O	1.88, 153.3	−34.0	−5.5
2	CBZ·DMA^1^	N—H_CBZ_⋯O	1.91, 164.3	−41.5	−5.7
	CBZ·BZQ	N—H_CBZ_⋯O	2.03, 156.1	−35.2	−9.9
3	2CBZ·BZQ	N—H_CBZ_⋯O	2.21, 148.0	−24.2	−9.4
	2CBZ·OXA^2^				
	CBZ·2H_2_O	N—H_CBZ_⋯O	2.23, 142.4	−16.2	−17.9 (hydrogen bonded)
		O—H⋯O_CBZ_	1.88, 159.6	−25.3	
	CBZ·FORM	N—H_CBZ_⋯O	2.11, 141.9	−20.8	−33.3 (hydrogen bonded)
		N—H⋯O_CBZ_	1.95, 150.2	−24.0	
		N—H_CBZ_⋯O	2.03, 143.7	−23.7	
		N—H⋯O_CBZ_	1.93, 171.9	−26.3	
	2CBZ·DIOX^3^	N—H_CBZ_⋯O	2.16, 130.7	−27.6	−10.7
		N—H_CBZ_⋯O	2.43, 115.1	−18.8	
4	CBZ·TFE	N—H_CBZ_⋯O	2.34, 122.2	−15.9	−18.9 (hydrogen bonded)
		O—H⋯O_CBZ_	1.65, 168.3	−34.9	
	2CBZ·TFE^3,4^	N—H_CBZ_⋯O	2.16, 133.6	−18.9	−22.4 (hydrogen bonded)
		O—H⋯O_CBZ_	1.80, 179.6	−35.5	
5	CBZ·TFA^5^	N—H_CBZ_⋯O	1.90, 164.7		
		O—H⋯O_CBZ_	1.45, 172.1		
		N—H_CBZ_⋯O	2.20, 130.8		
	CBZ·ACA	N—H_CBZ_⋯O	2.09, 150.7	−38.2	−3.2
		O—H⋯O_CBZ_	1.58, 166.9		
		N—H_CBZ_⋯O	2.25, 122.3	−16.4	
	CBZ·FA	N—H_CBZ_⋯O	2.02, 150.0	−38.4	−1.5
		O—H⋯O_CBZ_	1.57, 166.7		
		N—H_CBZ_⋯O	2.05, 138.6	−22.6	

Notes: (1) Structure contains disorder of the DMSO/DMA molecules. The major disorder component is considered. (2) Values for 2CBZ·OXA are not provided because the oxalic acid molecules are omitted in the reported structure (Childs *et al.*, 2009 ▸). See Table S2 for details. (3) Structure contains positional disorder of the DIOX/TFE molecules, which can be eliminated by constructing a supercell. The *E* ^AB^ values are calculated from the supercell model. (4) One set of CBZ dimers does not hydrogen bond to the TFE molecules. (5) The UNI forcefield produces unusual values for CBZ·TFA on normalization of the H-atom positions, which must be attributable to the short H⋯O hydrogen bond (1.45 Å). Without normalization, the interaction energy between CBZ and TFA is comparable to CBZ·ACA and CBZ·FA. (6) *E* ^AB^_coformer→coformer_ expresses the intermolecular energy between the two closest coformer molecules.

**Table 3:** Quantitative data obtained from thermal analyses of CBZ multicomponent materials

		Decomposition^1^			
Material	Sublimation/boiling *T* (°C)^2^	*T*_onset_ (°C)	*T*_peak_ (°C)	ΔH (kJ mol^−1^)	Weight loss (wt%)
CBZ·ACE	56	79.8 ± 2.9	87.3 ± 4.2	48.7 ± 1.7	19.6 ± 0.4
CBZ·DMSO	189	100.8 ± 1.1	106.7 ± 1.3	56.8 ± 1.2	24.6 ± 0.3
CBZ·DMF	153	77.1 ± 0.3	80.2 ± 0.1	53.9 ± 0.9	23.2 ± 0.4
CBZ·DMA	165	76.6 ± 2.0	79.5 ± 4.5	62.5 ± 1.4	26.8 ± 1.4
CBZ·BZQ	124	146.2 ± 13.6	159.9 ± 9.2	82.5 ± 4.0	31.3 ± 0.3
2CBZ·BZQ	124	155.1 ± 12.6	167.3 ± 2.8	93.2 ± 3.6	18.7 ± 0.1
2CBZ·OXA^3^	157	156.3 ± 1.4	160.2 ± 0.6	58.8 ± 0.8	
CBZ·2H_2_O (1)^4^	100	66.1 ± 0.2	75.7 ± 1.7	78.3 ± 2.3	13.2 ± 0.1
CBZ·2H_2_O (2)^4^	100	85.8 ± 0.1	92.4 ± 0.2	81.2 ± 0.5	13.0 ± 0.3
CBZ·FORM	210	144.5 ± 0.1	147.3 ± 1.8	50.7 ± 3.8	16.9 ± 0.4
2CBZ·DIOX	100	91.7 ± 1.2	98.7 ± 1.1	48.6 ± 1.2	15.1 ± 0.7
CBZ·TFE	77	68.4 ± 0.7	78.1 ± 1.0	51.0 ± 1.7	29.7 ± 0.4
2CBZ·TFE	77	97.2 ± 0.4	108.1 ± 3.4	50.9 ± 0.1	16.9 ± 0.1
CBZ·TFA^3^	72	128.4 ± 2.0	134.2 ± 0.1	104.8 ± 1.0	
CBZ·TFA^5^	72	120.5 ± 5.2	124.8 ± 0.7	72.6 ± 0.7	33.0 ± 0.9
CBZ·ACA	117	125.6 ± 0.2	139.6 ± 0.1	63.3 ± 3.9	21.1 ± 0.8
CBZ·FA	100	119.0 ± 0.1	124.8 ± 1.1	57.1 ± 0.4	16.3 ± 0.2

Notes: (1) DSC: 10 °C min^−1^, perforated lid, N_2_; TGA: 10 °C min^−1^, open pan, N_2_. (2) According to supplier’s information. (3) Event occurs simultaneously with the chemical decomposition of CBZ. (4) CBZ·2H_2_O (1) and (2) are samples that differ with respect to their morphology (*i.e.* they present different dominant crystal surfaces and particle size). See §S1.2 and §S3.7 for additional information. (5) DSC: 1 °C min^−1^, perforated lid, N_2_; TGA: 1 °C min^−1^, open pan, N_2_.
