# Supplementary material for: Polymorphism and surface diversity arising from stress-induced transformations – the case of multicomponent forms of carbamazepine
Source: Acta Crystallogr B Struct Sci Cryst Eng Mater. 2021 Jan 16;77(Pt 1):54–67. doi: 10.1107/S2052520620015437 (PMC7941281; doi:10.1107/S2052520620015437)
Supplement: Supplementary file 2 [file b-77-00054-sup3.pdf]

STRUCTURAL SCIENCE
CRYSTAL ENGINEERING
MATERIALS

Volume 77 (2021)

Supporting information for article:

Polymorphism and surface diversity arising from stress-induced transformations – the case of multicomponent forms of carbamazepine

Gabriela Schneider-Rauber, Mihails Arhangelskis, Andrew D. Bond, Raimundo Ho, Nandkishor Nere, Shailendra Bordawekar, Ahmad Sheikh and William Jones

Polymorphism and surface diversity arising from stress-induced transformations – the case of multicomponent forms of carbamazepine

Gabriela Schneider-Rauber, Mihails Arhangelskis, Andrew D. Bond, Raimundo Ho, Nandkishor Nere, Shailendra Bordawekar, Ahmad Sheikh, William Jones.

Contents

S1. Experimental 3

S1.1. Materials..3

S1.2. Solution crystallization 3

S1.3. Single-crystal X-ray diffraction 4

S1.4. Use of the Cambridge Structural Database (CSD)..5

S1.5. Powder X-ray diffraction (PXRD)..5

S1.6. Surface analyses..5

S1.7. Thermal analyses..6

S1.8. Attenuated total reflection Fourier transform infrared spectroscopy (ATR-FTIR)..6

S2. Crystal structure analysis applied to stress-induced transformations..7

S3. Data from thermal decomposition studies of carbamazepine multicomponent forms..16

S3.1. CBZ acetone solvate (CBZ·ACE), Group 1..16

S3.2. CBZ N,N-dimethylsulfoxide solvate (CBZ·DMSO), Group 1 18

S3.3. CBZ N,N-dimethylformamide solvate (CBZ·DMF), Group 1 21

S3.4. CBZ N,N-dimethylacetamide solvate (CBZ·DMA), Group 2..24

S3.5. CBZ 1,4-benzoquinone cocrystals (CBZ·BZQ and 2CBZ·BZQ), Group 2 and Group 3............27

S3.6. CBZ oxalic acid cocrystal (2CBZ·OXA), Group 3..32

S3.7. CBZ dihydrate (CBZ·2H_2O), Group 3..34

S3.8. CBZ formamide solvate (CBZ·FORM), Group 3..37

S3.9. CBZ dioxane solvate (2CBZ·DIOX), Group 3 39

S3.10. CBZ 2,2,2-trifluoroethanol solvates (CBZ·TFE and 2CBZ·TFE), Group 4..41

S3.11. CBZ trifluoroacetic acid solvate (CBZ·TFA), Group 5..46

S3.12. CBZ acetic acid solvate (CBZ·ACA), Group 5 50

S3.13. CBZ formic acid solvate (CBZ·FA), Group 5 53

S3.14. Additional thermal behaviour analyses on carbamazepine polymorphs I and III..56

S4. References..61

S1. Experimental

S1.1. Materials

Solvents were supplied by Sigma-Aldrich Company Ltd and used without further purification. Carbamazepine, benzoquinone and oxalic acid were obtained from Alfa Aesar, Thermo Fisher Scientific and Sigma Aldrich Ltd, respectively.

Table S1 Summary of crystallization conditions

Crystal form	Solvent composition (volume proportion)	Concentration (mg.mL^{-1})	Solubilizing temperature (°C)
CBZ·ACE	Acetone 100%	72	55
CBZ·DMF	DMF 100%	634	80
CBZ·DMSO	DMSO 100%	284	80
CBZ·DMA	DMA 100%	600	80
CBZ·BZQ	Ethanol 100%	20/147 (CBZ/BZQ)	70
2CBZ·BZQ	Ethanol 100%	70/15 (CBZ/BZQ)	70
2CBZ·OXA	Ethyl acetate 100%	27	70
CBZ·2H_2O (1)	Water:ethanol 75:25	7	70
CBZ·2H_2O (2)	Water:ethanol 25:75	70	70
CBZ·FORM	Formamide:ethanol 20:80	255	80
2CBZ·DIOX	1,4 dioxane:ethanol 35:65	453	80
CBZ·TFE	Trifluoroethanol 100%	925	70
2CBZ·TFE	Trfluoroethanol:ethanol 50:50	315	70
CBZ·TFA	Trifluoroacetic acid:ethanol 50:50	106	70
CBZ·ACA	Acetic acid:ethanol 25:75	112	80
CBZ·FA	Formic acid 100%	635	80

S1.2. Solution crystallization

Solution crystallization based on the cooling technique was used. The aim was to preserve the activity of the solvents throughout the crystallization process and to avoid the presence of seeds and extensive defects on the materials which were further used to study stress-induced transformations. Clear

solutions were prepared on a hot plate by stirring CBZ in the solvent and were subsequently cooled (5 ^{o}C/h) in a bead bath. The crystals were allowed to spontaneously nucleate (*i.e.* without the addition of seeds) and no agitation was used to avoid particle breakage. The crystals were harvested by vacuum filtration and dried under room conditions.

Table S1 shows a summary of the crystallization conditions used. The concentration of the crystallization solutions was defined in relation to the supersaturation degree of the solid phase in the respective solution. The solution composition was selected based on previous studies (unpublished results) and aimed to provide crystals with desired size and surface characteristics. The conditions were defined from equilibrium solubility measurements. The cocrystals of carbamazepine with benzoquinone and oxalic acid were prepared according to the literature (Fleischman *et al.*, 2003; Childs *et al.*, 2009).

The screening of appropriate crystallization conditions has also resulted in two new structures of CBZ multicomponent crystal forms already reported in different stoichiometries. The first structure in the CBZ/TFE system was obtained by slow evaporation of a 2,2,2-trifluoroethanol solution and described by Lohani *et al.* (2005). It has a 1:1 stoichiometry and is reported in the CSD as SAPDUJ. In our screening studies, however, a 2:1 stoichiometry was also crystallized in ethanol:trifluoroethanol (its structure is reported in the present study). In the case of CBZ/BZQ, a cocrystal was first described by Fleishman *et al.* (2003) with a 2:1 stoichiometry. The 2CBZ:BZQ cocrystal is reported in the CSD as UNEYOB and was initially obtained by slow solvent evaporation starting from a 1:1 proportion of CBZ and BZQ (the solvent used was not reported). In our screening studies, however, a 1:1 stoichiometry was also crystallised in ethanol at high proportions of BZQ, following the reactive crystallization method proposed by Rodríguez-Hornedo and co-workers (Nehm *et al.*, 2006; Good & Rodríguez-Hornedo, 2010; Lipert & Rodríguez-Hornedo, 2015). Later, CBZ:BZQ was also obtained from cooling and other slurry experiments, always with high quantities of unreacted BZQ. The structure has been reported elsewhere (Schneider Rauber *et al.*, 2020*c*).

S1.3. Single-crystal X-ray diffraction

Single crystal X-ray diffraction data for 2CBZ·TFE were collected on a Nonius KappaCCD diffractometer (MoKα radiation; $\lambda = 0.7107$ Å). The temperature was maintained at 180 K using an Oxford Cryostream open-flow nitrogen device. Integration was carried out using HKL Denzo/Scalepack, and a multi-scan absorption correction was applied using SORTAV. Crystallographic data are summarized in Section S2 (**Table S2**) and CIF files are provided as Supporting Information or can be retrieved from the Cambridge Crystallographic Data Centre (CCDC 2013467).

S1.4. Use of the Cambridge Structural Database (CSD)

Conquest was used to search for deposited crystal structures. Mercury was used for general structural analysis and visualization as well as obtaining crystal packing similarities between structures, simulated PXRD patterns, analysis of voids and values of intermolecular energy calculated using the UNI force-field potentials. The refcodes of the crystal structures retrieved from the CSD are as follows: carbamazepine Form I (CBMZPN11), Form III (CBMZPN01) and Form IV (CBMZPN12), CBZ·ACE (CRBMZA01), CBZ·DMF (QANQUS), CBZ·DMSO (UNEYIV01), CBZ·DMA (KIWBEY), CBZ·BZQ (1994550), 2CBZ·BZQ (UNEYOB), 2CBZ·OXA (MOXWUS), CBZ·2H_2O (FEFNOT02), CBZ·FORM (UNIBOI), 2CBZ·DIOX (1994551), CBZ·TFE (SAPDUJ), 2CBZ·TFE (2013467), CBZ·TFA (GINFOZ), CBZ·ACA (UNEZIW) and CBZ·FA (UNEZOC).

S1.5. Powder X-ray diffraction (PXRD)

PXRD measurements were performed at room temperature on a PanAlytical X'pert PRO Multi-Purpose Diffractometer using non-monochromated CuKα radiation (λ = 1.5418 Å) and an X'Celerator detector. The X-ray generator was set at a voltage of 40 kV and current of 40 mA. Samples were placed onto a flat glass slide and scanned from 3° to 40° 2θ using a total scanning time of 5 minutes.

Experiments with modulated temperatures used an Anton Paar TTK 450 temperature air open chamber. Samples were evaluated with different temperature gradients: non-isothermal runs at heating rate of 10 ºC.min^{-1} (one 2 minutes scan every 10ºC), non-isothermal runs at heating rate of 1 ºC.min^{-1} (one 2 minute scan every 5 ºC).

S1.6. Surface analyses

Optical micrographs (OM) were recorded with a Leica DM1000 LED transmission optical microscope (polarizing filter) equipped with a JVC colour video camera. The microscope was also used for recording images during hot stage experiments. The heating programs were made in a Metler Toledo FP90 Central Processor connected to a FP84HT TA Microscopy Cell. The samples were prepared in 7mm Ø opened glass pans and scanned at heating rate of 10 K.min^{-1}, under open atmosphere. The data was processed using Studio software.

Scanning electron micrographs (SEM) of fresh and thermally processed (see **Section S1.7**) samples were also taken. The equipment used was a JEOL JSM-5510LV scanning electron microscope. The samples were mounted with adhesive tape over an aluminium holder and sputtered with gold for 3 minutes (Agar Sputter Coater). The vacuum within the sputter unit was ca 1 x 10^{-3} torr.

S1.7. Thermal analyses

Differential scanning calorimetry (DSC) was carried out using a Mettler Toledo DSC822 instrument. Samples weighing 1.0-4.0 mg were heated in 40 μL pin-holed closed pans and scanned at heating rate of 1 or 10 K.min^{-1}, under nitrogen air atmosphere (80 mL.min^{-1}).

The mass loss of the samples as a function of temperature was determined by thermogravimetric analysis (TGA) using a Mettler Toledo TGA/SDTA851e/SF/1100 instrument. Typically, 2-10 mg were placed in open 100 μL aluminium crucibles at a heating rate of 1 or 10 K.min^{-1}, under nitrogen atmosphere (80 mL.min^{-1}). The obtained thermal data were processed using Metler STARe software. The thermobalance was also used to prepare thermally processed samples prior to SEM surface analysis.

S1.8. Attenuated total reflection Fourier transform infrared spectroscopy (ATR-FTIR)

Spectra were collected on a ThermoNicolet Nexus FT-IR spectrometer with a Smart Golden Gate Single Reflection ATR accessory. Samples were measured from (500-4000) cm^{-1} at a resolution of 4 cm^{-1} and 32 scans.

S2. Crystal structure analysis applied to stress-induced transformations

Table S2 Crystallographic data of CBZ materials studied and/or mentioned in the article.

Material	CBZ·ACE	CBZ·DMSO[a/b]	CBZ·DMSO[a/b]	CBZ·DMF	CBZ·DMA[a]
Formula	$(C_{15}H_{12}N_2O)$ (C_3H_6O)	$(C_{15}H_{12}N_2O)$ (C_2H_6OS)	$(C_{15}H_{12}N_2O)$ (C_2H_6OS)	$(C_{15}H_{12}N_2O)$ (C_3H_7NO)	$(C_{15}H_{12}N_2O)$ (C_4H_9NO)
MW/g.mol^{-1}	294.35	314.40	314.40	309.36	323.39
T/K	100	110	280	123	123
Crystal system	Triclinic	Triclinic	Triclinic	Triclinic	Monoclinic
Space group	*P*-1	*P*-1	*P*-1	*P*-1	$P2_1/c$
a/Å	7.3985(15)	8.8708(2)	7.4105(3)	7.7118(4)	7.5046(7)
b/Å	8.9054(18)	12.1901(3)	8.9753(3)	9.1503(4)	19.506(2)
c/Å	11.873(2)	14.6128(4)	12.2407(4)	11.6969(6)	11.7808(13)
α/º	95.809(3)	94.9700(10)	94.259(2)	100.192(3)	90
β/º	93.401(4)	100.2170(10)	94.526(3)	95.379(2)	96.597(8)
γ/º	101.362(3)	95.0360(10)	100.200(2)	101.908(3)	90
V/Å^3	760.479	1540.67	795.544	787.584	1713.11
Z/Z'	2/1	4/2	2/1	2/1	4/1
ρ/g.cm^{-3}	1.285	1.355	1.313	1.305	1.254
R-factor/%	7.68	4.75	4.86	4.77	6.76
CSD refcode	CRBMZA01	UNEYIV04	UNEYIV01	QANQUS	KIWBEY
Reference	(Fleischman *et al.*, 2003)	(Cruz-Cabeza *et al.*, 2011)	(Cruz-Cabeza *et al.*, 2011)	(Johnston *et al.*, 2005)	(Johnston *et al.*, 2008)

(to be continued next page)

[a] Structure presents disorder

[b] These structures differ only in the extent of disorder of the DMSO solvent molecules. The positions of the carbamazepine molecules are identical.

Material	CBZ·BZQ	2CBZ·BZQ	CBZ/OXA[a]	CBZ·2H_2O
Formula	$(C_{15}H_{12}N_2O)$ $(C_6H_4O_2)$	$(C_{15}H_{12}N_2O)_2$ $(C_6H_4O_2)$	$(C_{15}H_{12}N_2O)$ $(C_2H_2O_4)_x$	$(C_{15}H_{12}N_2O)$ $(H_2O)_2$
MW/g.mol^{-1}	344.36	580.63	326.30	272.30
T/K	180	100	173	120
Crystal system	Triclinic	Monoclinic	Monoclinic	Monoclinic
Space group	*P*-1	$P2_1/c$	$P2_1/c$	$P2_1/c$
a/Å	7.0891(2)	10.3335(18)	10.330(14)	10.066(2)
b/Å	10.2587(3)	27.611(5)	26.24(3)	28.719(5)
c/Å	12.6557(3)	4.9960(9)	5.110(6)	4.831(1)
α/º	80.435(2)	90	90	90
β/º	86.6170(10)	102.275(3)	104.21(3)	103.45(1)
γ/º	71.0080(10)	90	90	90
V/Å^3	858.172	1392.86	1342.73	1358.27
Z/Z’	2/1	2/0.5	2/0.5	4/1
ρ/g.cm^{-3}	1.333	1.384	1.614	1.332
R-factor/%	3.58	5.45	14.08	7.29
CSD refcode	1994550	UNEYOB	MOXWUS	FEFNOT02
Reference	(Schneider Rauber *et al.*, 2020*c*)	(Fleischman *et al.*, 2003)	(Childs *et al.*, 2009)	(Harris *et al.*, 2005)

(to be continued next page)

Material	CBZ·FORM	2CBZ·DIOX[a]	2CBZ·DIOX[b]	CBZ·TFE	2CBZ·TFE[a]
Formula	$(C_{15}H_{12}N_2O)$ (CH_3NO)	$(C_{15}H_{12}N_2O)_2$ $(C_4H_8O_2)$	$(C_{15}H_{12}N_2O)_2$ $(C_4H_8O_2)$	$(C_{15}H_{12}N_2O)$ $(C_2H_3F_3O)$	$(C_{15}H_{12}N_2O)_2$ $(C_2H_3F_3O)$
MW/g.mol^{-1}	281.31	560.63	560.63	336.31	572.58
T/K	100	180	180	173	180
Crystal system	Triclinic	Monoclinic	Monoclinic	Triclinic	Triclinic
Space group	*P*-1	$P2_1/n$	$P2_1/c$	*P*-1	*P*-1
a/Å	5.1077(11)	16.7776(16)	28.071(2)	10.236(2)	5.2419(2)
b/Å	16.057(3)	4.9175(5)	4.917(4)	12.937(2)	15.0825(5)
c/Å	17.752(4)	17.991(17)	20.5138(14)	13.614(2)	18.4638(6)
α/°	73.711(3)	90	90	62.419(2)	103.874(2)
β/°	89.350(3)	107.814(5)	94.177(4)	88.218(2)	93.756(2)
γ/°	88.636(3)	90	90	84.286(2)	97.404(2)
V/Å^3	1397.06	1413.2(2)	2823.96	1589.76	1398.3
Z/Z’	4/2	2/0.5	4/1	4/2	4/2
ρ/g.cm^{-3}	1.337	1.318	1.319	1.405	1.360
R-factor/%	7.66	7.27	10.9	4.64	8.18
CSD refcode	UNIBOI	1994551	1994552	SAPDUJ	2013467
Reference	(Fleischman *et al.*, 2003)	(Schneider Rauber *et al.*, 2020*a*)	(Schneider Rauber *et al.*, 2020*b*)	(Lohani *et al.*, 2005)	This work

(to be continued next page)

[a] Structure presents disorder

[b] This model has been refined as a super cell, to eliminate positional disorder of the dioxane molecules.

Material	CBZ·TFA[a]	CBZ·ACA	CBZ·FA
Formula	$(C_{15}H_{12}N_2O)$ $(C_2HF_3O_2)$	$(C_{15}H_{12}N_2O)$ $(C_2H_4O_2)$	$(C_{15}H_{12}N_2O)$ (CH_2O_2)
MW/g.mol^{-1}	350.29	296.32	282.32
T/K	150	100	100
Crystal system	Monoclinic	Monoclinic	Monoclinic
Space group	$P2_1$/n	$P2_1/c$	$P2_1/c$
a/Å	15.0142(4)	5.1206(4)	5.2031(9)
b/Å	5.28710(10)	15.7136(13)	14.741(2)
c/Å	20.2489(6)	18.4986(15)	17.882(3)
α/º	90	90	90
β/º	101.735(3)	96.5460(10)	98.132(3)
γ/º	90	90	90
V/Å^3	1573.79	1478.75	1357.74
Z/Z'	4/1	4/1	4/1
ρ/g.cm^{-3}	1.478	1.331	1.381
R-factor/%	3.86	4.92	5.33
CSD refcode	GINFOZ	UNEZIW	UNEZOC
Reference	(Fernandes *et al.*, 2007)	(Fleischman *et al.*, 2003)	(Fleischman *et al.*, 2003)

[a] Structure presents disorder

Material	CBZ I	CBZ II	CBZ III	CBZ IV	CBZ V
Formula	$C_{15}H_{12}N_2O$	$C_{15}H_{12}N_2O$	$C_{15}H_{12}N_2O$	$C_{15}H_{12}N_2O$	$C_{15}H_{12}N_2O$
MW/g.mol^{-1}	236.27	236.27	236.27	236.27	236.27
T/K	158	298	298	158	123
Crystal system	Triclinic	Trigonal	Monoclinic	Monoclinic	Orthorhombic
Space group	*P*-1	*R*-3	$P2_1/c$	*C*2/*c*	*Pbca*
a/Å	5.1705(6)	35.454(3)	7.529(1)	26.609(4)	9.1245(5)
b/Å	20.574(2)	35.454(3)	11.148(2)	6.9269(10)	10.4518(5)
c/Å	22.245(2)	5.253(1)	15.470(2)	13.957(2)	24.8224(11)
α/°	84.124(4)	90	90	90	90
β/°	88.008(4)	90	116.17(1)	109.702(2)	90
γ/°	85.187(4)	120	90	90	90
V/Å^3	2344.82	5718.32	1165.34	2421.93	2367.25
Z/Z’	8/4	18/1	4/1	8/1	8/1
ρ/g.cm^{-3}	1.339	1.235	1.347	1.296	1.326
R-factor/%	5.06	6.9	3.5	3.57	4.5
CSD refcode	CBMZPN11	CBMZPN03	CBMZPN01	CBMZPN12	CBMZPN16
Reference	(Grzesiak *et al.*, 2003)	(Lowes *et al.*, 1987)	(Reboul *et al.*, 1981)	(Lang *et al.*, 2002)	(Arlin *et al.*, 2011)

Table S3 Coformer virtual cavity analysis of the CBZ multicomponent materials. Data from Mercury®, CSD.

Group	Structure	Cavities/area (Å^{-2})[2]	Cavities/volume (Å^{-3})[3]	Cavities/volume normalized (x10^{-5})[4]	Volume% of cavities[5]	Weight% of coformer
1	CBZ·ACE	0.011 / 0.010	0.0026	1.73	20.0	19.73
	CBZ·DMSO	0.011 / 0.009	0.0025	1.23	25.7	24.85
	CBZ·DMF	0.011 / 0.009	0.0025	1.65	19.5	23.63
2	CBZ·DMA	0.014	0.0012	0.26	25.9	26.94
	CBZ·BZQ	0.014	0.0012	0.42	32.0	31.39
3	2CBZ·BZQ	0.007	0.0014	0.54	19.2	18.62
	2CBZ·OXA	0.007	0.0015	0.73	15.7	16.00
	CBZ·2H_2O	0.007	0.0015	0.56	19.2	13.23
	CBZ·FORM	0.007	0.0014	0.65	15.8	16.01
	2CBZ·DIOX	0.007	0.0014	0.60	16.8	15.72
4	CBZ·TFE	0.006	0.0006	0.15	26.0	29.75
	2CBZ·TFE[1]	0.004	0.0007	0.32	16.0	17.47
5	CBZ·TFA	0.007	0.0013	0.35	23.0	32.55
	CBZ·ACA	0.014	0.0027	1.08	16.9	20.27
	CBZ·FA	-	0.0029	2.32	9.4	16.30

[1] Calculated from the doubled unit cell which presents TFE molecules in two possible positions (static disorder).

[2] Number of cavities per area of the unit cell's surface crossed by the cavities. Two faces are characterized in Group 1, as the structures present channels along two directions. The values in Group 1 are relative to the *ac* and *bc* faces, respectively. CBZ·FA was not characterized as the solvent is arranged in pockets.

[3] Number of cavities per volume of the unit cell.

[4] Number of cavities per volume of the unit cell divided by the volume occupied by the cavities.

[5] Virtual volume of the cavities calculated from the removal of the coformer molecules from the CIF files.

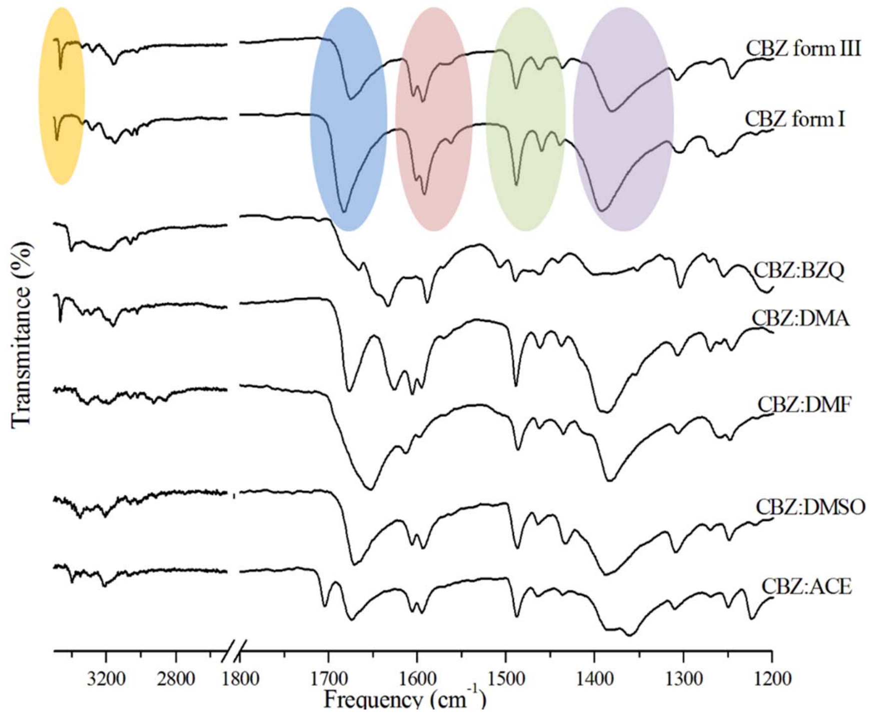

Figure S1 IR spectra of CBZ materials classified in Groups 1 and 2. The spectra of Forms I and III are added for comparison and have specific regions highlighted: N-H stretching (yellow); C=O stretching (blue); N-H bending (pink); C=C stretching (green); C-N stretching (purple).

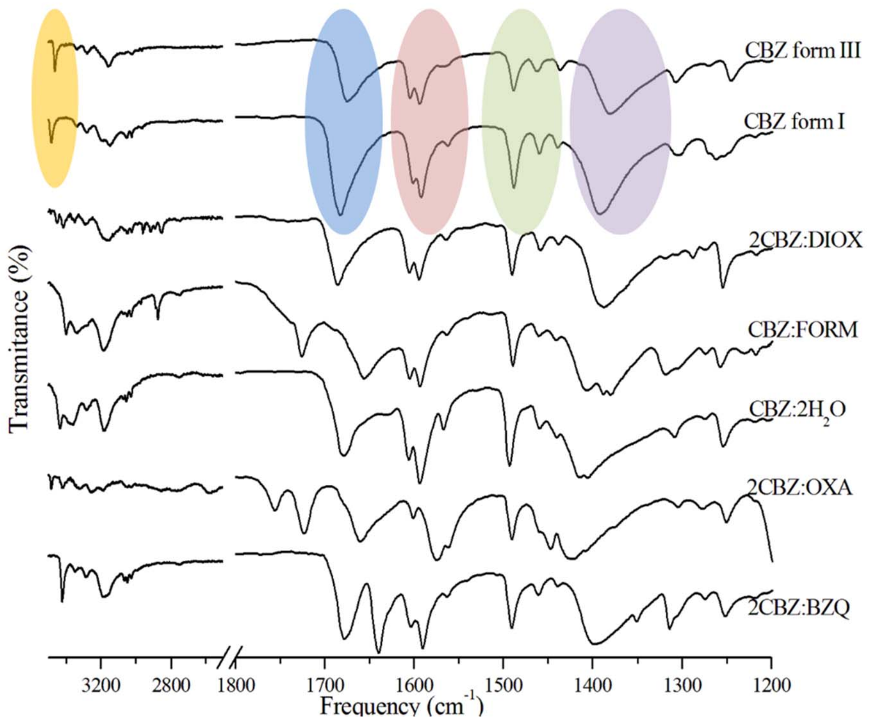

Figure S2 IR spectra of CBZ materials classified in Group 3.

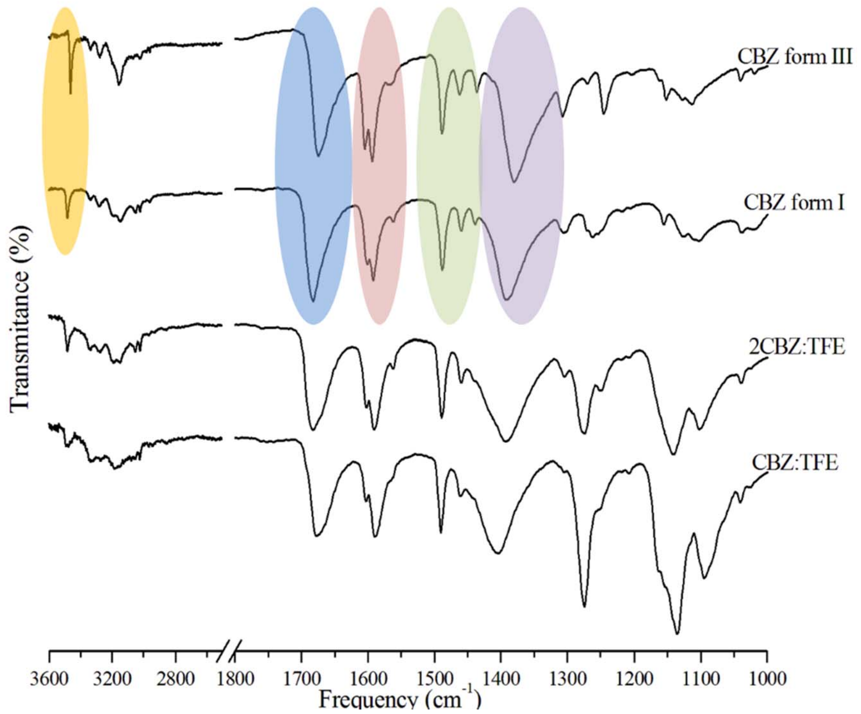

Figure S3 IR spectra of CBZ materials classified in Group 4.

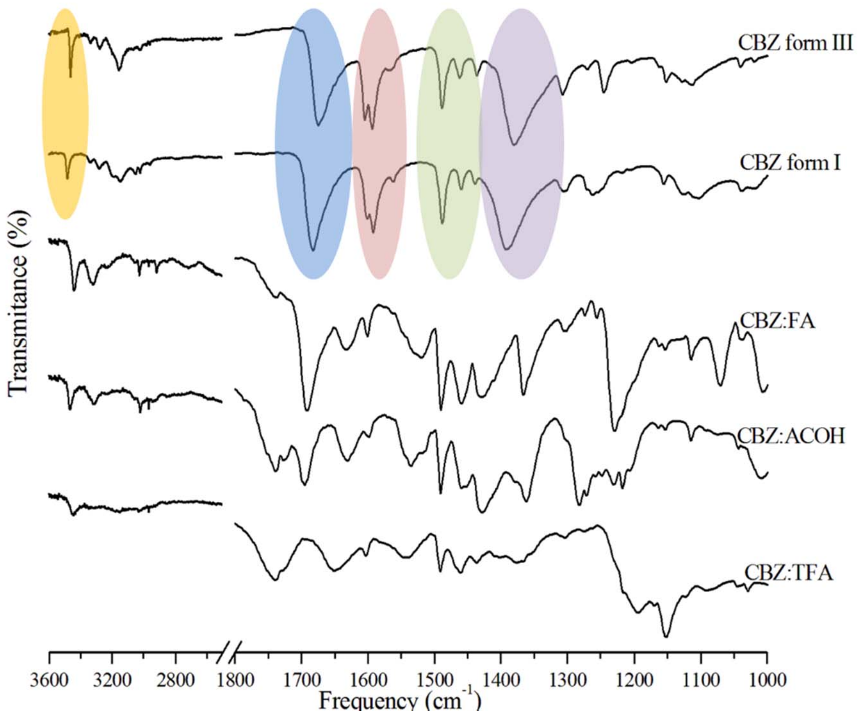

Figure S4 IR spectra of CBZ materials classified in Group 5.

S3. Data from thermal decomposition studies of carbamazepine multicomponent forms

S3.1. CBZ acetone solvate (CBZ·ACE), Group 1

The TGA curve shows one single weight loss ending at about 100 ºC and the heat flow curve demonstrates that the desolvation is characterized by a relatively sharp endothermic event (T_{onset}=79.8 ºC). Additional endotherms are also seen before the characteristic melting of CBZ Form I, which occurs above 190 ºC. The endothermic events in the range 160-180 ºC can be correlated to the transition of Form III to Form I, while the endothermic event starting at about 185 ºC resembles the melting of remaining Form III that has been described in the literature (Umeda *et al.*, 1984; Rustichelli *et al.*, 2000) and experimentally confirmed (see **Figure S78** and **Figure S79**).

It is noteworthy that the melting of Form III was not observable in every DSC experiment of CBZ·ACE. In the case of small crystals, only the solid-state transition of Form III to Form I is detected. It is suggested that CBZ·ACE desolvates to polymorph III, which may convert to polymorph I at higher temperatures. In larger crystals, this transformation is sometimes not complete when the melting temperature of Form III is reached. In this case, the remaining Form III melts and recrystallizes as Form I just before its melting.

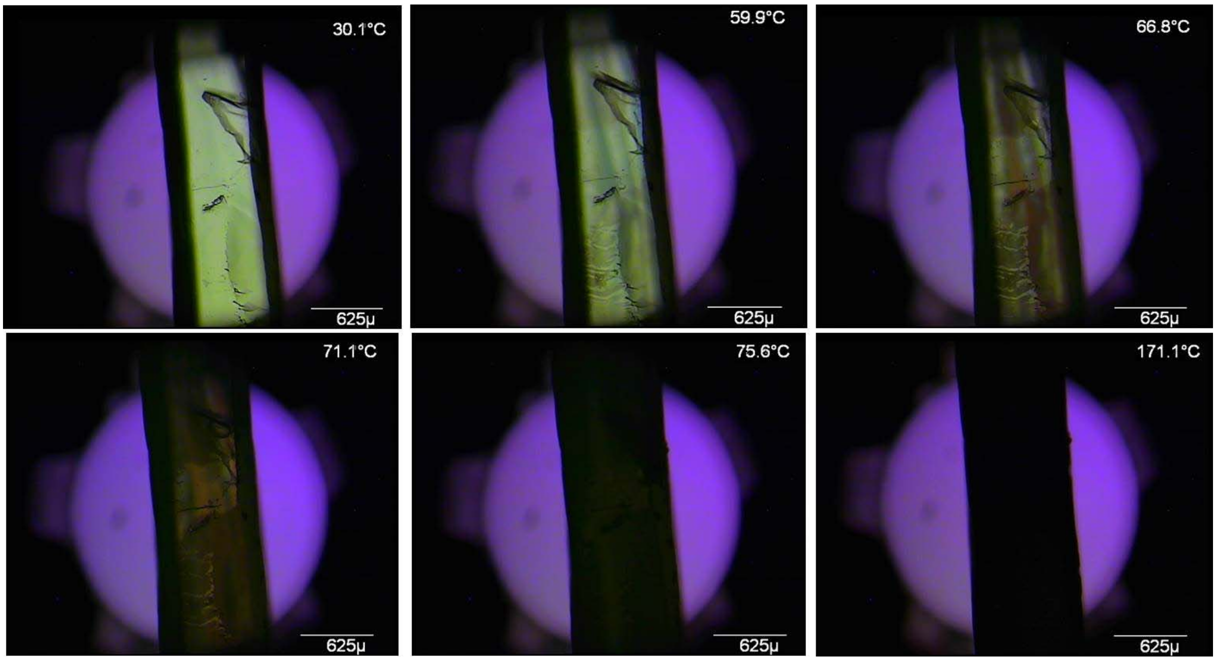

Figure S5 Optical micrographs of CBZ·ACE crystals at different temperatures under polarized light (heating rate of 10 ºC.min^{-1}).

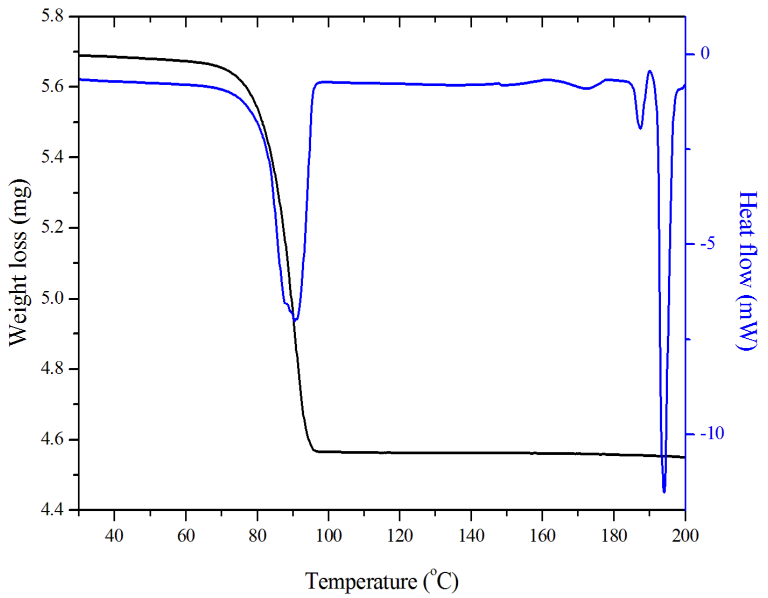

Figure S6 DSC and TGA thermal characterization of CBZ·ACE crystals (10 ºC.min^{-1}, perforated lid, N_2).

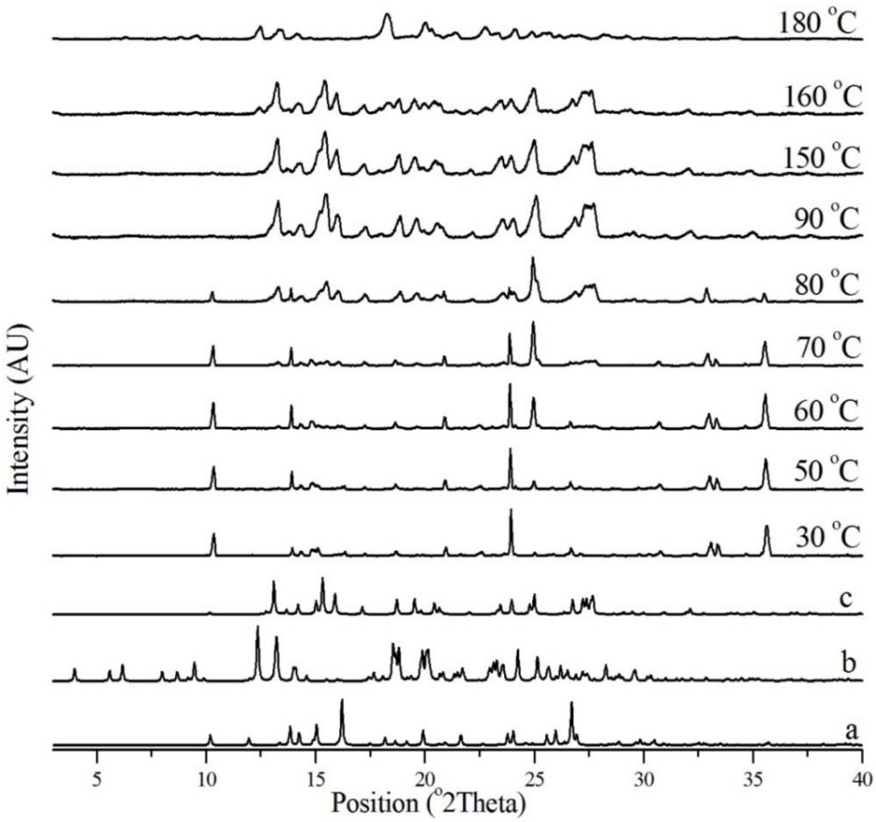

Figure S7 *In situ* variable temperature PXRD patterns of CBZ·ACE crystals (10 ºC.min^{-1}, 25-180 ºC). *a*: CBZ·ACE from CRBMZA01. *b*: CBZ Form I from CBMZPN11. *c*: CBZ Form III from CBMZPN01.

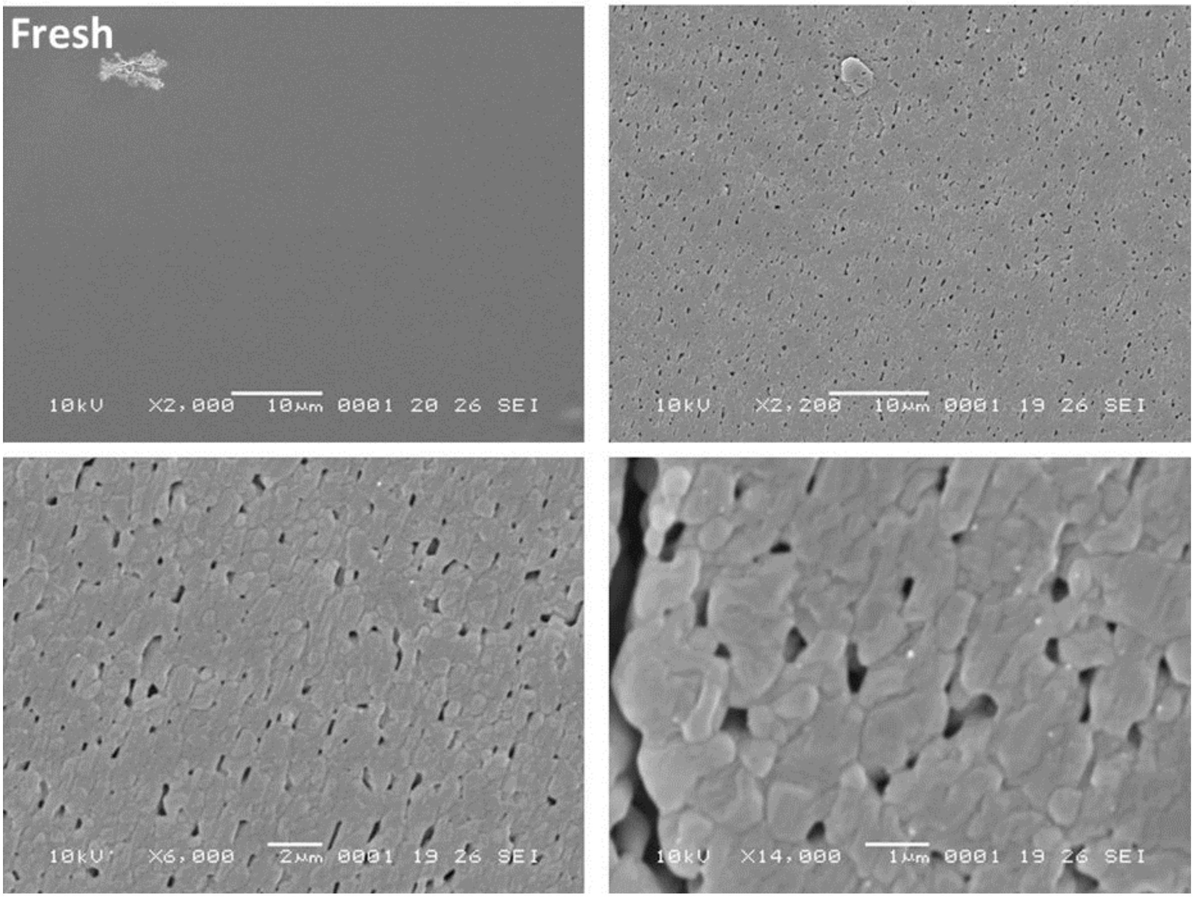

Figure S8 SEM micrographs of CBZ·ACE crystal surfaces heated until 120 ºC under N_2 (heating rate of 10 ºC.min^{-1}). The fresh surface is added for comparison purposes.

S3.2. CBZ N,N-dimethylsulfoxide solvate (CBZ·DMSO), Group 1

The weight loss associated with desolvation shows varying slopes throughout the event, which may suggest different physical transformations arising from the heating of the crystals. This phenomenon is also observed in the DSC curve as the characteristics of the endothermic event of desolvation change as the temperature increases: the event begins as a sharp endotherm (T_{onset}=100.8 ºC), but is followed by a broad event which lasts until about 170 ºC. At approximately 175 ºC, another endotherm indicates melting of Form III and recrystallization of Form I before it melts at about 190 ºC.

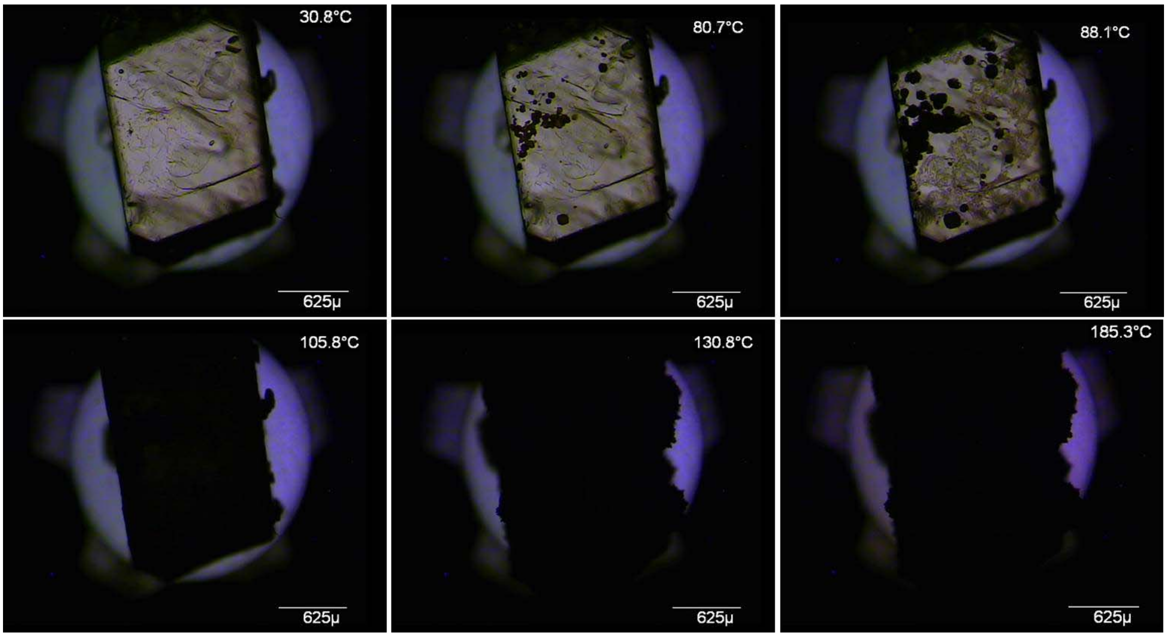

Figure S9 Optical micrographs of CBZ·DMSO crystals at different temperatures under polarized light (heating rate of 10 °C.min^{-1}).

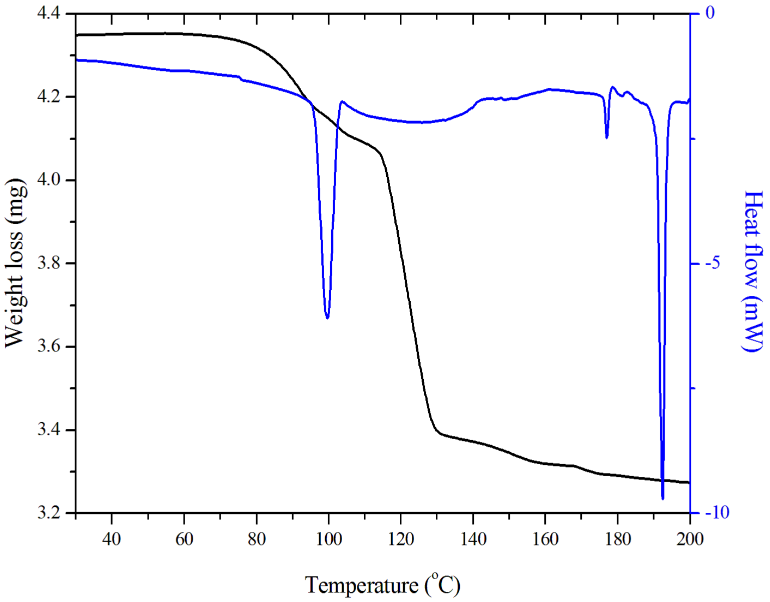

Figure S10DSC and TGA thermal characterization of CBZ·DMSO crystals (10 °C.min^{-1}, perforated lid, N_2).

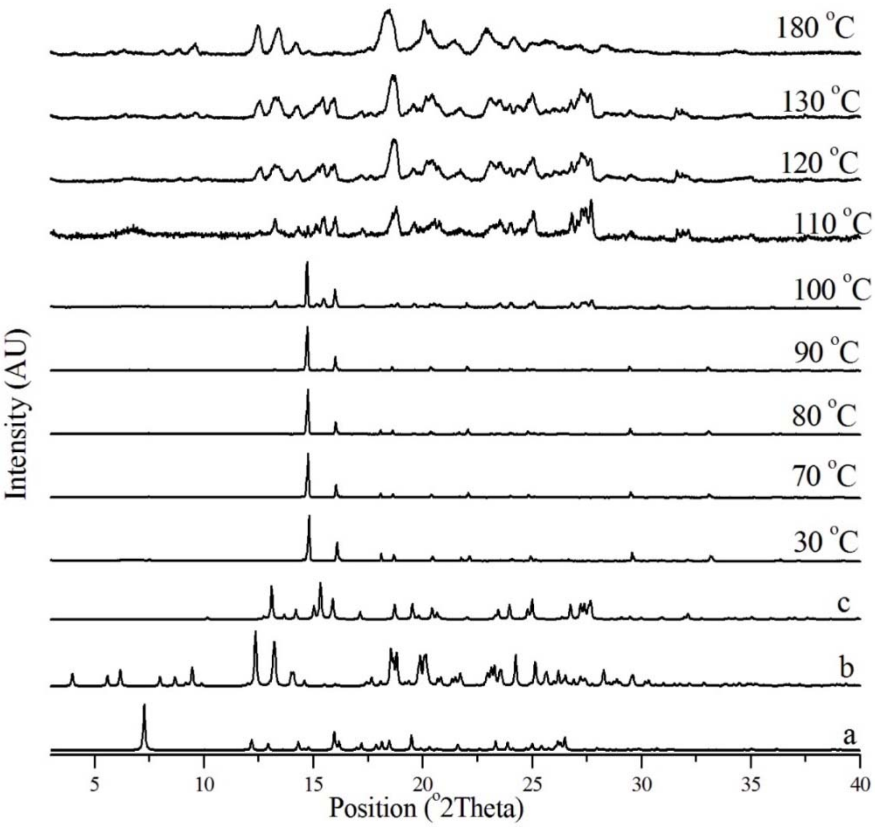

Figure S11*In situ* variable temperature PXRD patterns of CBZ·DMSO crystals (10 ºC.min^{-1}, 25-180 ºC). *a*: CBZ·DMSO from UNEYIV01. *b*: CBZ Form I from CBMZPN11. *c*: CBZ Form III from CBMZPN01.

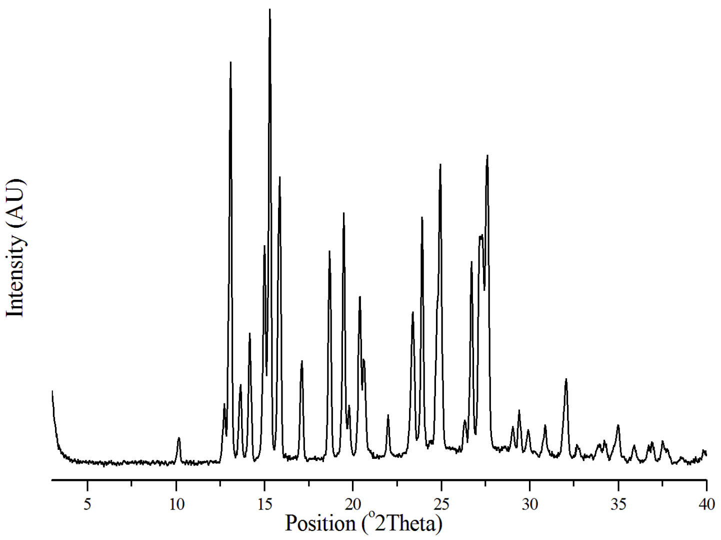

Figure S12*Ex situ* PXRD pattern of CBZ·DMSO crystals stored at 80 ºC for 5 h (N_2). The pattern corresponds to CBZ Form III.

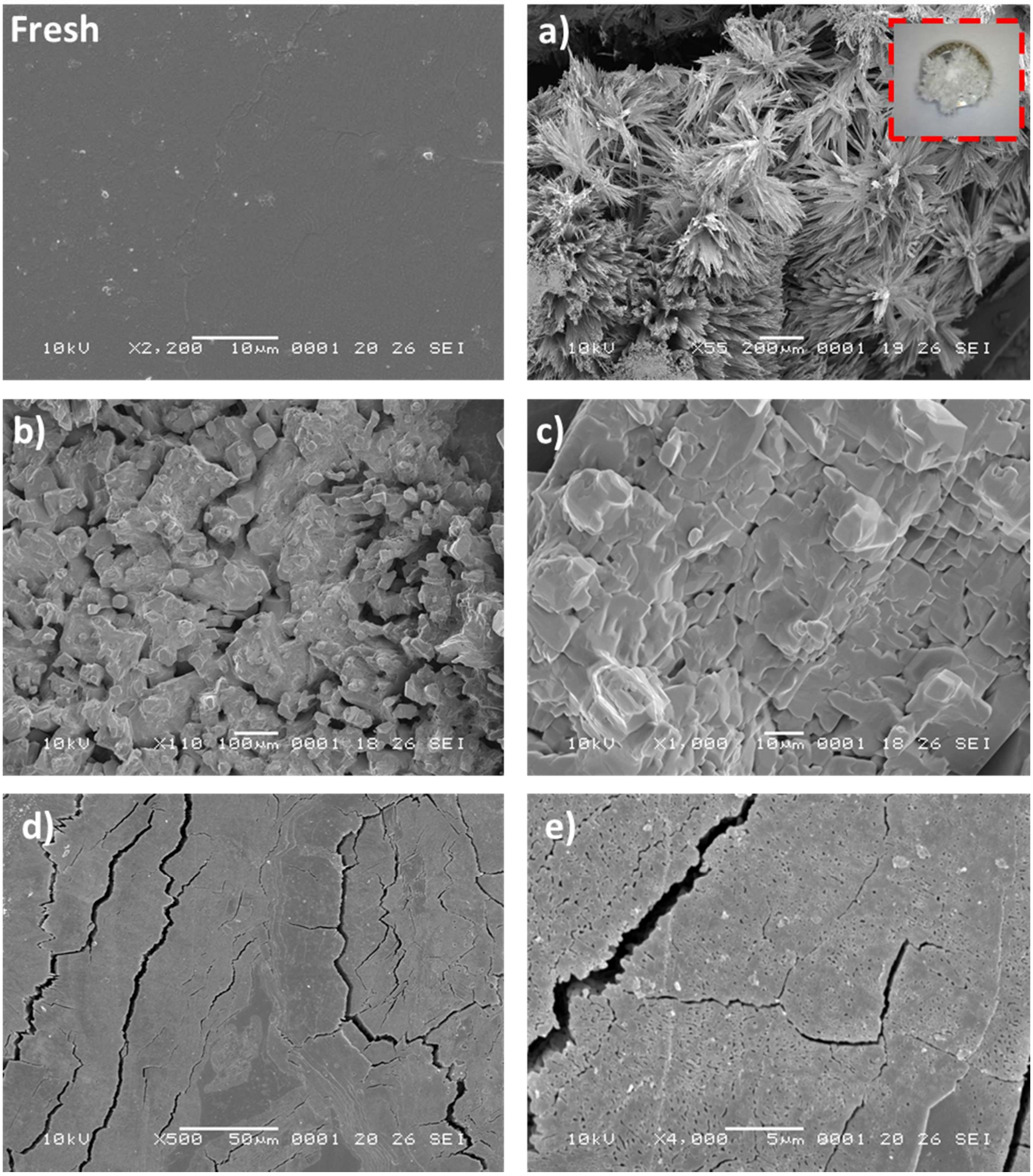

Figure S13SEM micrographs of CBZ·DMSO crystals subjected to desolvation (the fresh surface is added for comparison purposes). *a*: heated until 150 ºC (N_2, 10 ºC.min^{-1}); the detail shows a photograph of the sample after the experiment. *b* and *c*: heated until 110 ºC (N_2, heating rate of 10 ºC.min^{-1}). *d* and *e* : stored at 25 ºC (1 day, room conditions).

S3.3. CBZ N,N-dimethylformamide solvate (CBZ·DMF), Group 1

One event of weight loss ending at about 160 ºC with varying slope is linked to the desolvation of DMF. The heat flow curve shows that the endothermic event of desolvation changes in appearance

during the decomposition as it begins as a sharp event at approximately 80 °C, but is followed by a broad endotherm which lasts until just above 145 °C. This may suggest that concurrent physical transformations are taking place. Only the melting of CBZ Form I is seen afterwards (~ 190 °C).

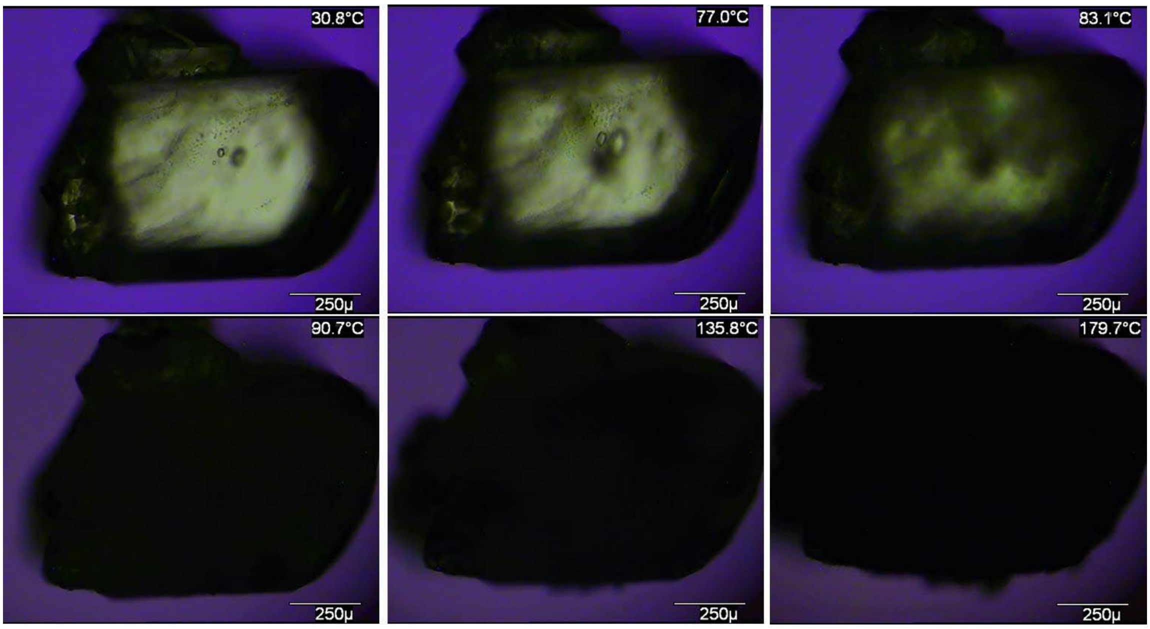

Figure S14 Optical micrographs of CBZ·DMF crystals at different temperatures under polarized light (heating rate of 10 °C.min^{-1}).

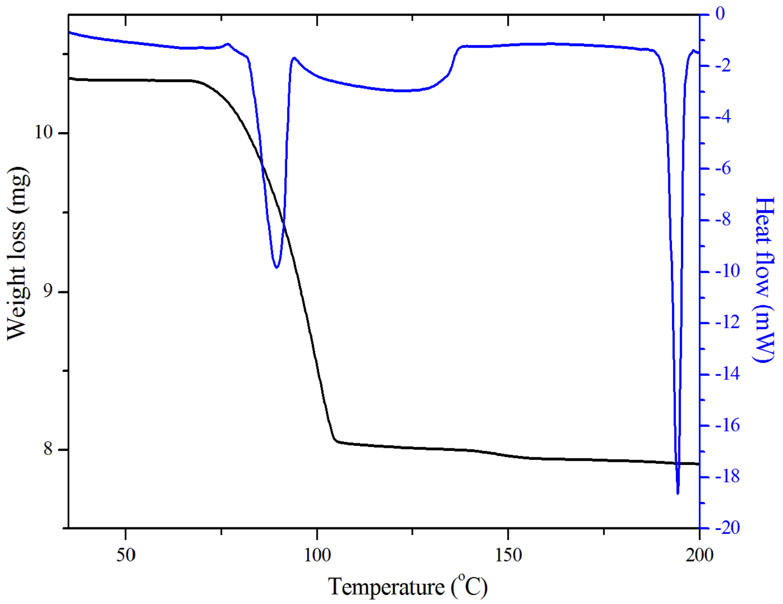

Figure S15DSC and TGA thermal characterization of CBZ·DMF crystals (10 °C.min^{-1}, perforated lid, N_2).

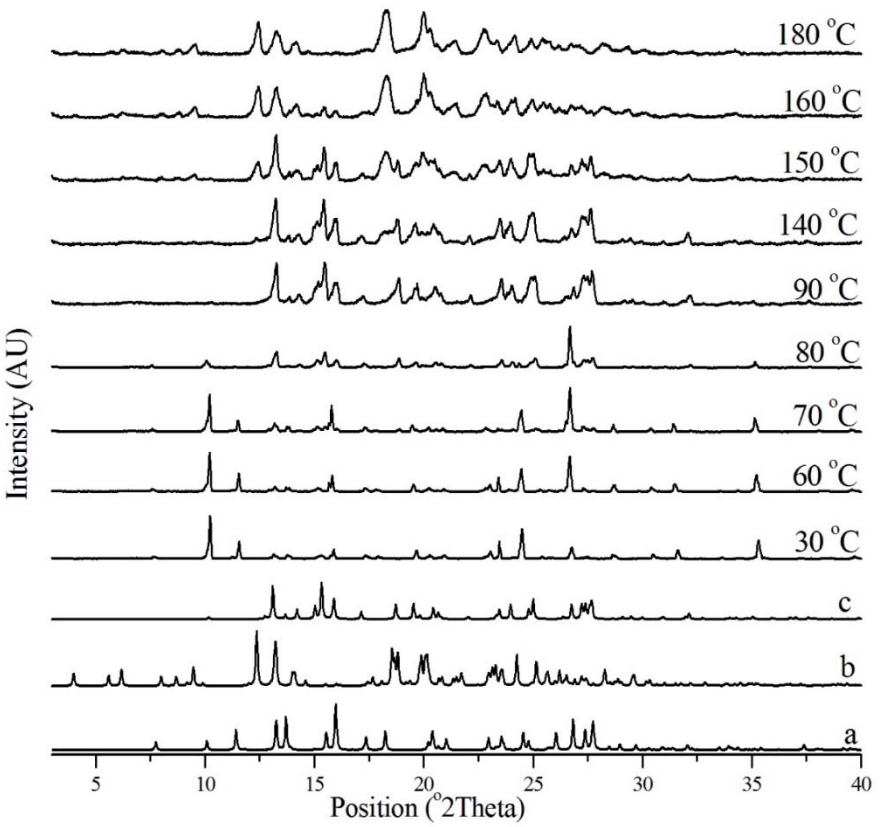

Figure S16 *In situ* variable temperature PXRD patterns of CBZ·DMF crystals (10 ºC.min^{-1}, 25-180 ºC). *a*: CBZ·DMF from QANQUS. *b*: CBZ Form I from CBMZPN11. *c*: CBZ Form III from CBMZPN01.

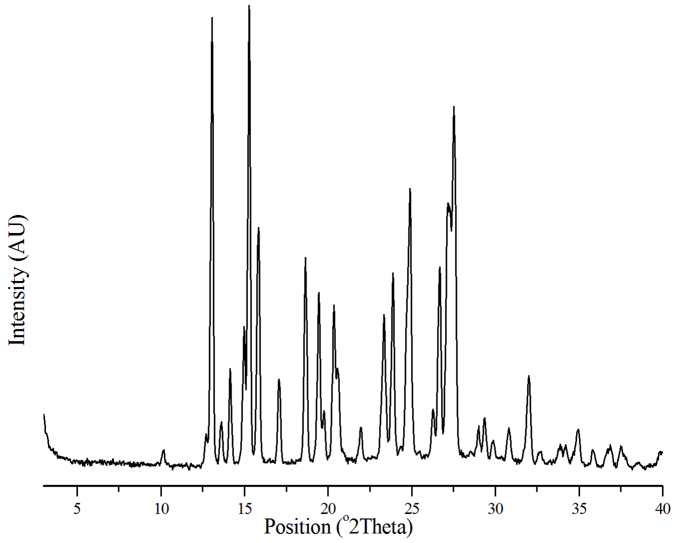

Figure S17 *Ex situ* PXRD pattern of CBZ·DMF crystals stored at 50 ºC for 2 hours (N_2). The pattern corresponds to CBZ Form III.

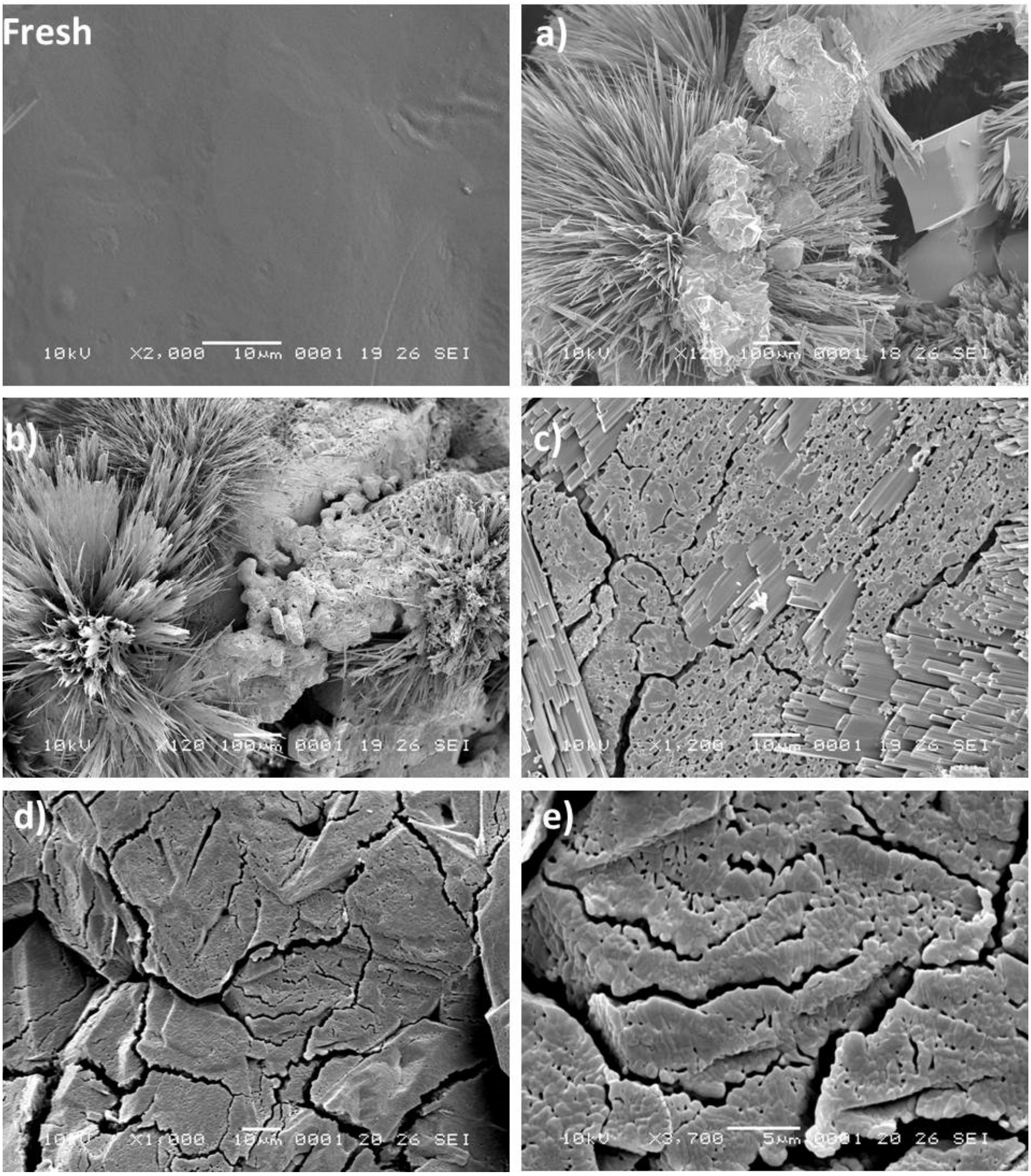

Figure S18SEM micrographs of CBZ·DMF crystal surfaces subjected to desolvation (the fresh surface is added for comparison purposes). *a* and *b*: heated until 150 ºC (N_2, 10 ºC.min^{-1}); the detail shows a photograph of the sample after the experiment. *c*: heated until 110 ºC (N_2, heating rate of 10 ºC.min^{-1}). *d* and *e* : stored at 25 ºC (1 day, room conditions).

S3.4. CBZ N,N-dimethylacetamide solvate (CBZ·DMA), Group 2

A single event of weight loss (ending at about 90 ºC) is observed in the TGA, while the heat flow curve shows that desolvation is characterized by a single endothermic event (T_{onset}=76.6 ºC). Although

the desolvation endotherm is not symmetric, the shape of the thermograms and the rate of weight loss are different to those observed for CBZ·DMSO and CBZ·DMF. The melting of the desolvate consists of a broad endotherm that may consist of two events. It is unclear whether this behaviour is related to differences in particle size or if it is correlated to the melting of polymorphs III and I.

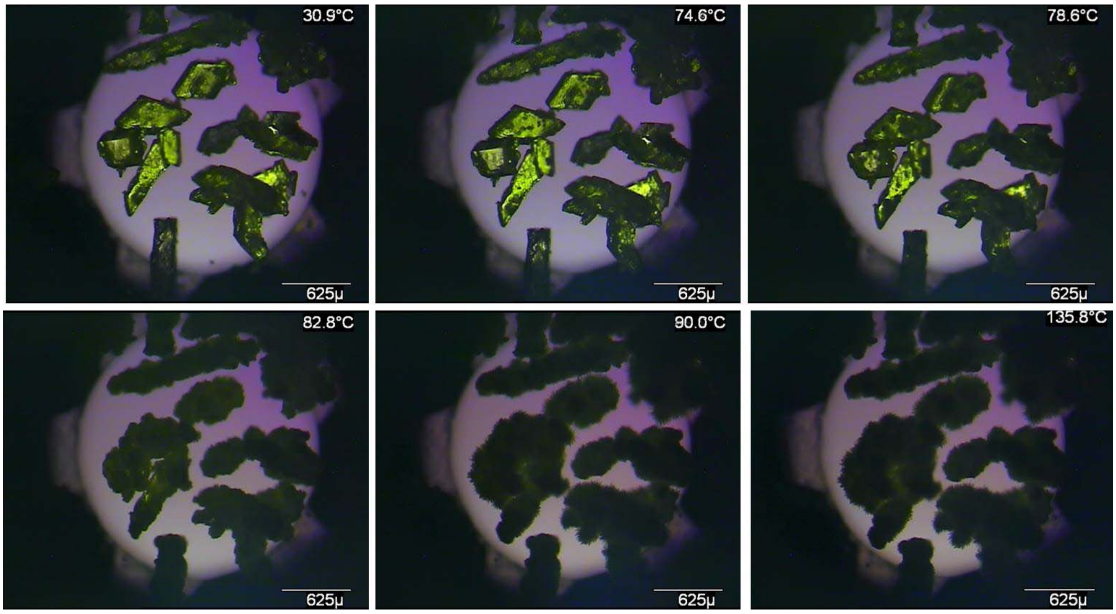

Figure S19 Optical micrographs of CBZ·DMA crystals at different temperatures under polarized light (heating rate of 10 °C.min^{-1}).

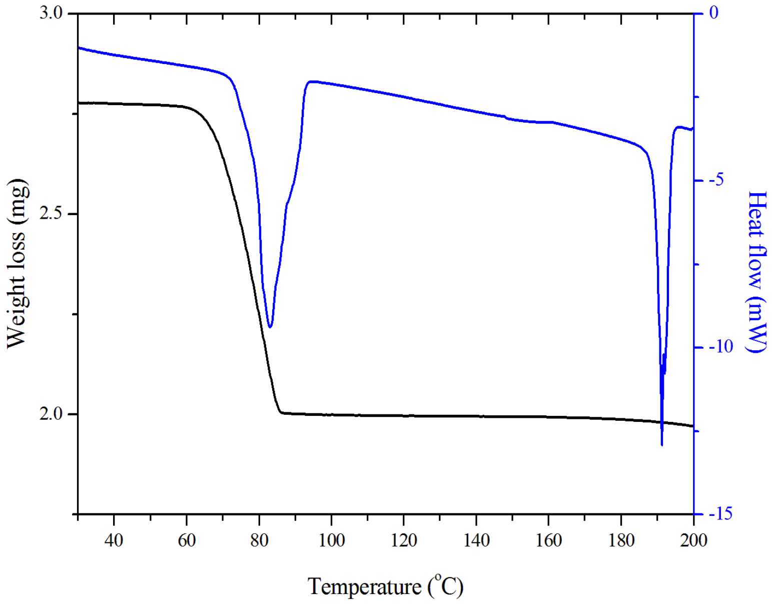

Figure S20DSC and TGA thermal characterization of CBZ·DMA crystals (10 °C.min^{-1}, perforated lid, N_2).

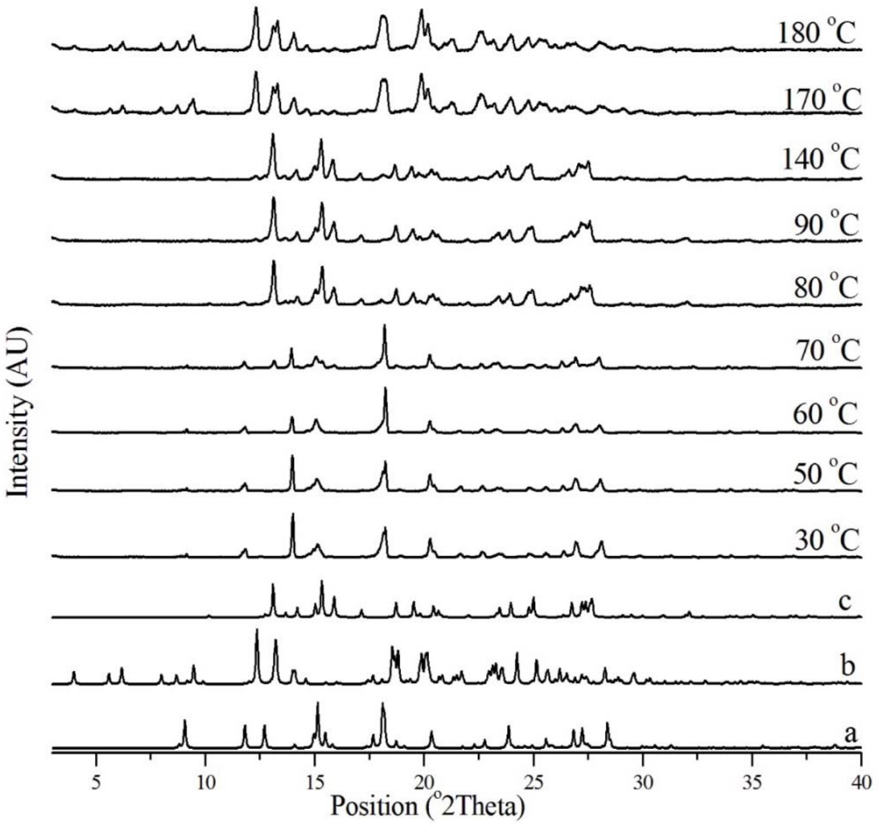

Figure S21 *In situ* variable temperature PXRD patterns of CBZ·DMA crystals (10 ºC.min^{-1}, 25-180 ºC). *a*: CBZ·DMA from KIWBEY. *b*: CBZ Form I from CBMZPN11. *c*: CBZ Form III from CBMZPN01.

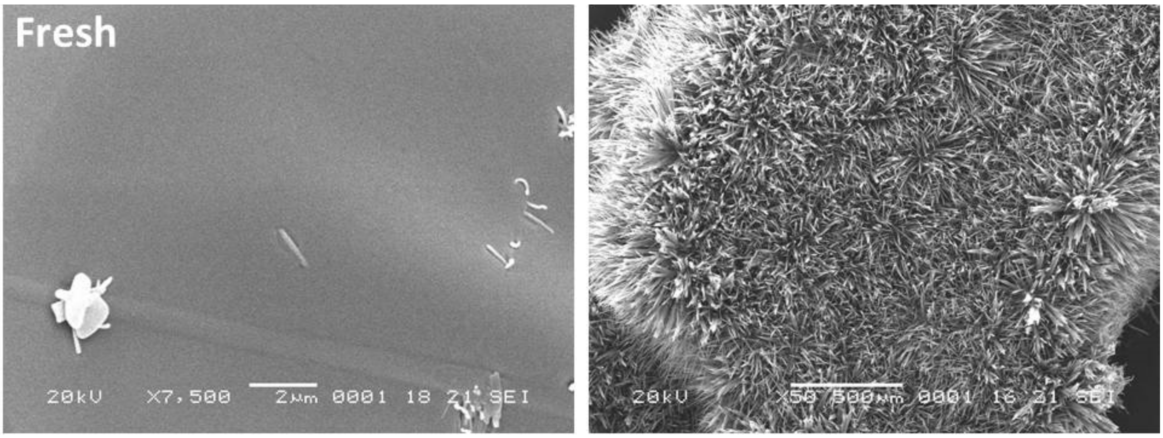

Figure S22 SEM micrograph of CBZ·DMA crystals heated until 120 ºC (N_2, heating rate of 10 ºC.min^{-1}). The fresh surface is added for comparison purposes

S3.5. CBZ 1,4-benzoquinone cocrystals (CBZ·BZQ and 2CBZ·BZQ), Group 2 and Group 3

In the case of CBZ·BZQ, a single event of weight loss ending at about 160 ºC is observed by TGA, and the heat flow curve demonstrates that desolvation is characterized by a broad endothermic event (T_{onset}=146.2 ºC). For comparison purposes, the sublimation of BZQ crystals is illustrated in **Figure S22**. The TGA of BZQ shows a single weight loss between 60 to 160 ºC, while the DSC curve displays two endotherms. These findings agree with the literature, which suggests that benzoquinone concomitantly melts and sublimes (Sousa *et al.*, 2012).

The DSC and TGA curves of the 2:1 cocrystal are very similar to the 1:1 cocrystal, while the onset temperature of sublimation appears to be slightly higher (T_{onset}=155.1 ºC).

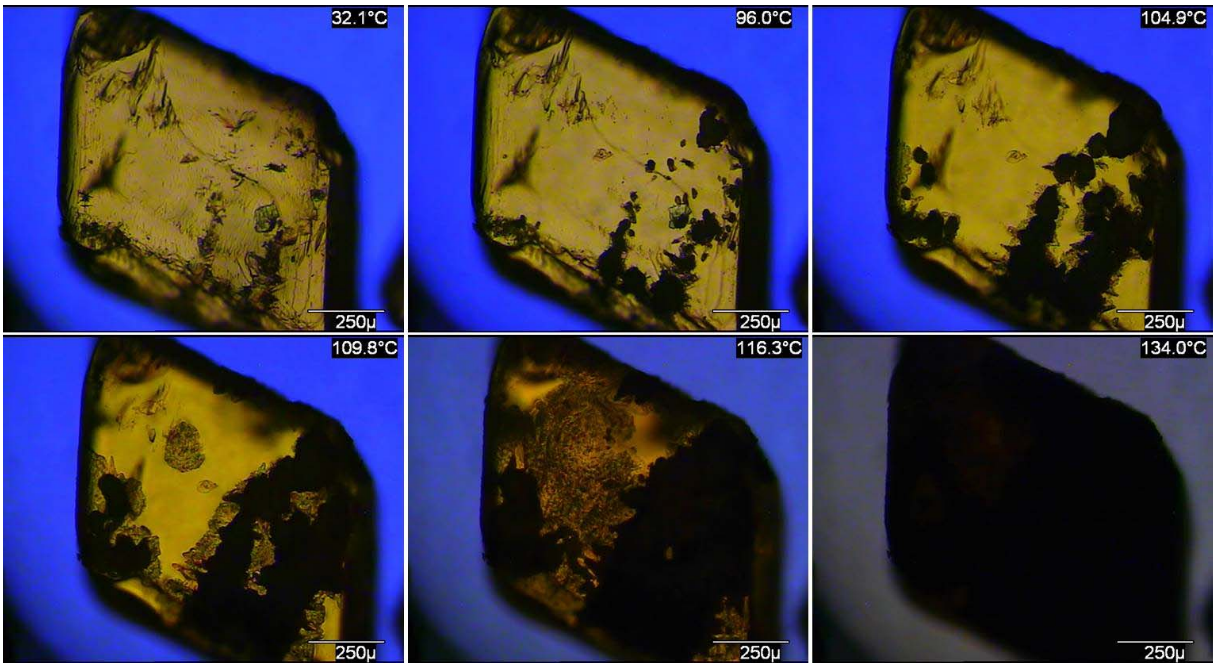

Figure S23 Optical micrographs of CBZ·BZQ crystals at different temperatures under polarized light (heating rate of 10 ºC.min^{-1}).

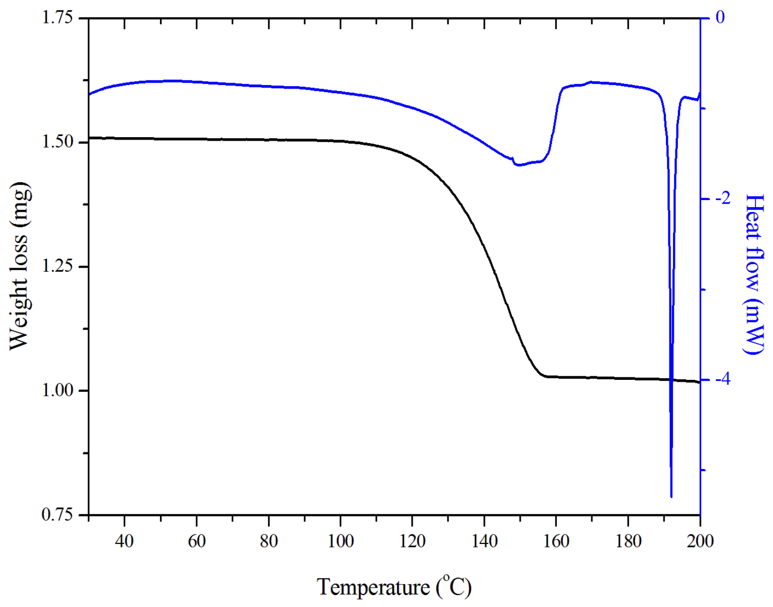

Figure S24DSC and TGA thermal characterization of CBZ·BZQ crystals (10 ºC.min^{-1}, perforated lid, N_2).

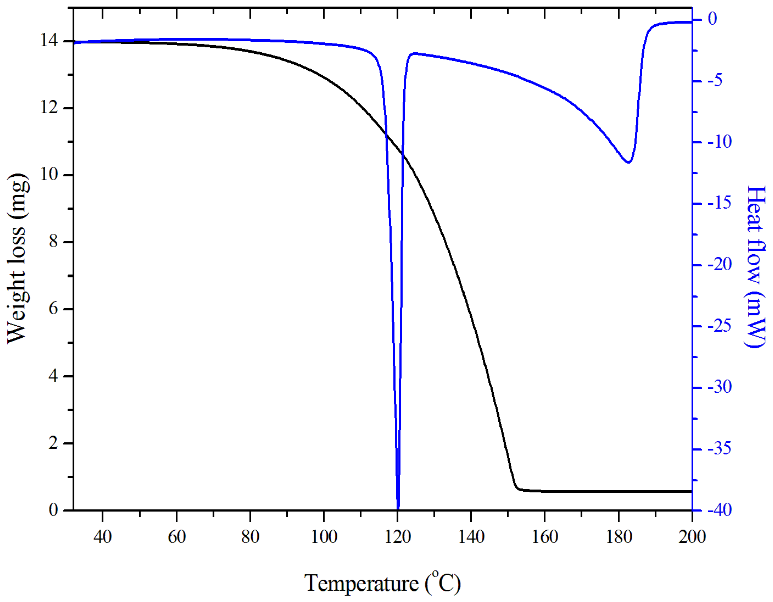

Figure S25DSC and TGA thermal characterization of BZQ crystals (10 ºC.min^{-1}, perforated lid, N_2).

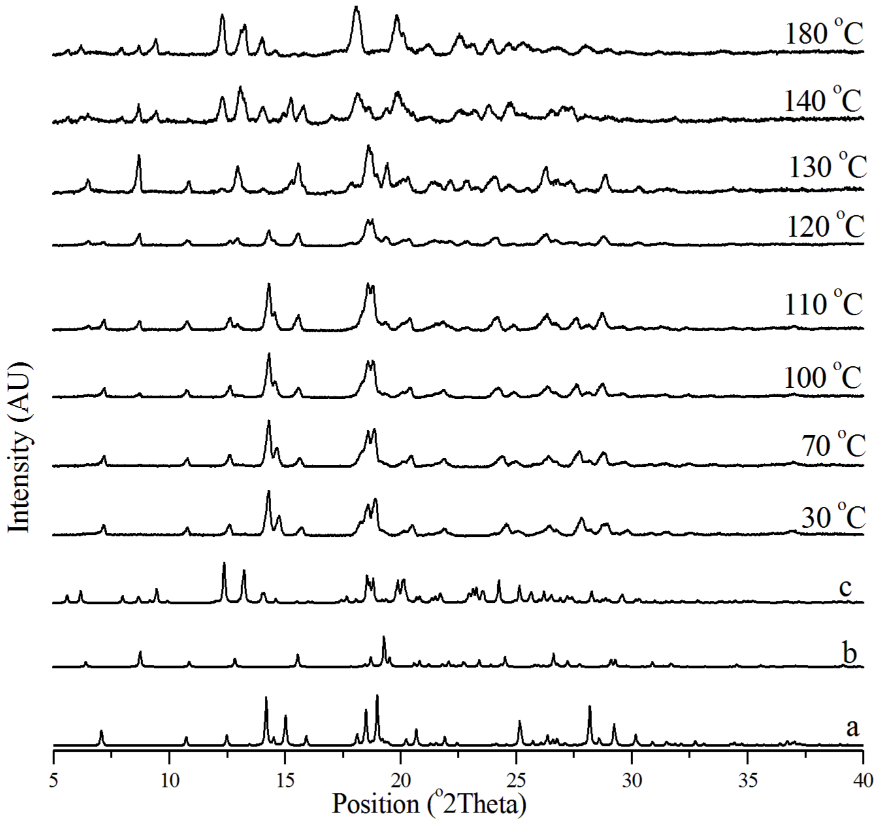

Figure S26*In situ* variable temperature PXRD patterns of CBZ·BZQ crystals (10 ºC.min^{-1}, 25-180 ºC). *a*: CBZ·BZQ from in-house data. *b*: CBZ Form I from CBMZPN11. *c*: CBZ Form III from CBMZPN01.

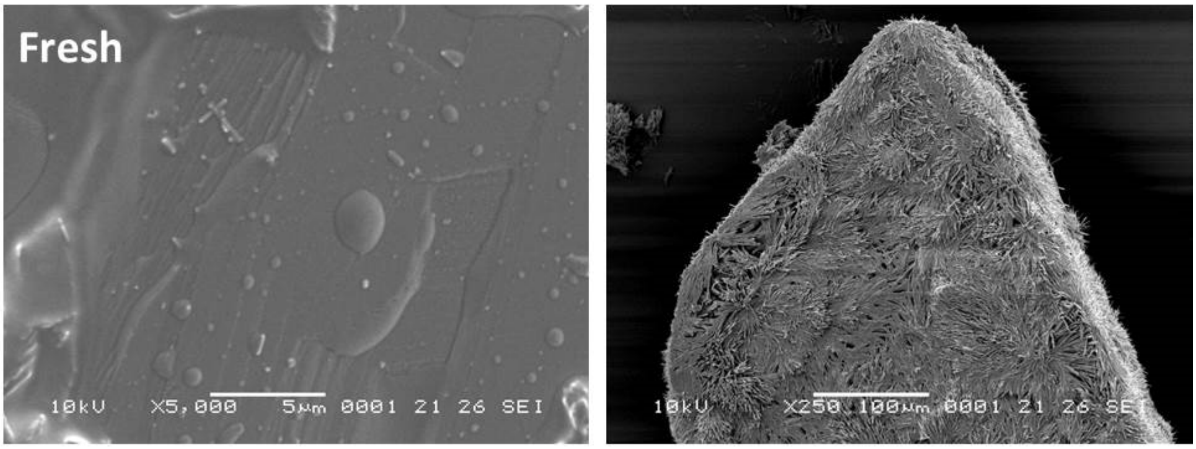

Figure S27SEM micrographs of CBZ·BZQ crystals heated until 150 ºC (N_2, heating rate of 10 ºC.min^{-1}). The fresh surface is added for comparison purposes.

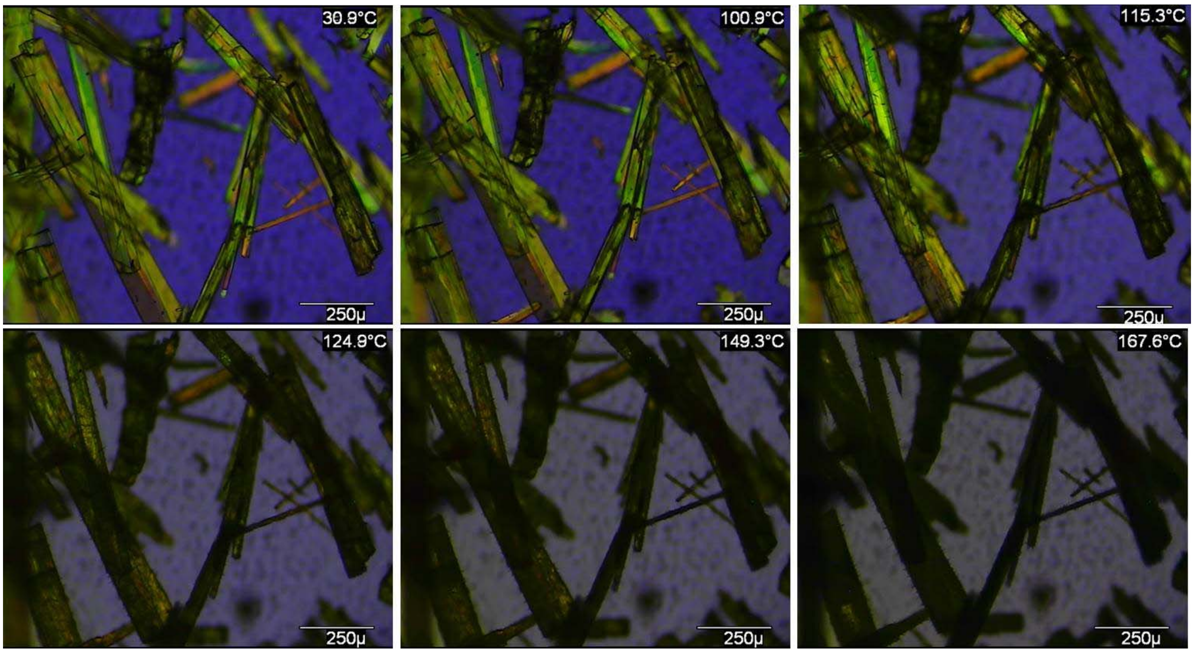

Figure S28 Optical micrographs of 2CBZ·BZQ crystals at different temperatures under polarized light (heating rate of 10 °C.min^{-1}).

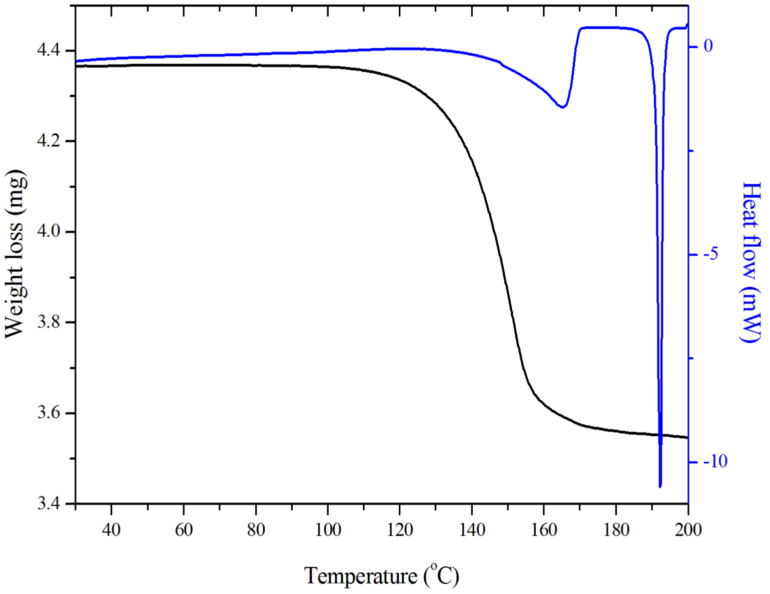

Figure S29DSC and TGA thermal characterization of 2CBZ·BZQ crystals (10 °C.min^{-1}, perforated lid, N_2).

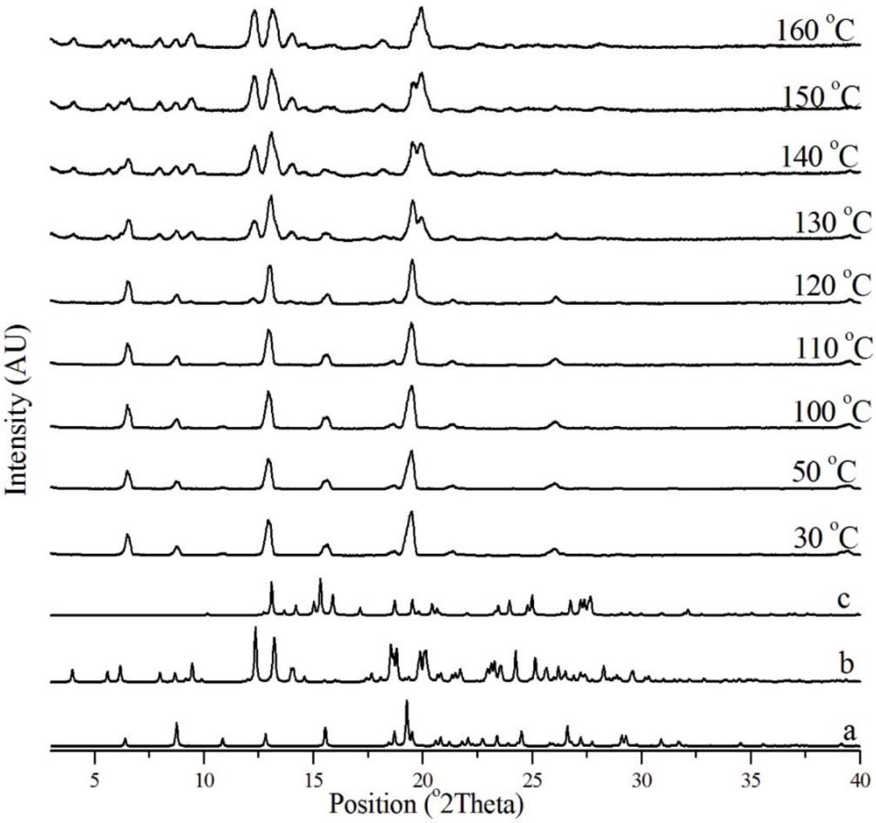

Figure S30 *In situ* variable temperature PXRD patterns of 2CBZ·BZQ crystals (10 ºC.min^{-1}, 25-180 ºC). *a*: 2CBZ·BZQ from UNEYOB. *b*: CBZ Form I from CBMZPN11. *c*: CBZ Form III from CBMZPN01.

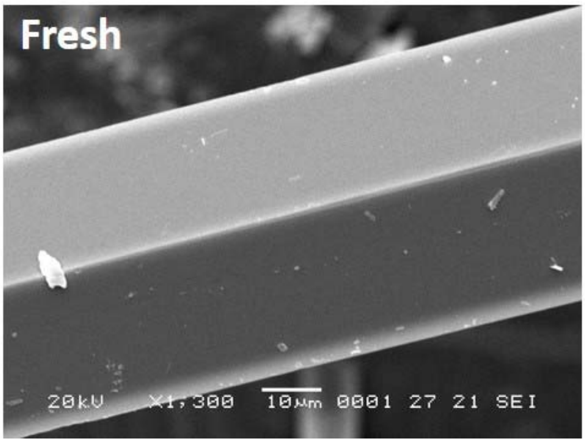

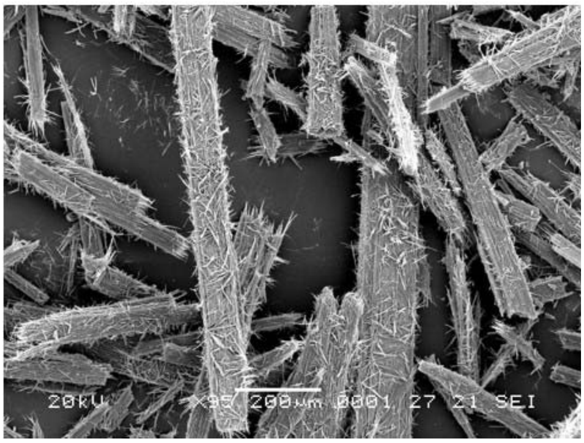

Figure S31 SEM micrographs of 2CBZ·BZQ crystals heated until 150 ºC (N_2, heating rate of 10 ºC.min^{-1}). The fresh surface is added for comparison purposes.

S3.6. CBZ oxalic acid cocrystal (2CBZ·OXA), Group 3

The DSC curves of 2CBZ·OXA collected at 1 and 10 ºC.min^{-1} show one sharp endothermic event in the range 150-175 ºC, which is followed by broad endotherms at higher temperature. Although the general results were reproducible, the experiments performed at slow heating rate identify additional exothermic events during heating which may be masked by the other events at higher rates.

The TGA curve demonstrates three regions of weight loss without clear limits: (i) from approximately 140 to 160 ºC; (ii) from approximately 160 to 260 ºC; and (iii) above 160 ºC. The shape and the slopes of regions (ii) and (iii) are equivalent to the characteristics of the decomposition of CBZ (see **Figure S63** and **Figure S64**). In the case of the OXA cocrystal, however, these events are shifted to lower temperatures. The results may demonstrate that the physical decomposition of the cocrystal accelerates the chemical decomposition of CBZ without a change in mechanism.

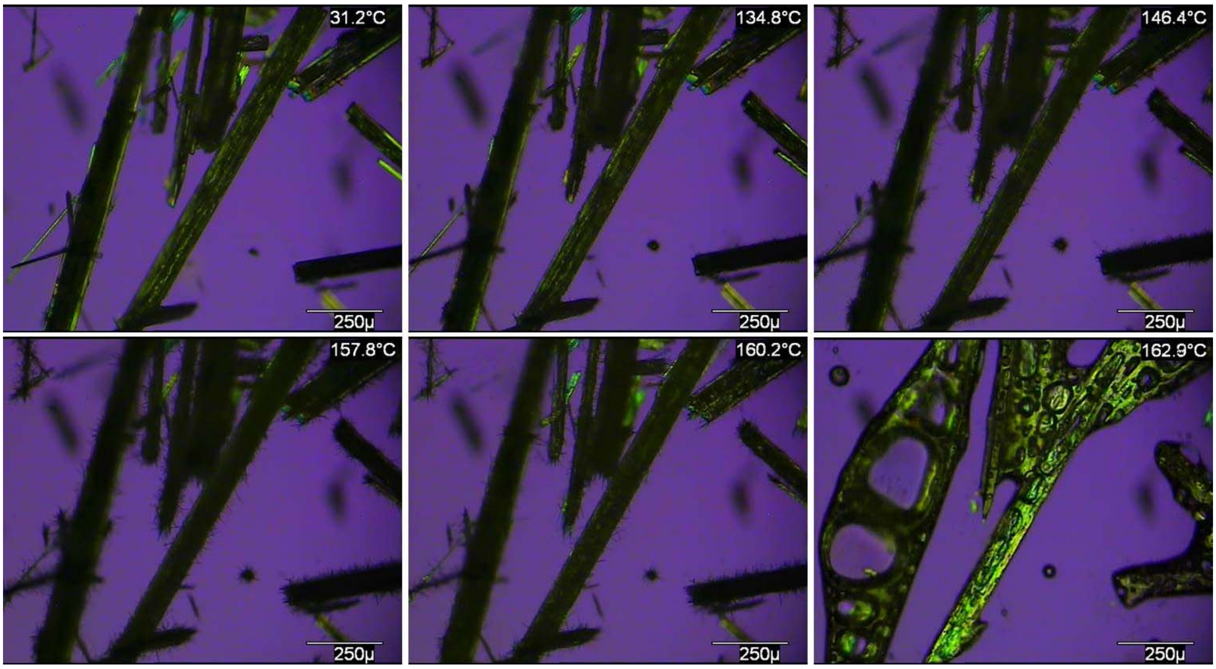

Figure S32 Optical micrographs of 2CBZ·OXA crystals at different temperatures under polarized light (heating rate of 10 ºC.min^{-1}).

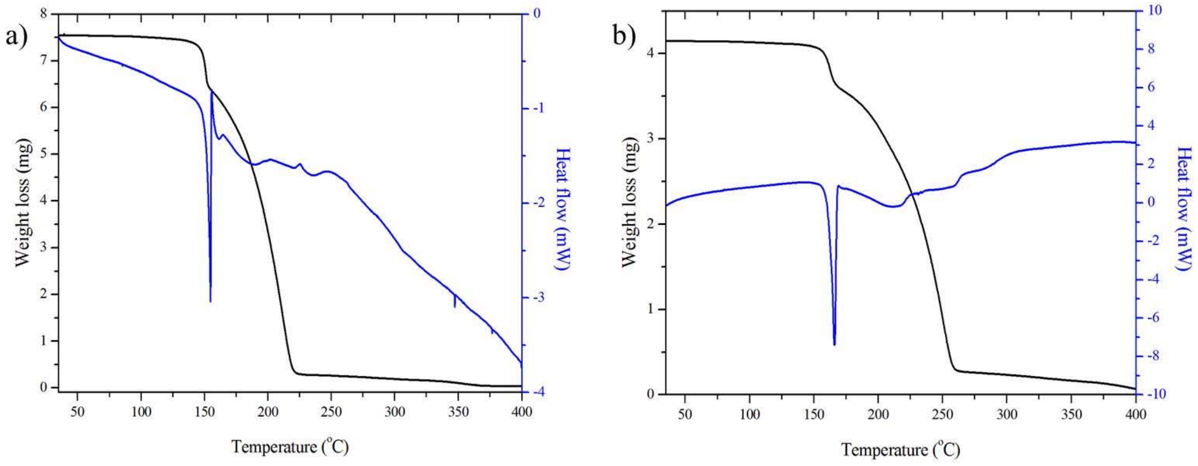

Figure S33DSC and TGA thermal characterisation of 2CBZ·OXA. *a:* 1 ºC.min^{-1}, perforated lid, N_2. *b*: 10 ºC.min^{-1}, perforated lid, N_2.

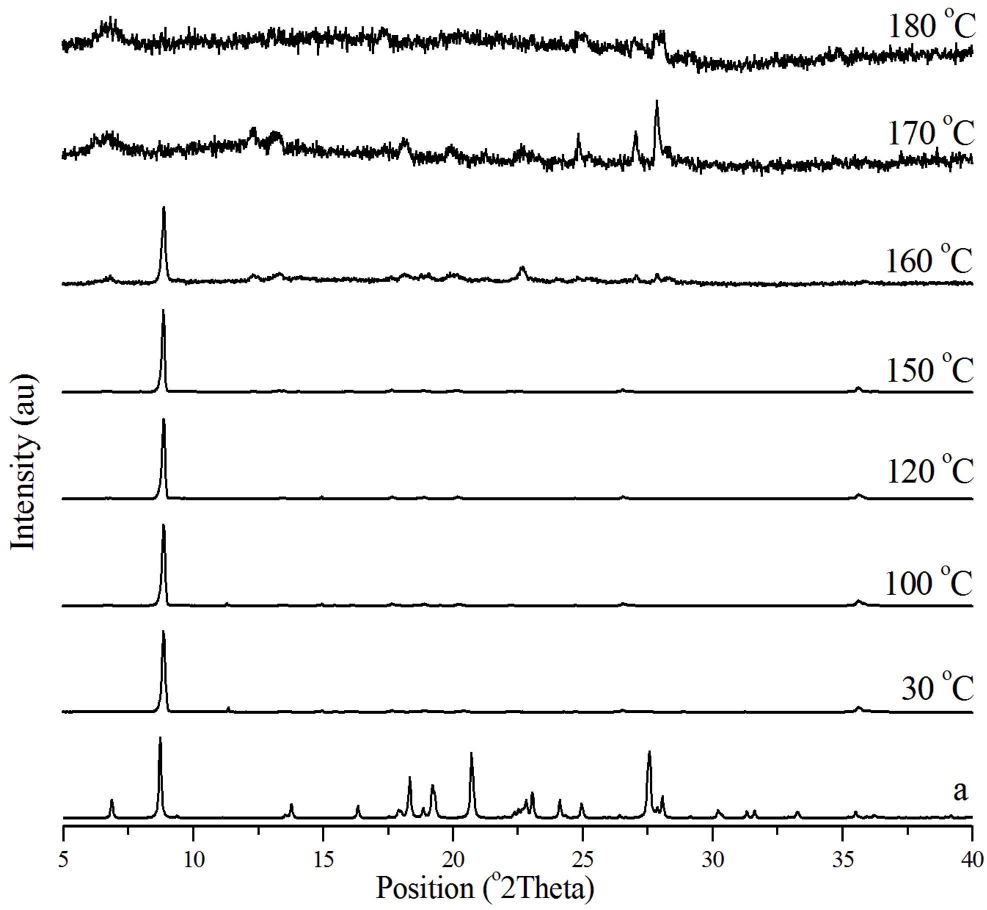

Figure S34*In situ* variable temperature PXRD patterns of 2CBZ·OXA crystals (10 ºC.min^{-1}, 25-180 ºC). *a*: 2CBZ·OXA from in-house data.

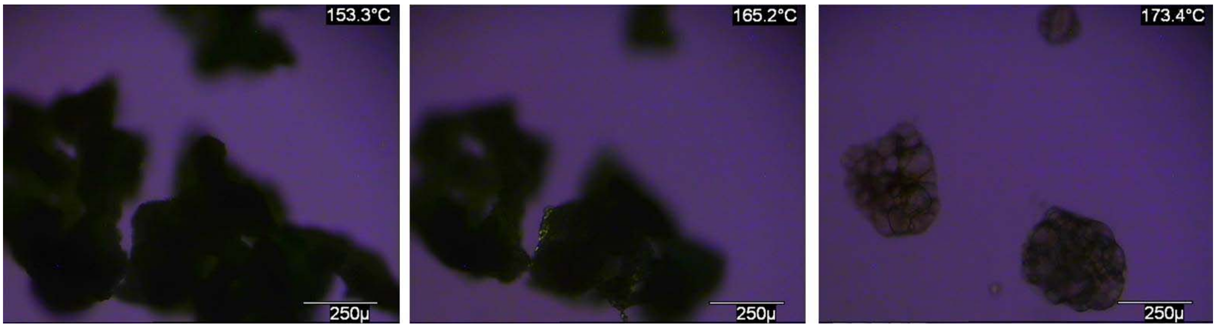

Figure S35 Optical micrographs of OXA crystals at different temperatures under polarized light (heating rate of 10 °C.min^{-1}).

S3.7. CBZ dihydrate (CBZ·2H_2O), Group 3

CBZ·2H_2O (1) and (2) are samples that differ on their morphology (*i.e.* they present different dominant crystal surfaces and particle size). They were both dehydrated to investigate whether such morphology variation could affect the polymorphic outcome after thermal decomposition. The thermal analyses have shown that while sample 1 shows one single weight loss ending at about 65 °C, crystals of sample 2 show a TGA curve with different slopes, resembling a two-step process finishing at approximately 90 °C. The same characteristic is observed in the DSC experiments where sample 1 shows one endothermic event followed by an exothermic event above 70 °C. CBZ·2H_2O sample 2, in turn, displays two non-resolved endothermic events at higher temperatures and no visible exothermic event.

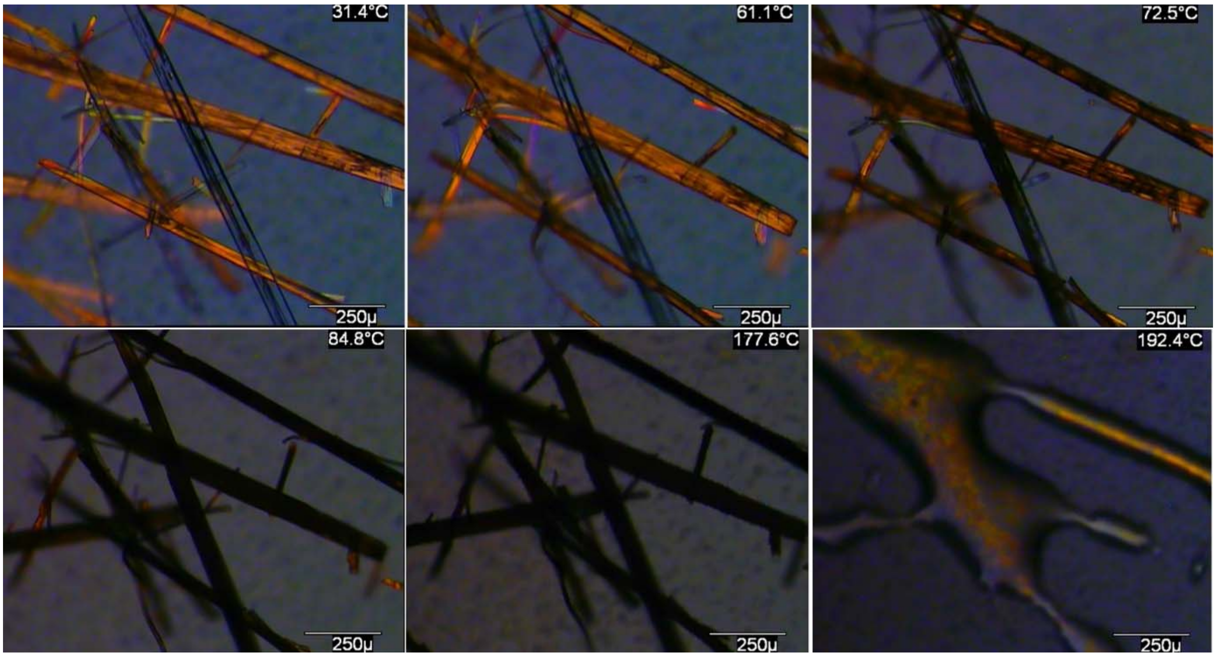

Figure S36 Optical micrographs of CBZ·$2H_2O$ sample (1) at different temperatures under polarized light (heating rate of 10 ºC.min^{-1}).

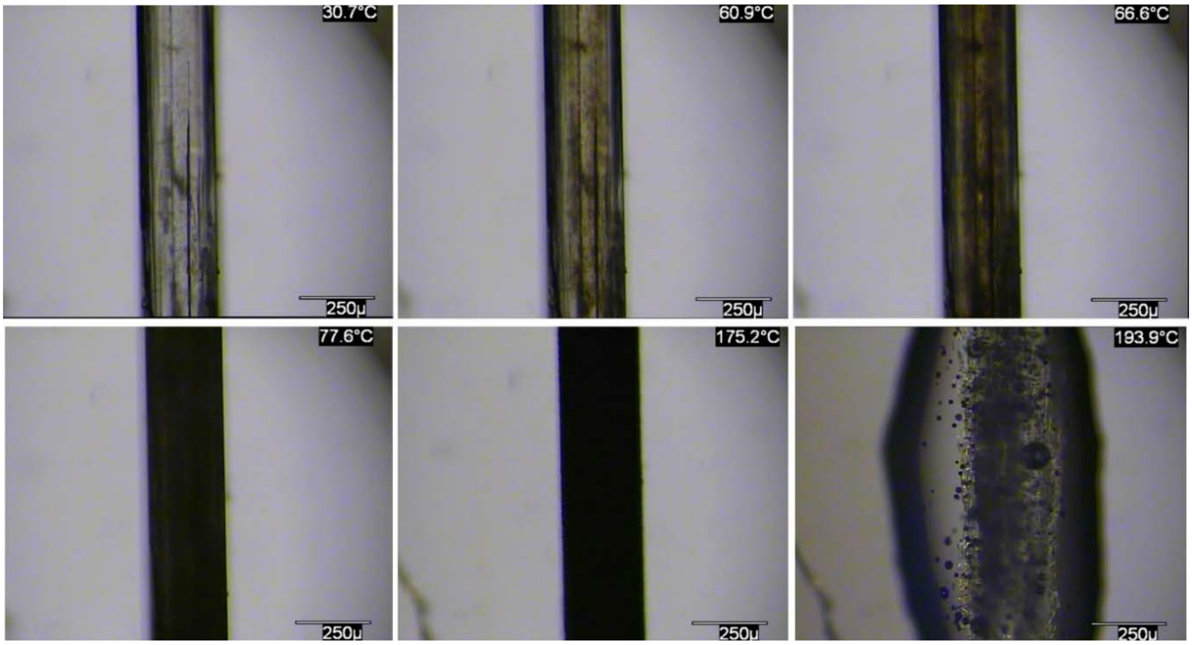

Figure S37 Optical micrographs of CBZ·$2H_2O$ sample (2) at different temperatures under polarized light (heating rate of 10 ºC.min^{-1}).

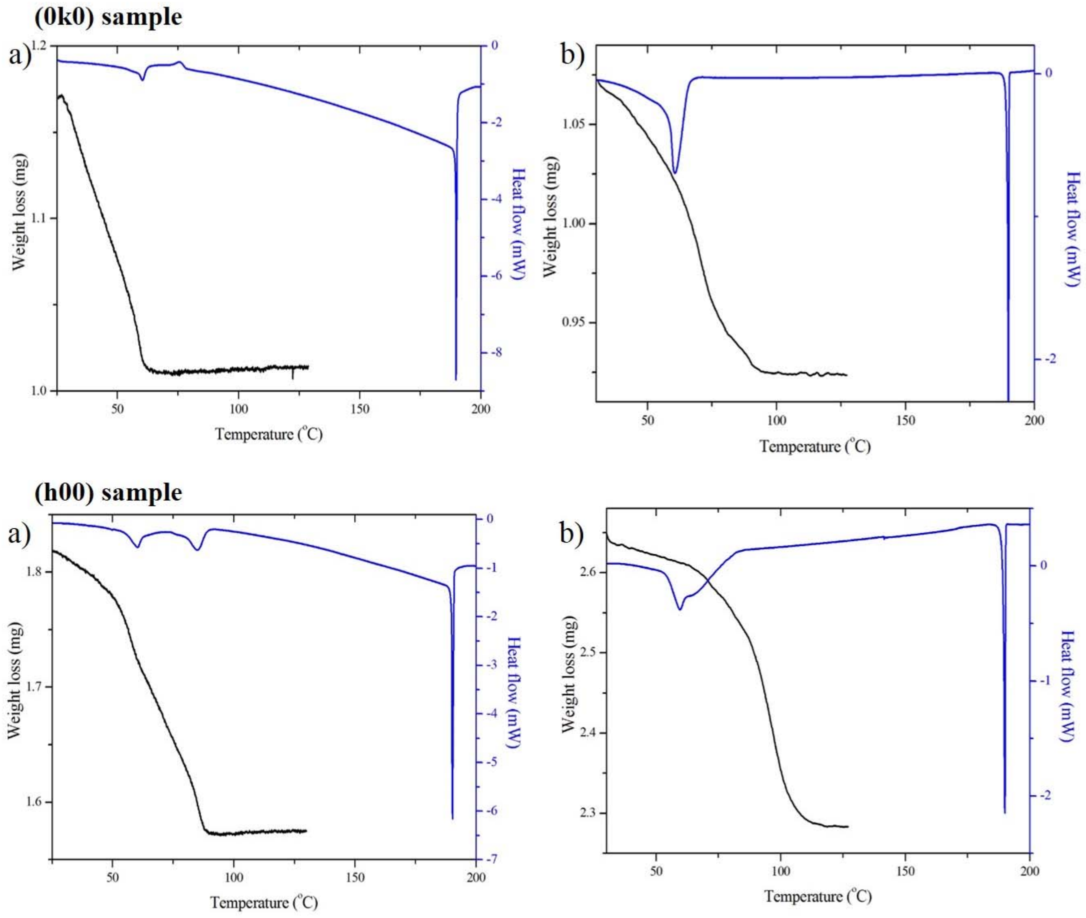

Figure S38DSC and TGA thermal characterization of CBZ·2H_2O samples 1/{0*k*0} and 2/{*h*00}. *a:* open pan, 1 ºC.min^{-1}, N_2. *b:* perforated lid, 10 ºC.min^{-1}, N_2.

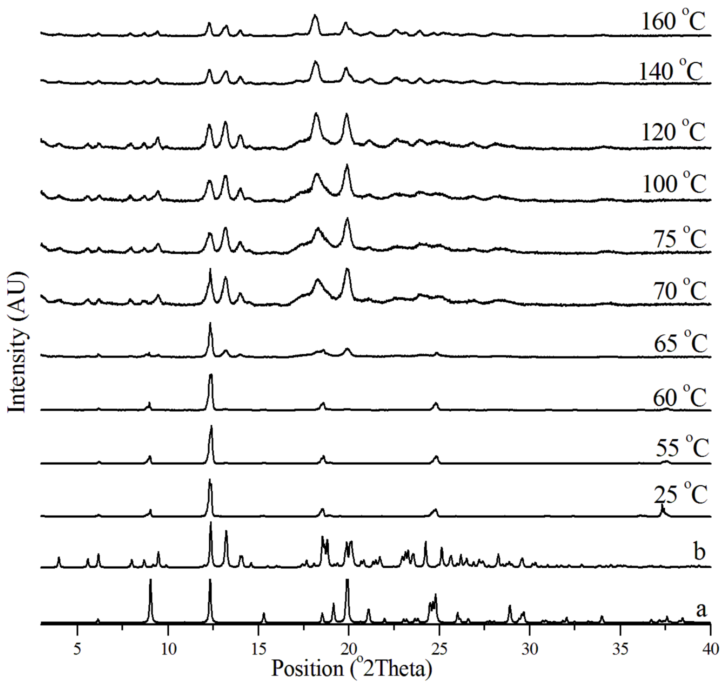

Figure S39*In situ* variable temperature PXRD patterns of CBZ·2H_2O sample (1) (1 ºC.min^{-1}, 25-160 ºC). *a*: CBZ·2H_2O from FEFNOT. *b*: CBZ Form I from CBMZPN11.

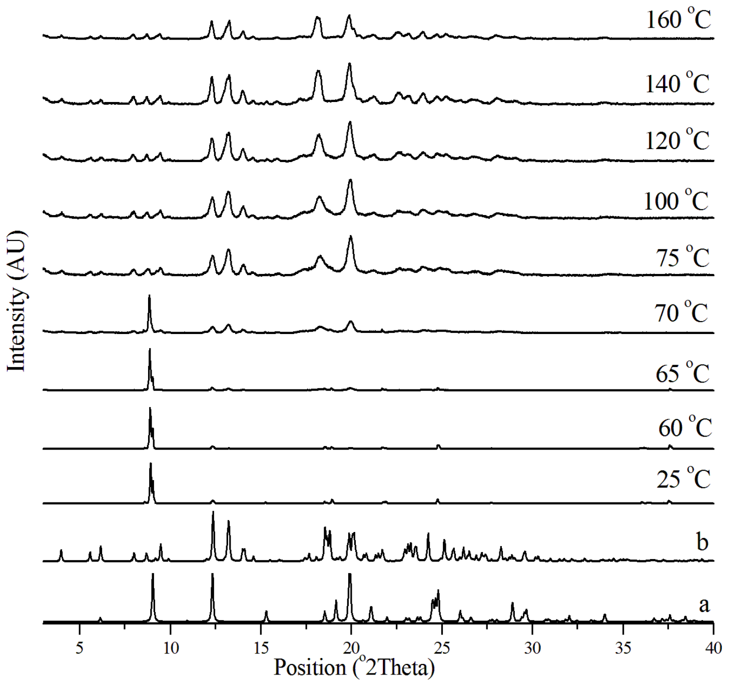

Figure S40*In situ* variable temperature PXRD patterns of CBZ·2H_2O sample (2) (1 ºC.min^{-1}, 25-160 ºC). *a*: CBZ·2H_2O from FEFNOT. *b*: CBZ Form I from CBMZPN11.

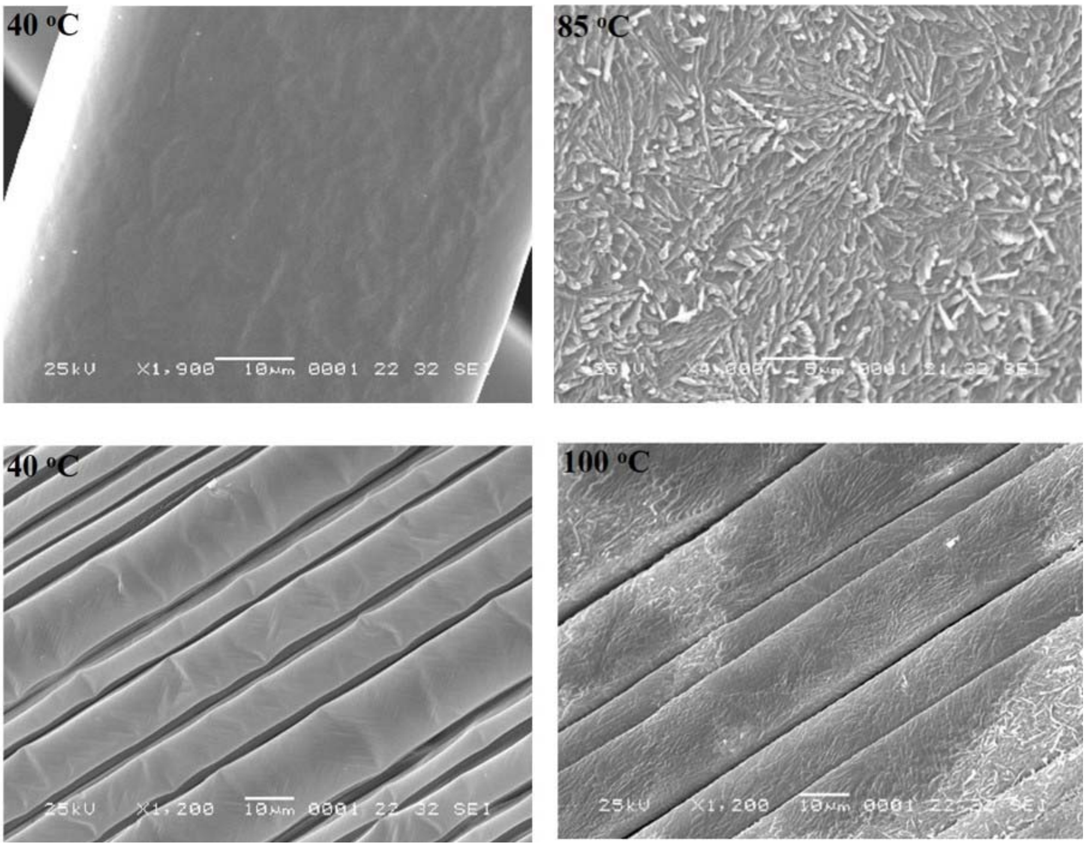

Figure S41 SEM micrographs of heated CBZ·2H_2O crystals (N_2, heating rate of 1 ºC.min^{-1}). Top: sample (1) {0*k*0} dominant surfaces; bottom: sample (2) {*h*00} dominant surfaces.

S3.8. CBZ formamide solvate (CBZ·FORM), Group 3

A single weight loss event is observed by TGA in the range 80-180 ºC. The heat flow curve shows desolvation as a sharp endotherm close to 147 ºC. The shape of the curve, however, suggests that the sample starts absorbing energy at a different rate at much lower temperatures. Exothermic events are seen above 150 ºC.

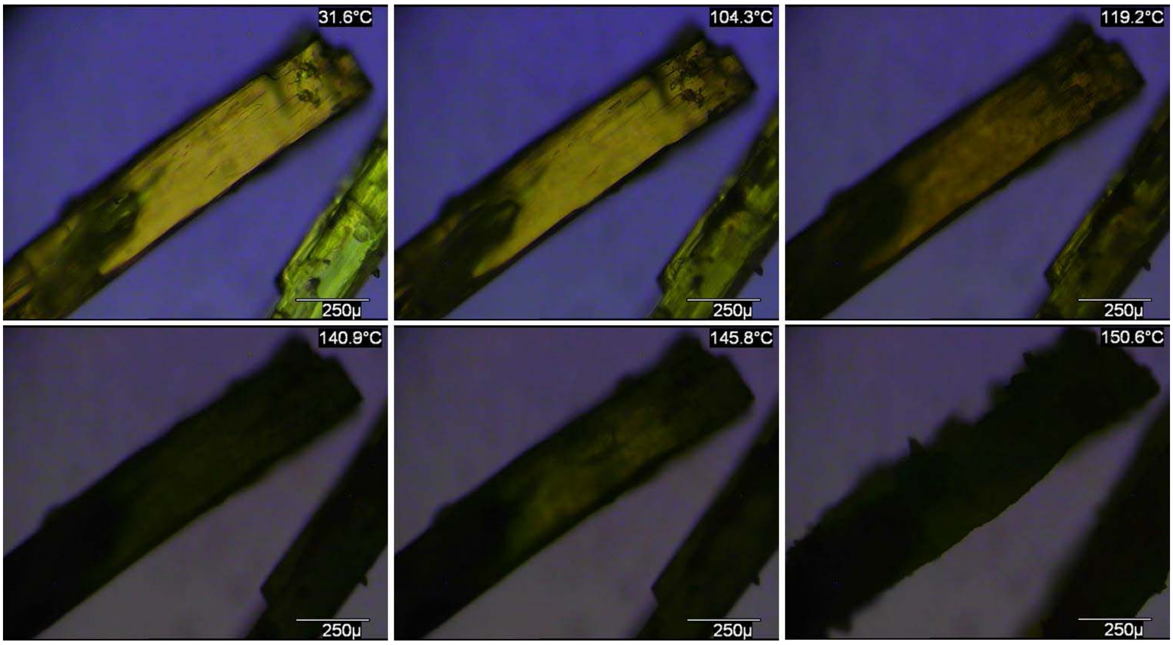

Figure S42 Optical micrographs of CBZ·FORM crystals at different temperatures under polarized light (heating rate of 10 °C.min^{-1}).

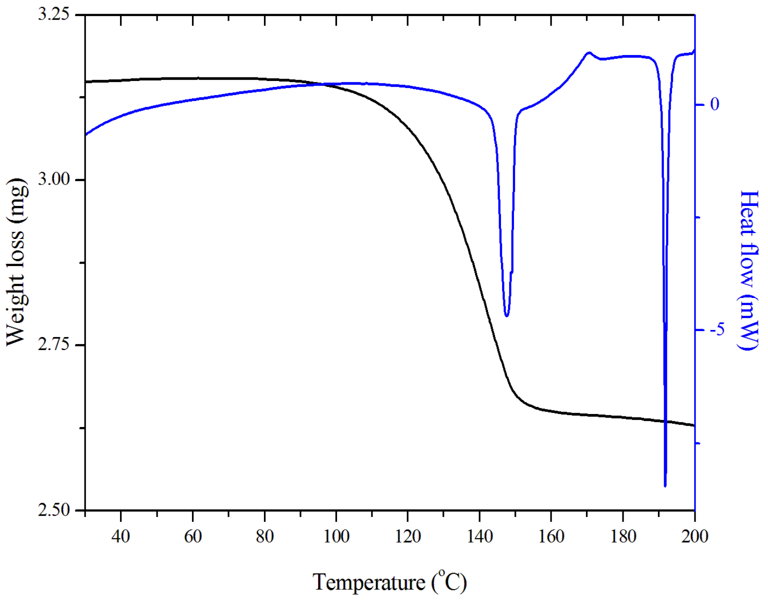

Figure S43DSC and TGA thermal characterization of CBZ·FORM crystals (10 °C.min^{-1}, perforated lid, N_2).

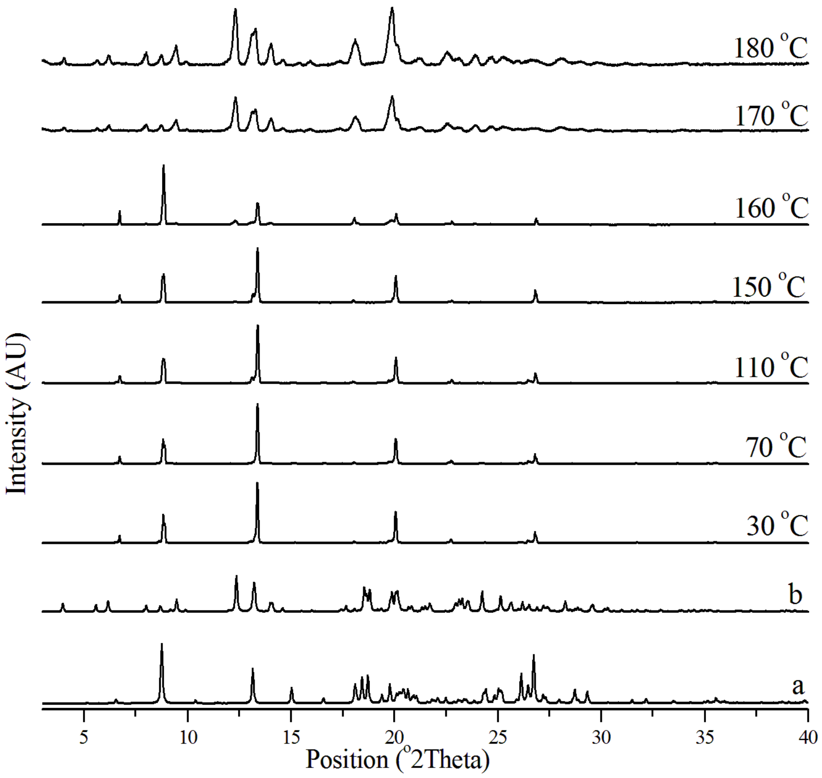

Figure S44*In situ* variable temperature PXRD patterns of CBZ·FORM crystals (10 ºC.min^{-1}, 25-180 ºC). *a*: CBZ·FORM from UNIBOI. *b*: CBZ Form I from CBMZPN11.

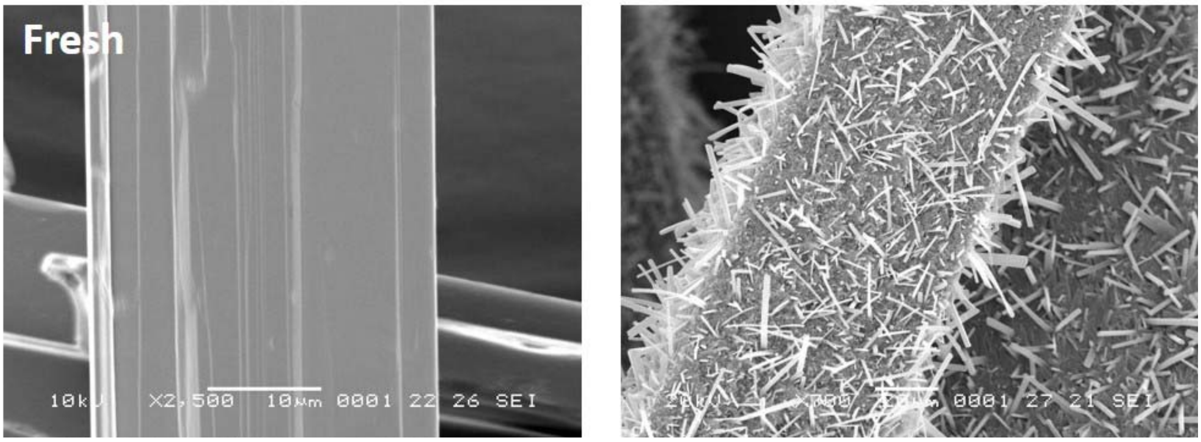

Figure S45SEM micrographs of CBZ·FORM crystals heated until 170 ºC (N_2, heating rate of 10 ºC.min^{-1}). The fresh surface is added for comparison purposes.

S3.9. CBZ dioxane solvate (2CBZ·DIOX), Group 3

TGA shows one single event of weight loss in the range 70-120 ºC. The heat flow curve characterizes desolvation as a relatively broad endothermic event ending at about 120 ºC. The DSC curve also

demonstrates baseline shifts in the range 150-170 °C, which resemble exothermic transformations, such as possible recrystallization.

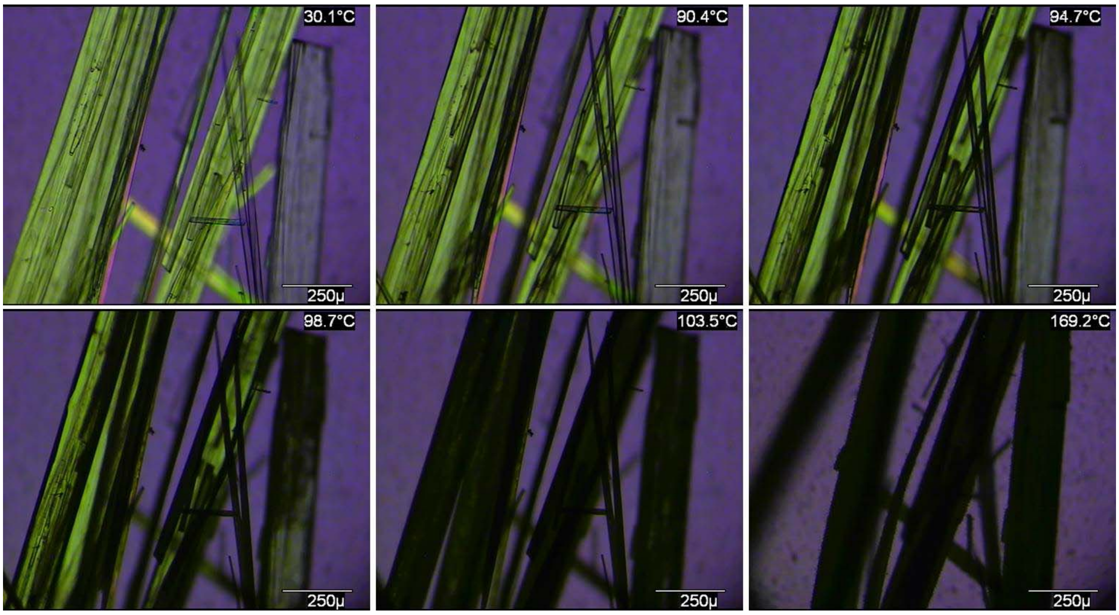

Figure S46 Optical micrographs of 2CBZ·DIOX crystals at different temperatures under polarized light (heating rate of 10 °C.min^{-1}).

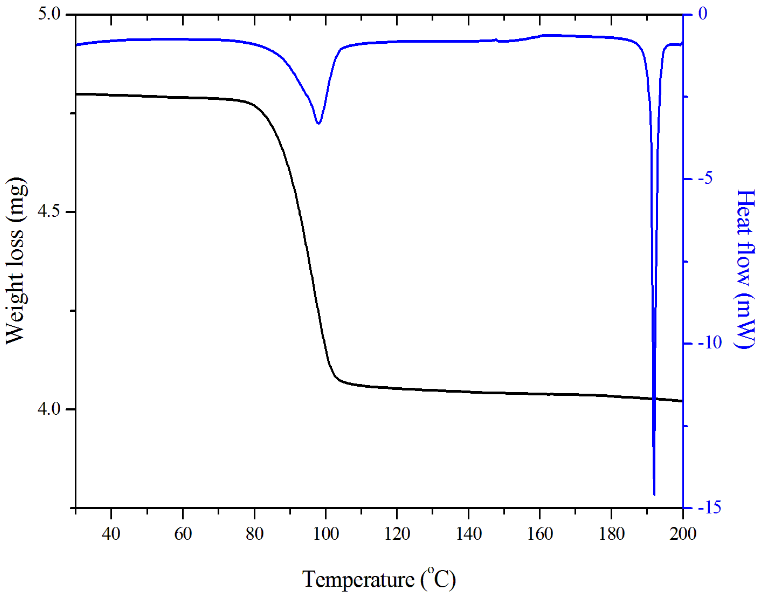

Figure S47DSC and TGA thermal characterization of 2CBZ·DIOX crystals (10 °C.min^{-1}, perforated lid, N_2).

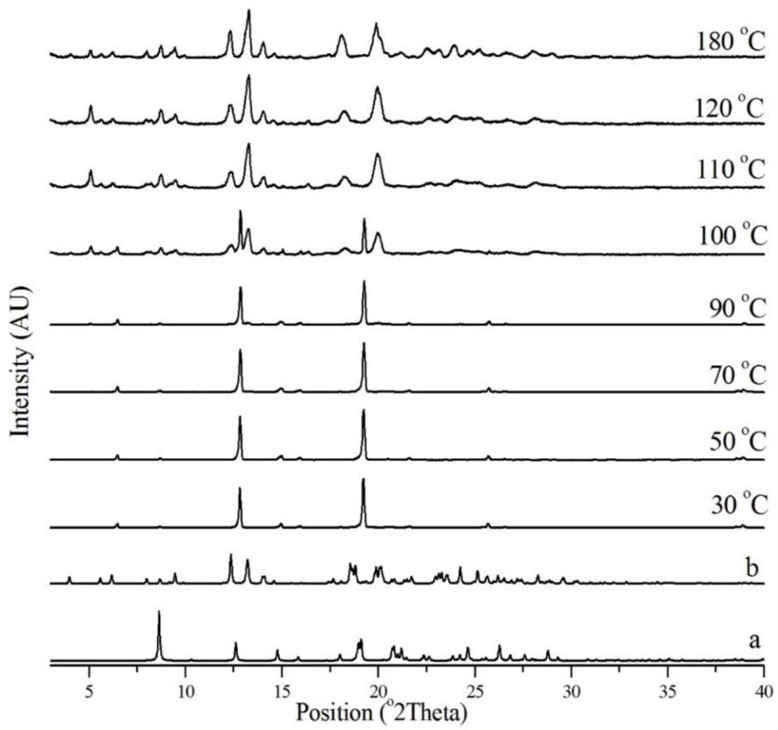

Figure S48*In situ* variable temperature PXRD patterns of 2CBZ·DIOX crystals (10 ºC.min^{-1}, 25-180 ºC). *a*: 2CBZ·DIOX from in-house data. *b*: CBZ Form I from CBMZPN11.

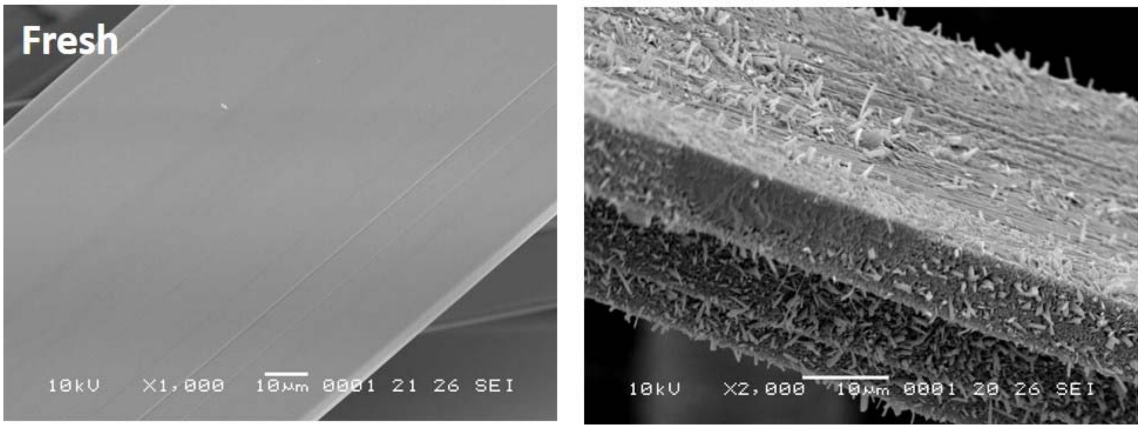

Figure S49SEM micrographs of 2CBZ·DIOX crystals heated until 120 ºC (N_2, heating rate of 10 ºC.min^{-1}). The fresh surface is added for comparison purposes.

S3.10. CBZ 2,2,2-trifluoroethanol solvates (CBZ·TFE and 2CBZ·TFE), Group 4

The TGA of CBZ·TFE demonstrates one event with two different slopes in the range 60-120 ºC, while the heat flow curve collected at 10 ºC.min^{-1} shows that desolvation is characterized by two unresolved endothermic events (T_{onset}=68.4 ºC). Although the shape of the thermograms alone could indicate the existence of an intermediate form during desolvation, the combination of thermal

analyses with other characterization techniques show that the findings only illustrate changes in the rate of desolvation. It is suggested that the recrystallization of CBZ at the final stages of desolvation may lead to a decrease in the rate of vaporization of TFE and desolvation of the remaining CBZ·TFE.

The DSC and TGA curves of the 2:1 solvate are different from the 1:1 form. TGA performed at 10 °C.min^{-1} shows a single weight loss in the range 80-130 °C, while the heat flow curve demonstrates that desolvation is characterized by an endothermic event with a T_{onset} of 97 °C.

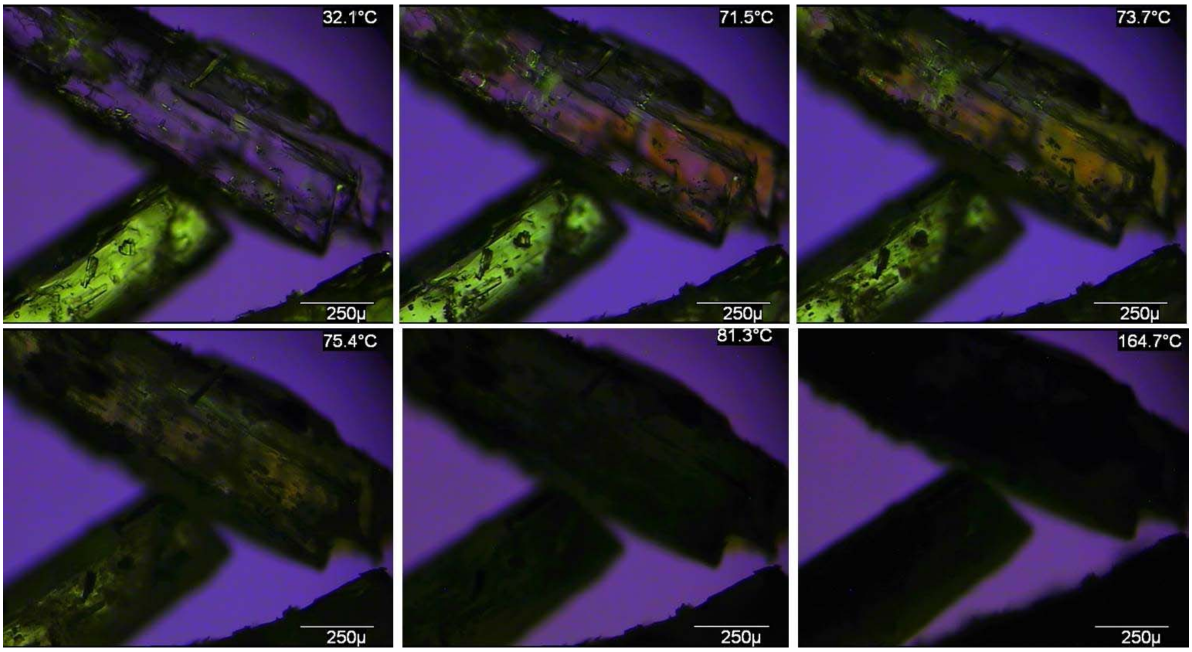

Figure S50 Optical micrographs of CBZ·TFE crystals at different temperatures under polarized light (heating rate of 10 ºC.min^{-1}).

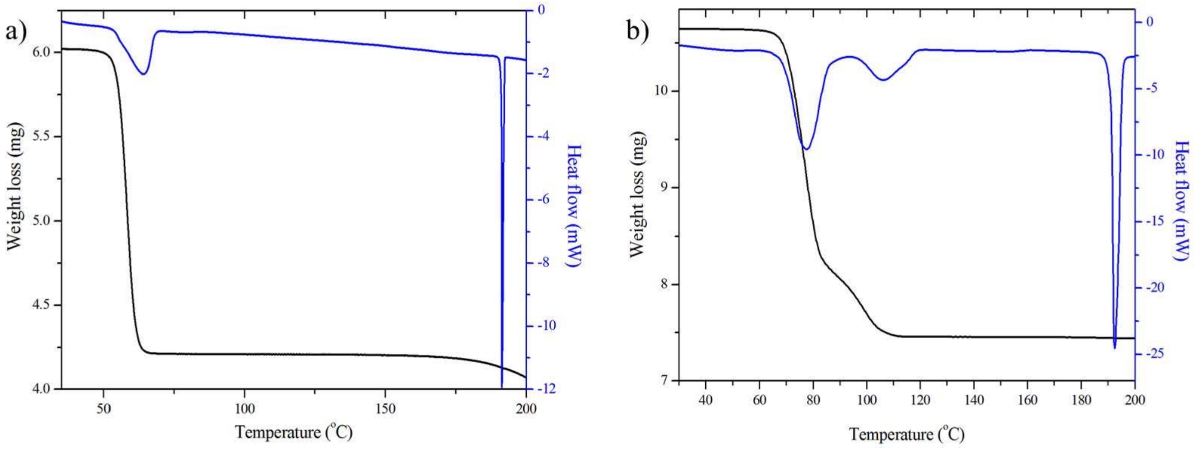

Figure S51DSC and TGA thermal characterisation of CBZ·TFE. *a:* 1 ºC.min^{-1}, perforated lid, N_2. *b*: 10 ºC.min^{-1}, perforated lid, N_2.

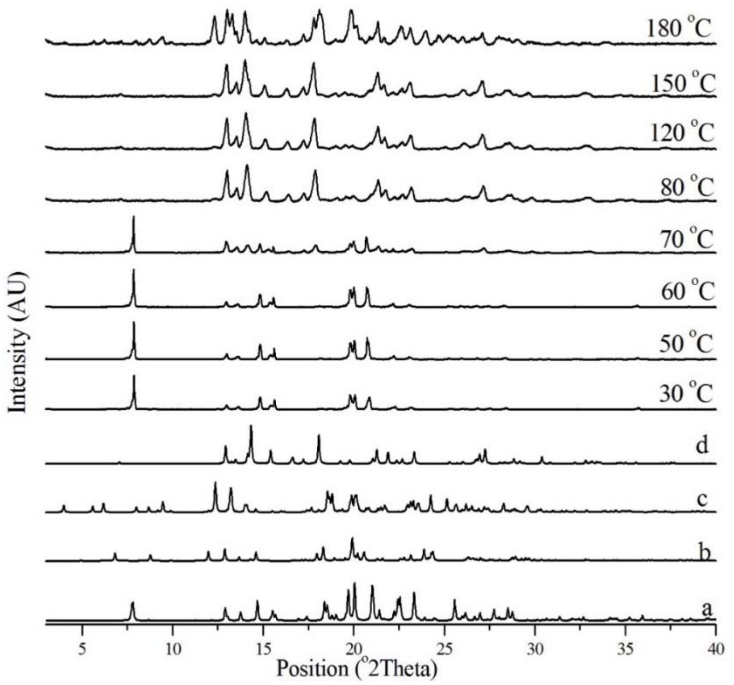

Figure S52*In situ* variable temperature PXRD patterns of CBZ·TFE crystals (10 ºC.min^{-1}, 25-180 ºC). *a*: CBZ·TFE from SAPDUJ. *b*: 2CBZ·TFE from in-house data. *c*: CBZ Form I from CBMZPN11. *d*: CBZ Form IV from CBMZPN12.

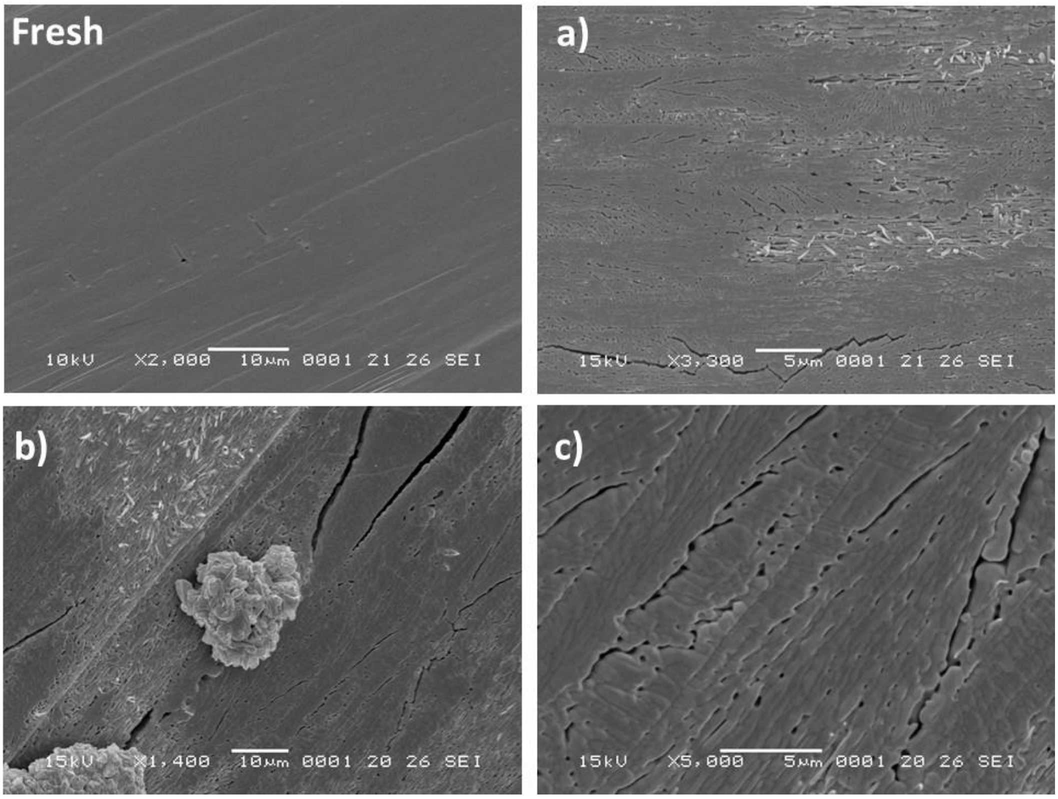

Figure S53SEM micrographs of CBZ·TFE crystals subjected to desolvation (the fresh surface is added for comparison purposes). *a* and *b*: heated until 120 ºC (N_2, 10 ºC.min^{-1}). *c*: heated until 80 ºC (N_2, heating rate of 10 ºC.min^{-1}).

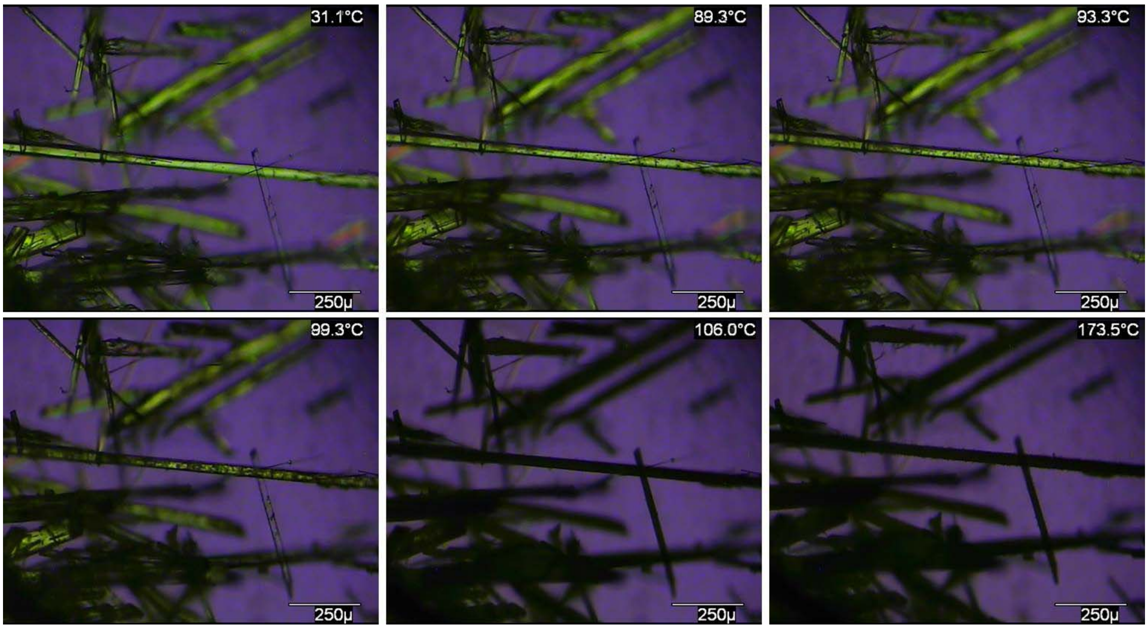

Figure S54 Optical micrographs of 2CBZ·TFE crystals at different temperatures under polarized light (heating rate of 10 °C.min^{-1}).

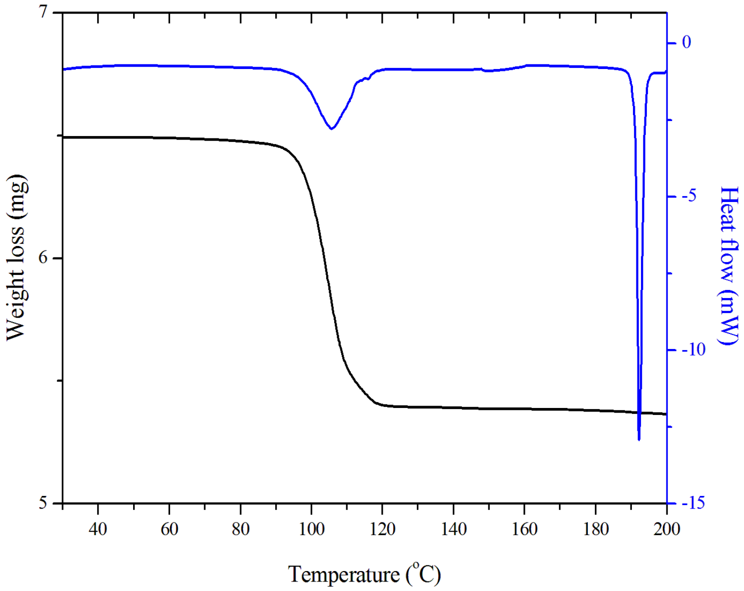

Figure S55DSC and TGA thermal characterization of 2CBZ·TFE crystals (10 °C.min^{-1}, perforated lid, N_2).

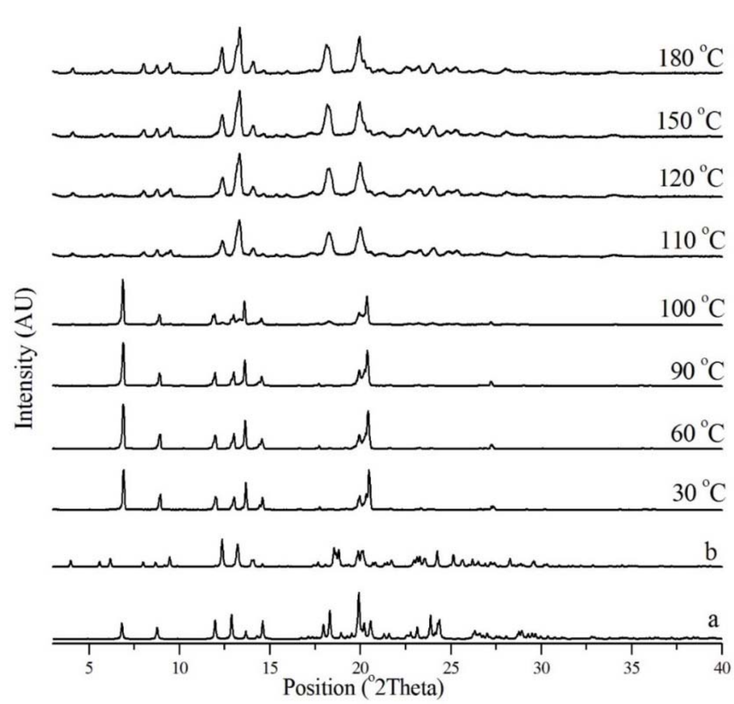

Figure S56*In situ* variable temperature PXRD patterns of 2CBZ·TFE crystals (10 ºC.min^{-1}, 25-180 ºC). *a*: 2CBZ·TFE from in-house data. *b*: CBZ Form I from CBMZPN11.

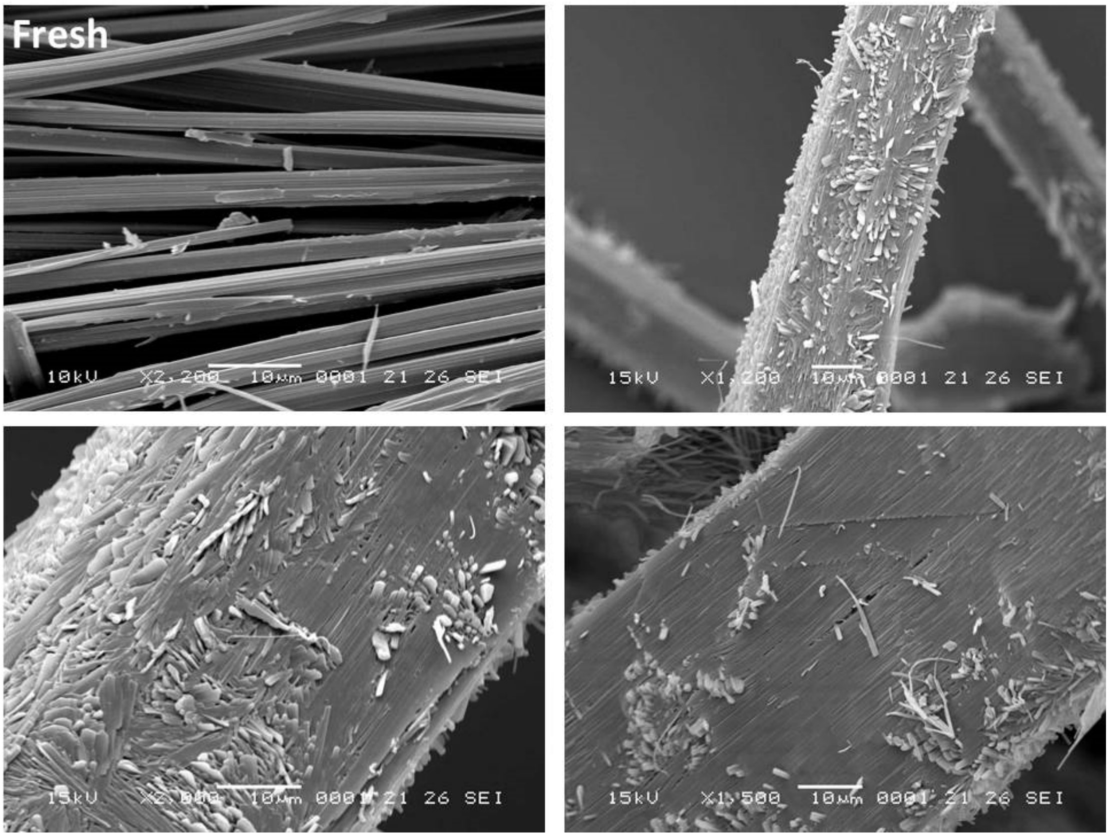

Figure S57SEM micrographs of 2CBZ·TFE crystals heated until 130 ºC (N_2, heating rate of 10 ºC.min^{-1}). The fresh surface is added for comparison purposes.

S3.11. CBZ trifluoroacetic acid solvate (CBZ·TFA), Group 5

The heat flow curves collected at 10 ºC.min^{-1} show an endothermic event that starts as a sharp endotherm (T_{onset}=128.4 ºC) but is followed by a broad event finishing above 140 ºC. As the temperature increases, two additional endotherms are also observed. These events can be correlated to three regions of weight loss that do not present clear limits in the TGA curves collected at 10 ºC.min^{-1}: (i) from approximately 80 to 175 ºC; (ii) from approximately 175 to 275 ºC; and (iii) above 275 ºC. The region (i) is related to the desolvation of CBZ·TFA and the (ii) and (iii) regions correspond to the decomposition of CBZ. The events in (ii) and (iii) are shifted to lower temperatures, as previously observed for 2CBZ·OXA. In the case of CBZ·TFA, however, the slopes of the curves collected at 10 ºC.min^{-1} are not comparable to the characteristic slopes of CBZ decomposition (see **Figure S63** and **Figure S64**).

Another difference in the behaviour of 2CBZ·OXA and CBZ·TFA is the effect of the heating rate on their thermal characteristics. Although the thermograms of 2CBZ·OXA do not differ whether they are collected at 1 or 10 ºC.min^{-1}, the CBZ·TFA thermograms do. At 1 ºC.min^{-1} the TFA solvate shows three regions of weight loss: (i) from approximately 60 to 115 ºC; (ii) from approximately 160 to 230 ºC; and (iii) from approximately 230 to 275 ºC. The regions (ii) and (iii) clearly correspond to the decomposition of CBZ, as seen in the thermal analyses of CBZ polymorphs. In contrast to the experiments performed at 10 ºC.min^{-1}, the desolvation event resulting from non-isothermal heating at 1 ºC.min^{-1} is clearly separated from the other weight loss events. The DSC curves collected at 1 ºC.min^{-1} combine the desolvation events to one endothermic process which is moderately asymmetric (T_{onset}=120.5 ºC). A second endotherm is also observed at higher temperatures between regions (i) and (ii) (T_{onset}=145.3 ºC).

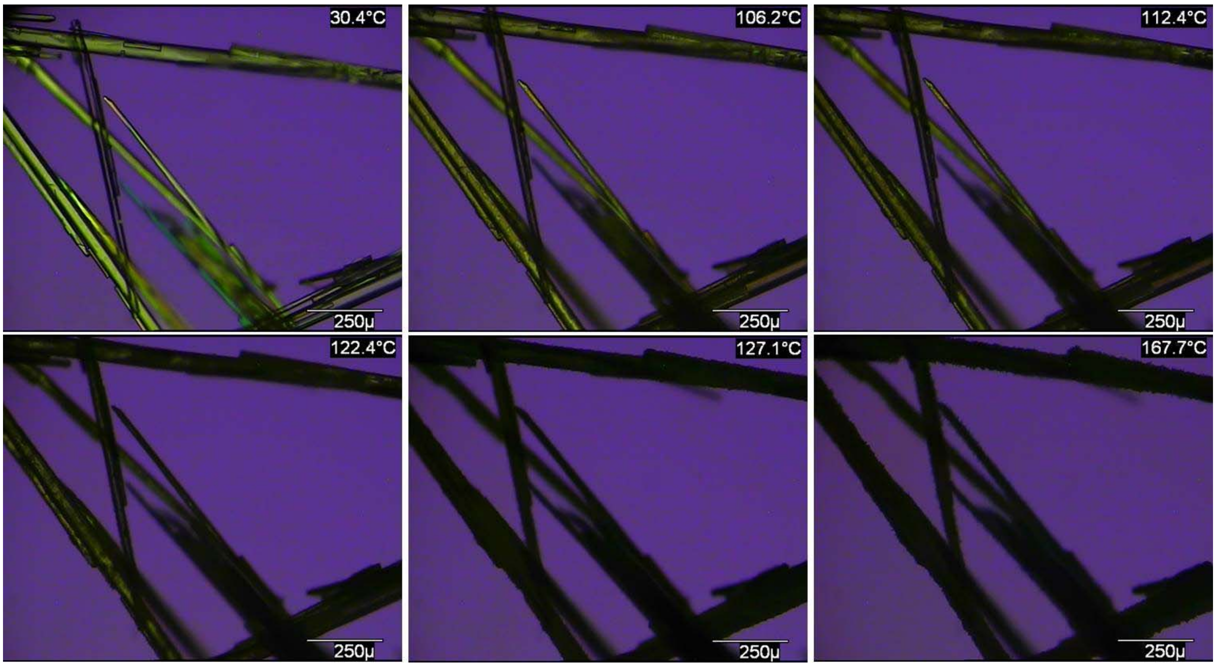

Figure S58 Optical micrographs of CBZ·TFA crystals at different temperatures under polarized light (heating rate of 10 °C.min^{-1}).

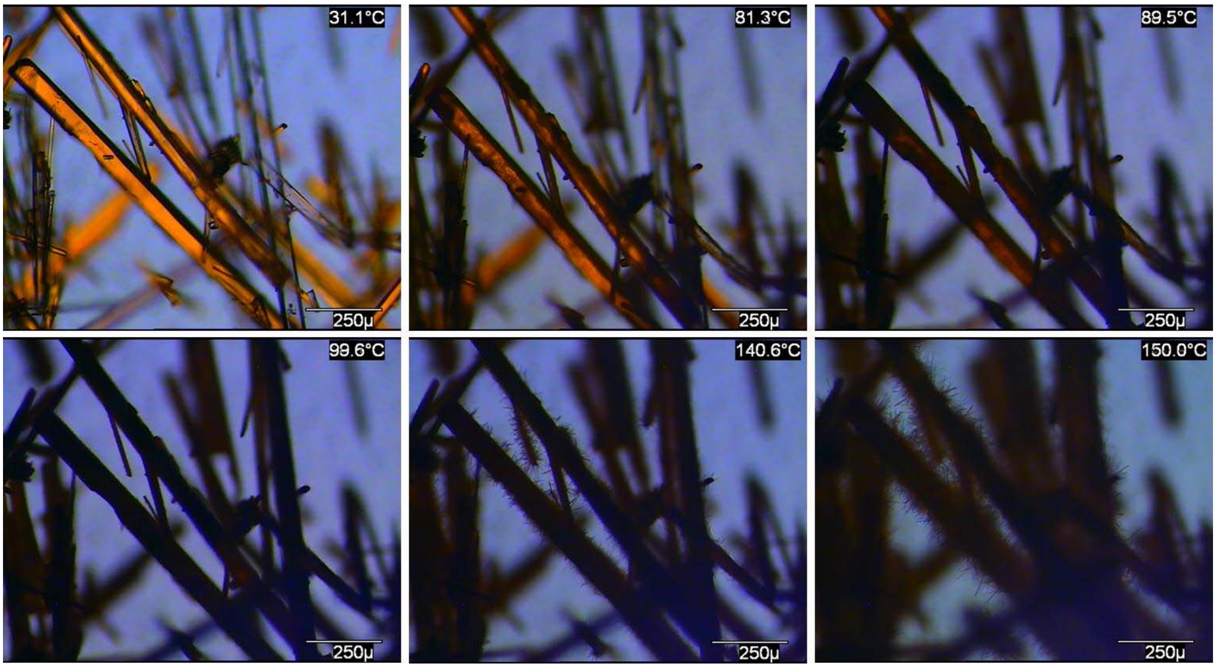

Figure S59 Optical micrographs of CBZ·TFA crystals at different temperatures under polarized light (heating rate of 1 °C.min^{-1}).

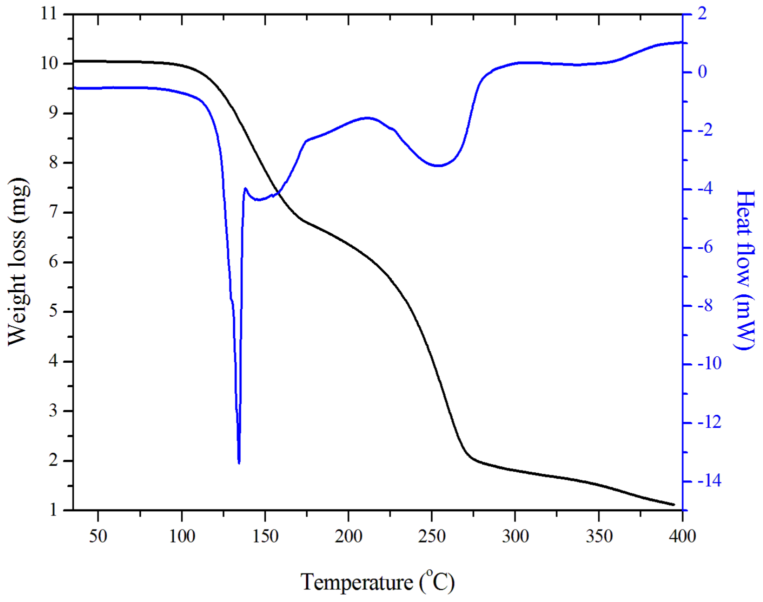

Figure S60DSC and TGA thermal characterization of CBZ·TFA crystals (10 ºC.min^{-1}, perforated lid, N_2).

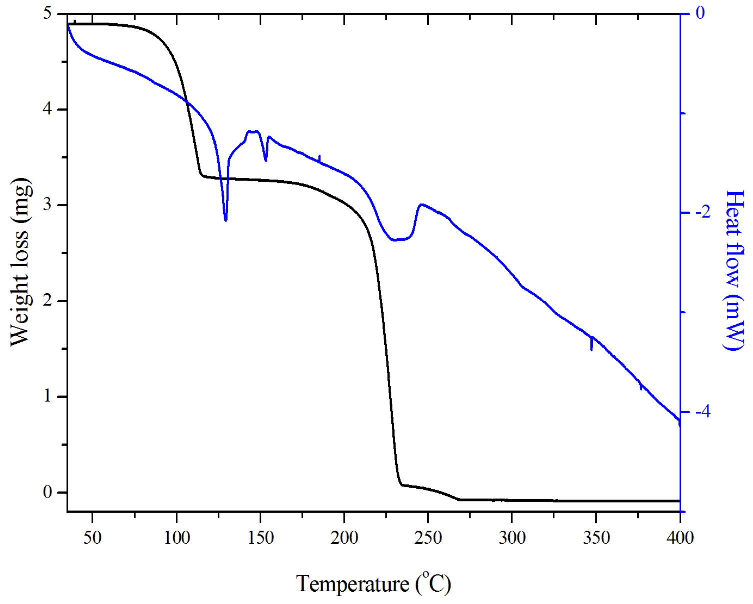

Figure S61DSC and TGA thermal characterization of CBZ·TFA crystals (1 ºC.min^{-1}, perforated lid, N_2).

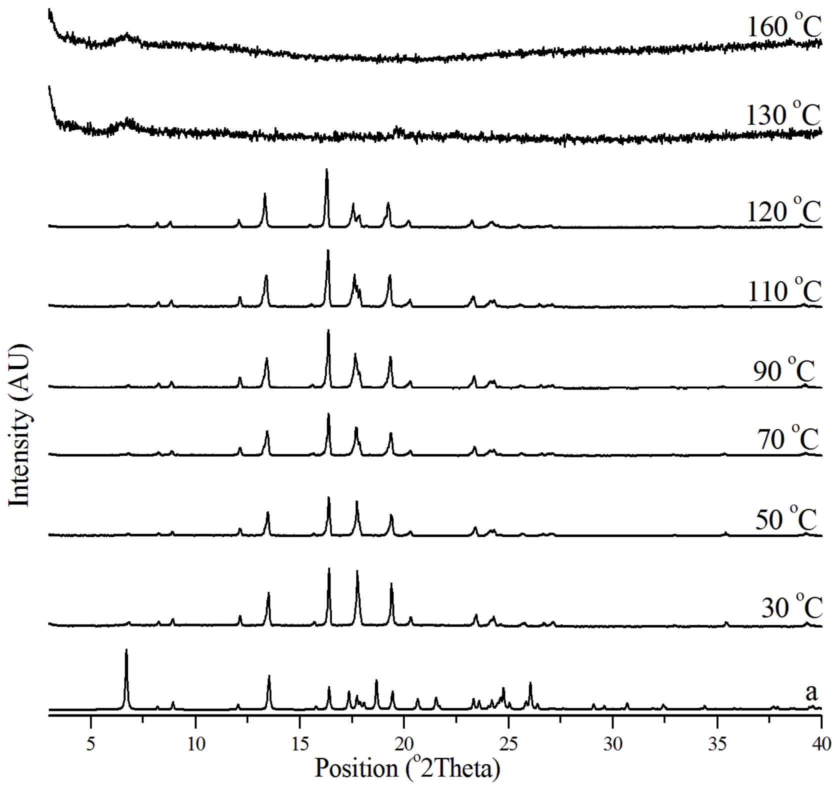

Figure S62 *In situ* variable temperature PXRD patterns of CBZ·TFA crystals (10 °C.min^{-1}, 25-180 °C). *a*: CBZ·TFA from GINFOZ.

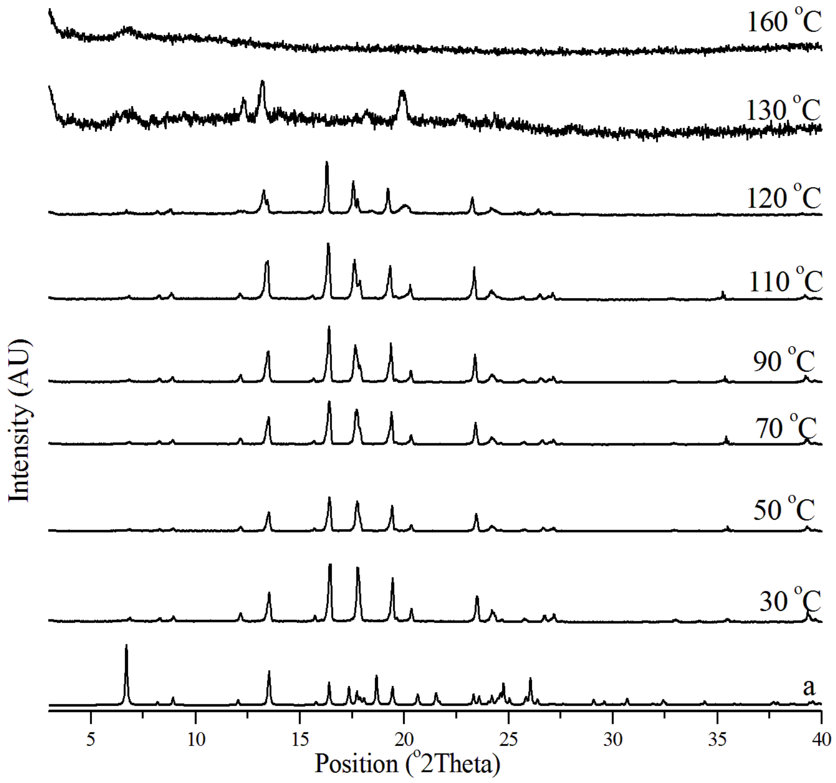

Figure S63 *In situ* variable temperature PXRD patterns of CBZ·TFA crystals (1 °C.min^{-1}, 25-180 °C). *a*: CBZ·TFA from SAPDUJ.

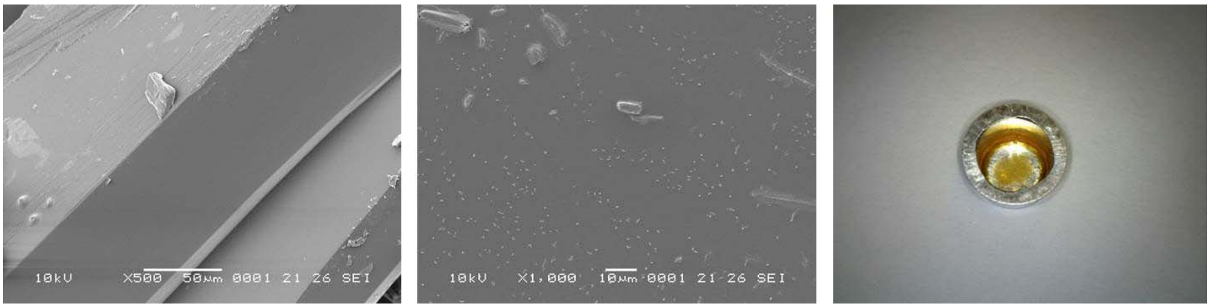

Figure S64SEM micrographs of CBZ·TFA crystals. The photograph on the right show the material which resulted from heating until 140 ºC (N_2, heating rate of 10 ºC.min^{-1}).

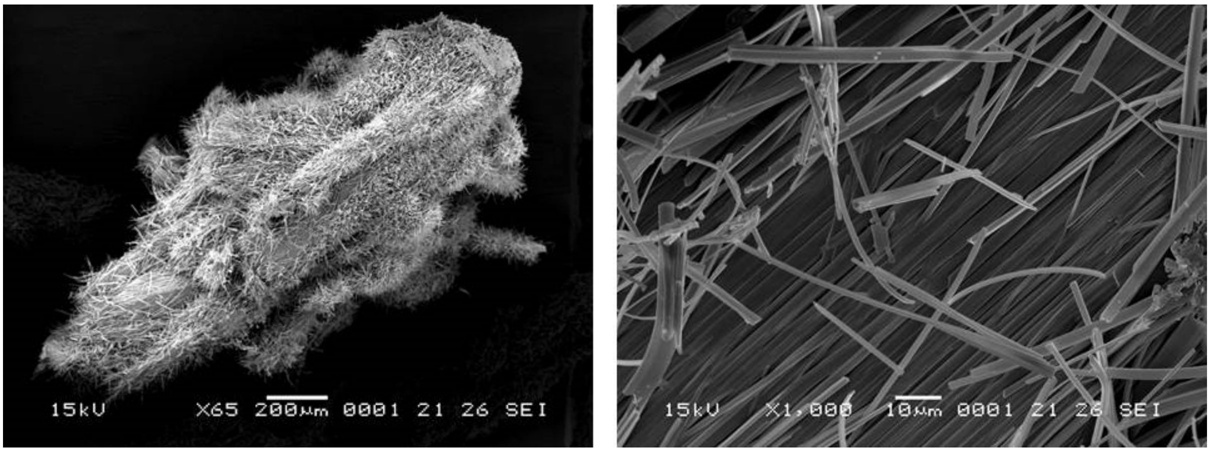

Figure S65SEM micrographs of CBZ·TFA crystals heated until 140 ºC (N_2, heating rate of 1 ºC.min^{-1}).

S3.12. CBZ acetic acid solvate (CBZ·ACA), Group 5

A single weight loss is observed in the range 80-140 ºC. The heat flow curve associates desolvation with one event (T_{onset}=125.6 ºC), which starts as a broad endotherm but becomes sharper as the temperature increases. This characteristic may be correlated to the existence of concomitant events, such as desolvation and melting or the formation of a peritectic mixture. The thermograms show a minor exothermic event after the endotherm assigned to desolvation.

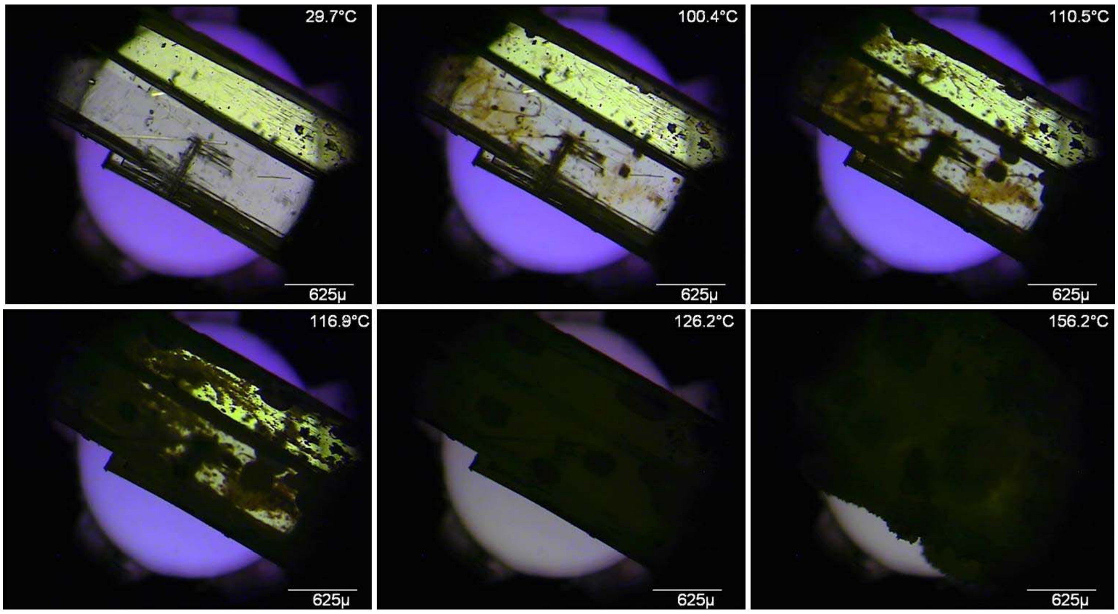

Figure S66 Optical micrographs of CBZ·ACA crystals at different temperatures under polarized light (heating rate of 10 °C.min^{-1}).

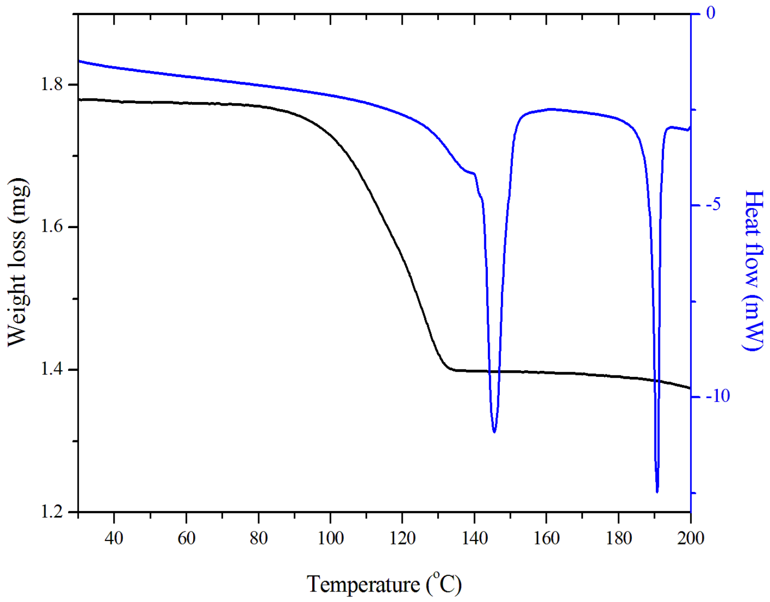

Figure S67DSC and TGA thermal characterization of CBZ·ACA crystals (10 °C.min^{-1}, perforated lid, N_2).

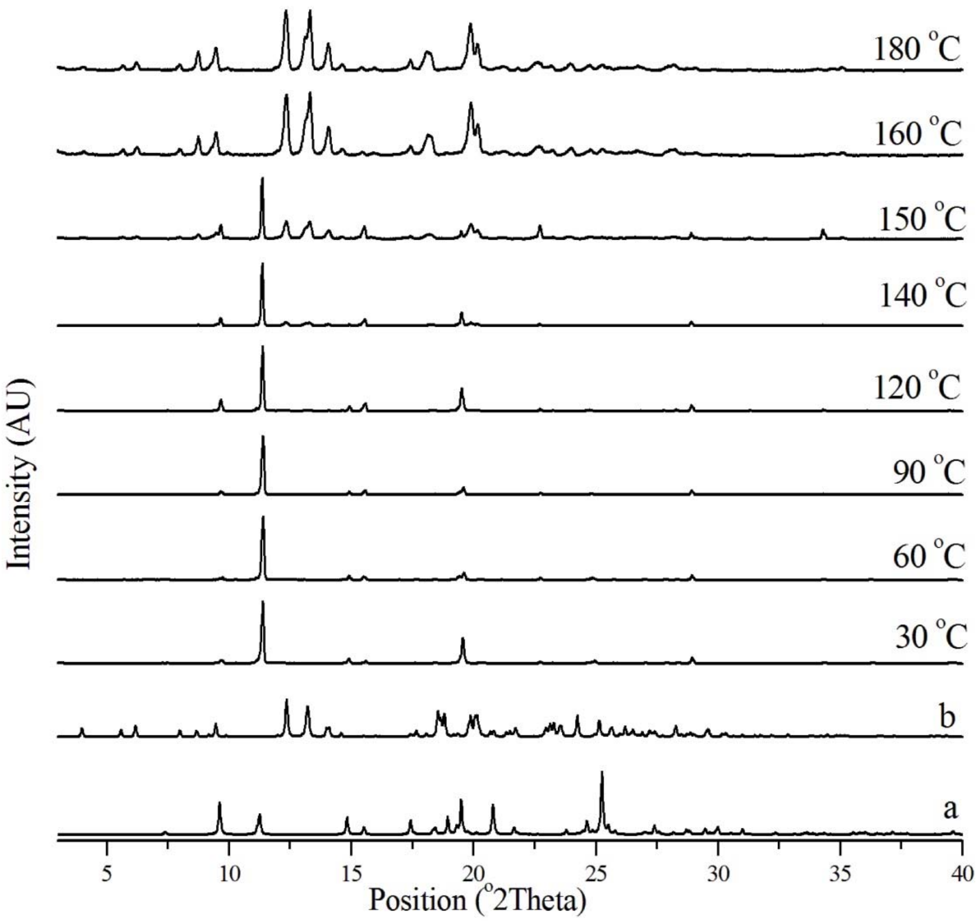

Figure S68 *In situ* variable temperature PXRD patterns of CBZ·ACA crystals (10 ºC.min^{-1}, 25-180 ºC). *a*: CBZ·ACA from UNEZIW. *b*: CBZ Form I from CBMZPN11.

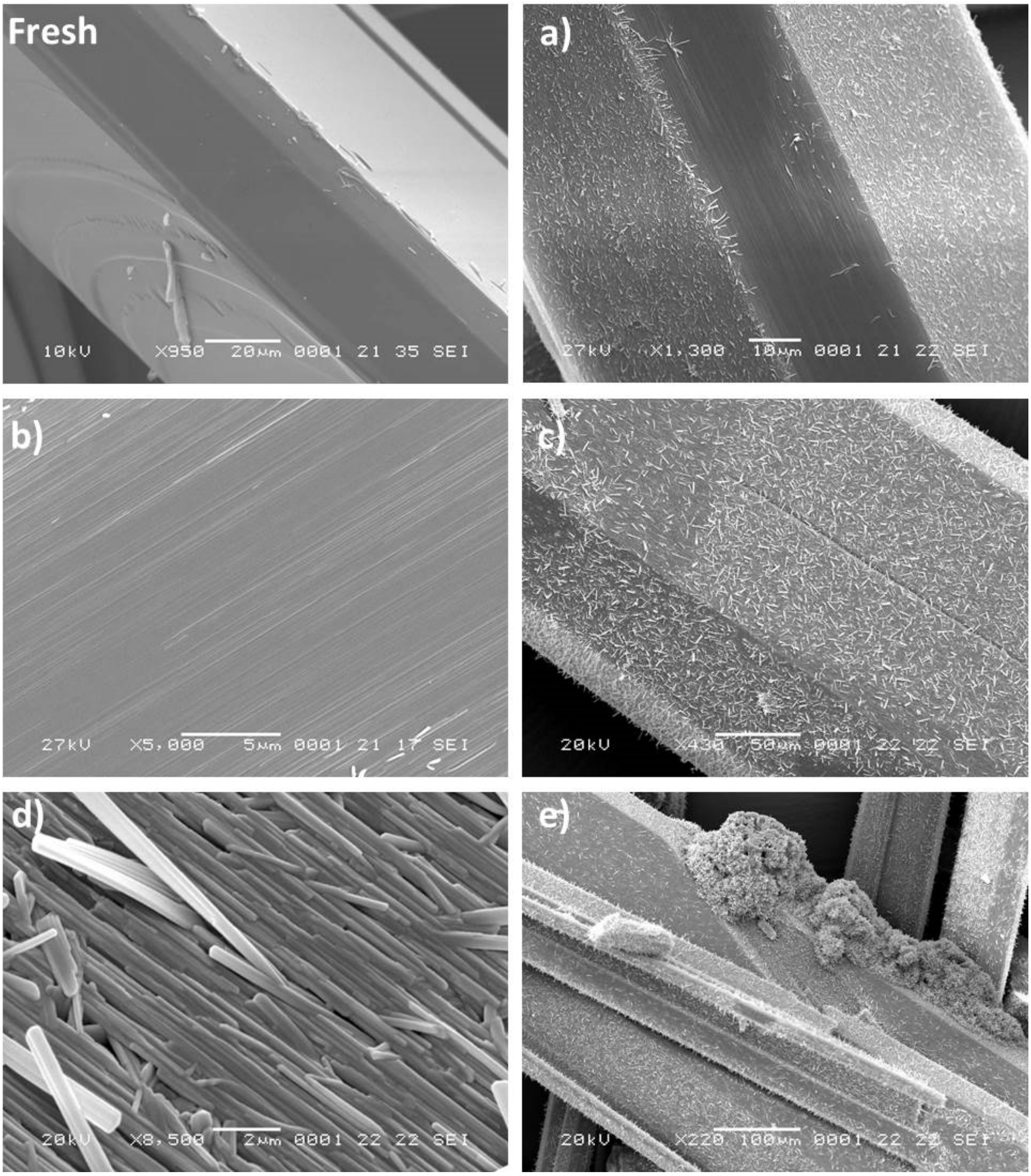

Figure S69SEM micrographs of CBZ:ACA crystals subjected to desolvation (the fresh surface is added for comparison purposes). *a, b* and *c*: heated until 100 ºC (N_2, 10 ºC.min^{-1}). *d* and *e*: heated until 150 ºC (N_2, heating rate of 10 ºC.min^{-1}).

S3.13. CBZ formic acid solvate (CBZ·FA), Group 5

A single weight loss is observed in the range 100-160 ºC. The heat flow curve shows desolvation as one endothermic event (T_{onset}=119.0 ºC) associated with other events, which may represent desolvation and melting or formation of a peritectic mixture.

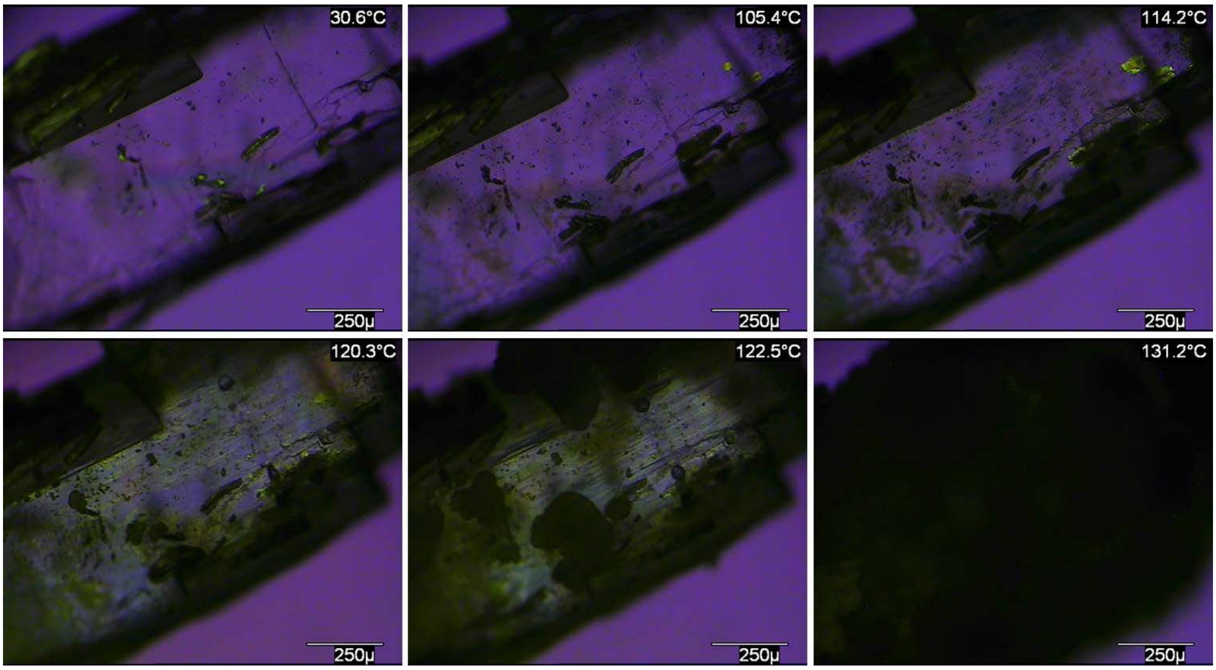

Figure S70 Optical micrographs of CBZ·FA crystals at different temperatures under polarized light (heating rate of 10 °C.min^{-1}).

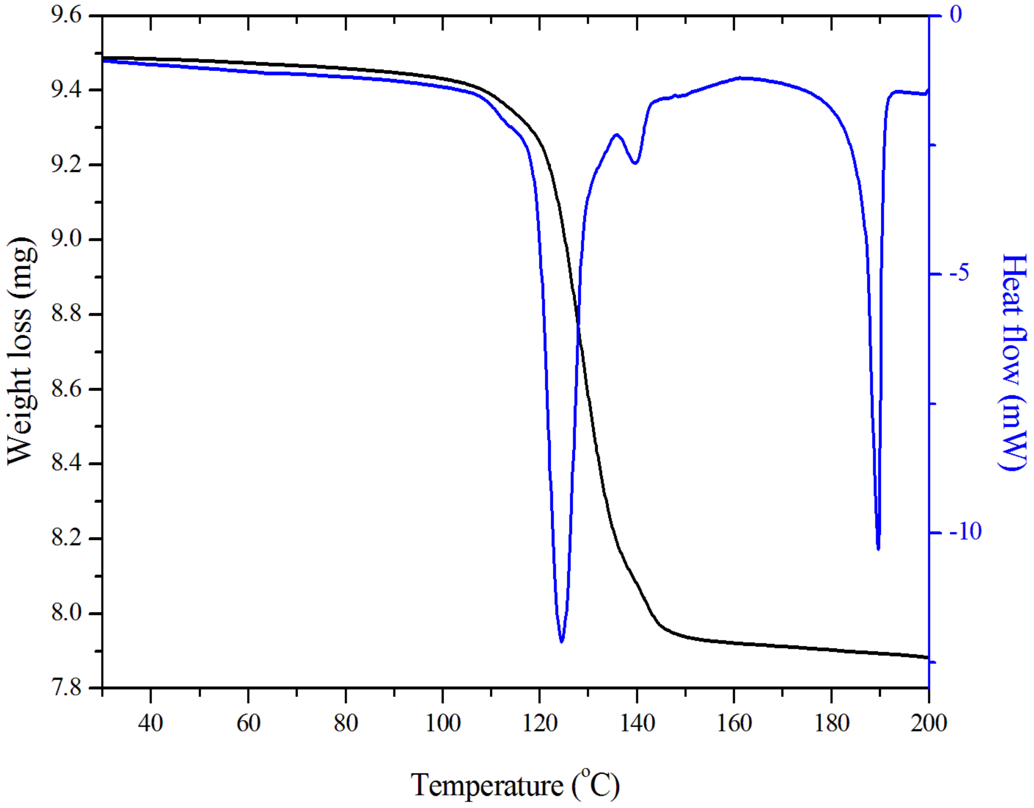

Figure S71DSC and TGA thermal characterization of CBZ·FA crystals (10 °C.min^{-1}, perforated lid, N_2).

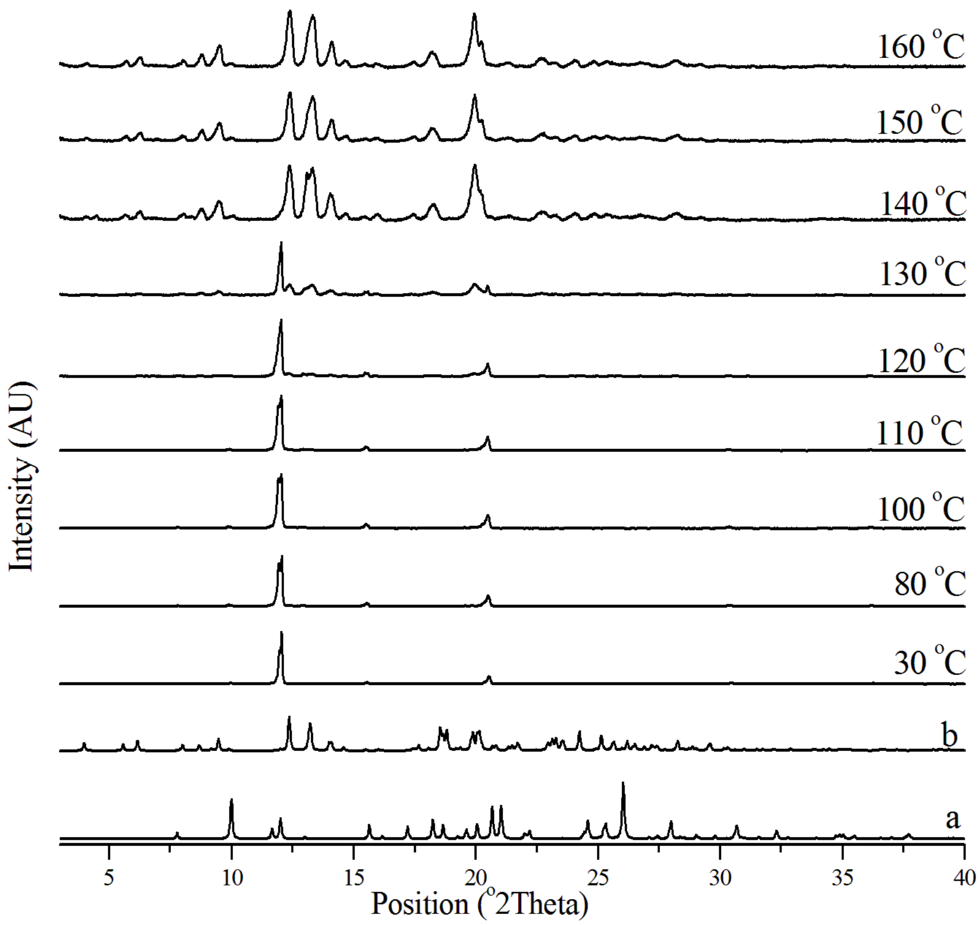

Figure S72 *In situ* variable temperature PXRD patterns of CBZ·FA crystals (10 ºC.min^{-1}, 25-180 ºC). *a*: CBZ·FA from UNEZOC. *b*: CBZ Form I from CBMZPN11.

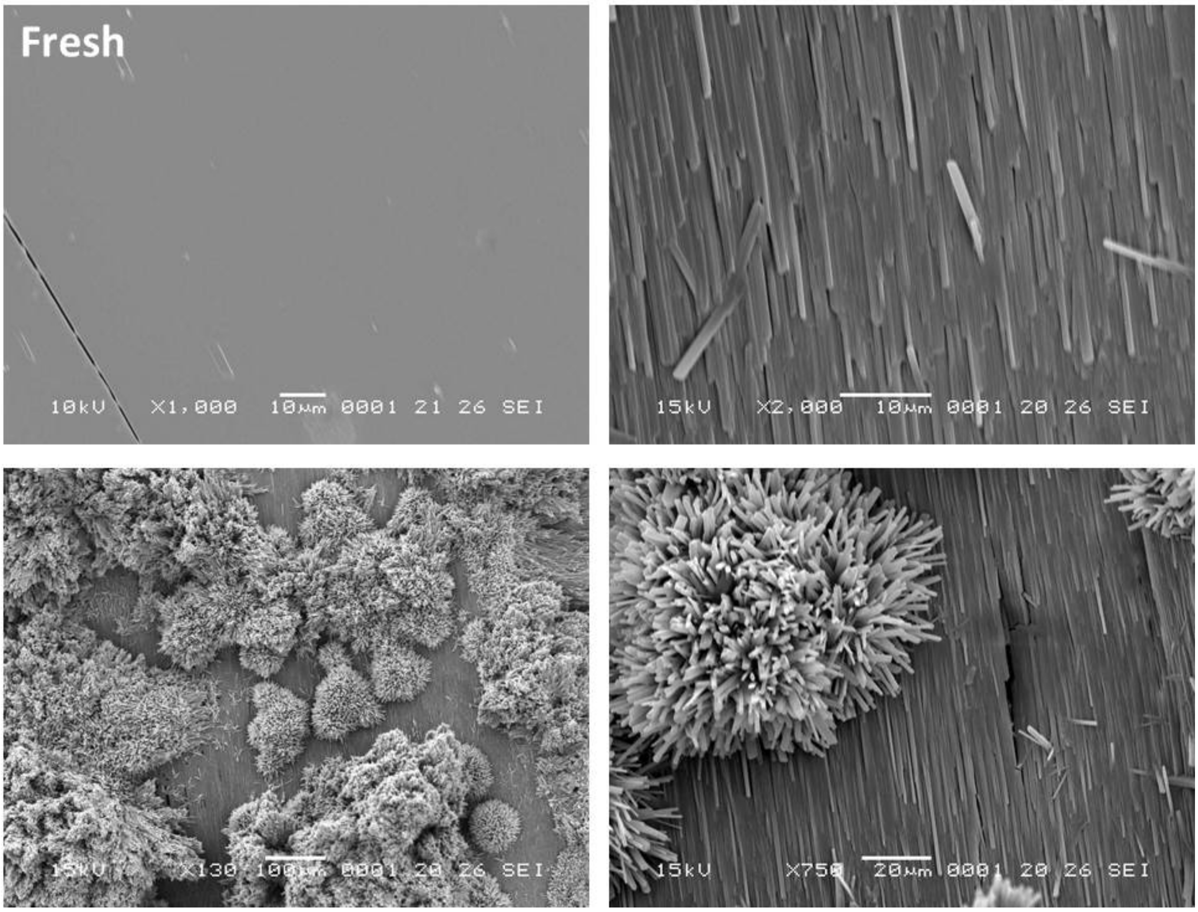

Figure S73SEM micrographs of CBZ·FA crystals heated until 165 ºC (N_2, heating rate of 10 ºC.min^{-1}). The fresh surface is added for comparison purposes.

S3.14. Additional thermal behaviour analyses on carbamazepine polymorphs I and III

The single crystals of CBZ polymorph I melt at about 190 ºC and no other transformation is seen by optical microscopy during heating. The single crystals of polymorph III, in turn, show additional events taking place at about 140 ºC. Interestingly, these events appeared to be different depending on the particle size. In the case of large particles of Form III, the crystals start fracturing above 145 ºC, while small particles show the formation of whiskers on the surface of the crystals. This transformation corresponds to the conversion of Form III into Form I, which is the most stable form at high temperatures. It is unclear whether these findings show that the mechanism of transformation is dependent on particle size, or if defects on the surface of small crystals may catalyse the growth of whiskers. In other words, it may indicate that CBZ molecules sublime at high temperatures and recrystallize on the surface of the crystals of Form I, showing the characteristic whiskers. The literature has reported the formation of whiskers (O'Brien *et al.*, 2004; Khoo *et al.*, 2013), however, to the best of our knowledge, fractures during the transformation have not been previously reported.

The micrographs agree with the thermograms. The TGA analyses of CBZ polymorphs I and III show two events of weight loss above 200 ºC (10 ºC.min^{-1}). Just before the chemical decomposition of CBZ, the DSC curves of both polymorphs show one endotherm corresponding to melting at about 190 ºC. The fusion event is equivalent in both samples, Form I and Form III, and it characterizes the fusion of polymorph I (T_{onset}=189.3 ºC, T_{peak}=190.7 ºC, ΔH_{fusion}=114.88 kJ.g^{-1}; and T_{onset}=188.9 ºC, T_{peak}= 190.4 ºC, ΔH_{fusion}=119.3 kJ.g^{-1}, respectively). Similarly to the results obtained from *in situ* optical microscopy, the DSC analyses show that Form III converts into Form I with heating. In this case, the heat flow curves of Form III show a small endothermic event at around 150 ºC, which is correlated to the polymorphic transition.

Although it is well accepted in the literature that Form I is the most stable polymorph at high temperatures (Cruz-Cabeza *et al.*, 2007, 2006), the exact temperature of the transition from polymorph III to polymorph I is uncertain (Behme & Brooke, 1991; Griesser *et al.*, 1999; Krahn & Mielck, 1989; Umeda *et al.*, 1984; Rustichelli *et al.*, 2000). Aiming to test the transition temperatures described in previous studies, slurries of Form I or Form III were prepared in 1-butanol at 25 ºC, 80 ºC and 120 ºC (reflux). 1-butanol was selected as a solvent because it does not form a solvate with CBZ and it has high boiling point. The slurries were equilibrated for 72 hours, filtered and the solids were analysed by PXRD. *Ex situ* PXRD patterns of the samples after slurrying show that all the samples converted to Form III (minor quantities of Form II are detected in some experiments). These results show that Form III only converts into Form I at temperatures above 120 ºC.

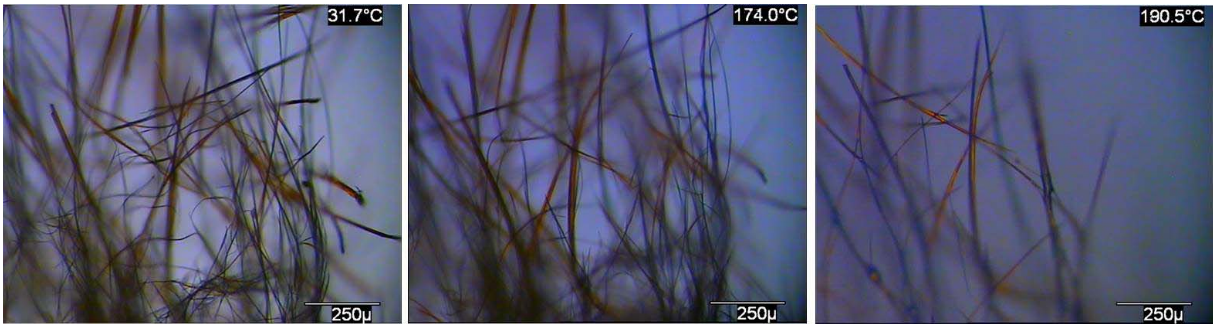

Figure S74 Optical micrographs of CBZ Form I crystals at different temperatures under polarized light (heating rate of 10 ºC.min^{-1}).

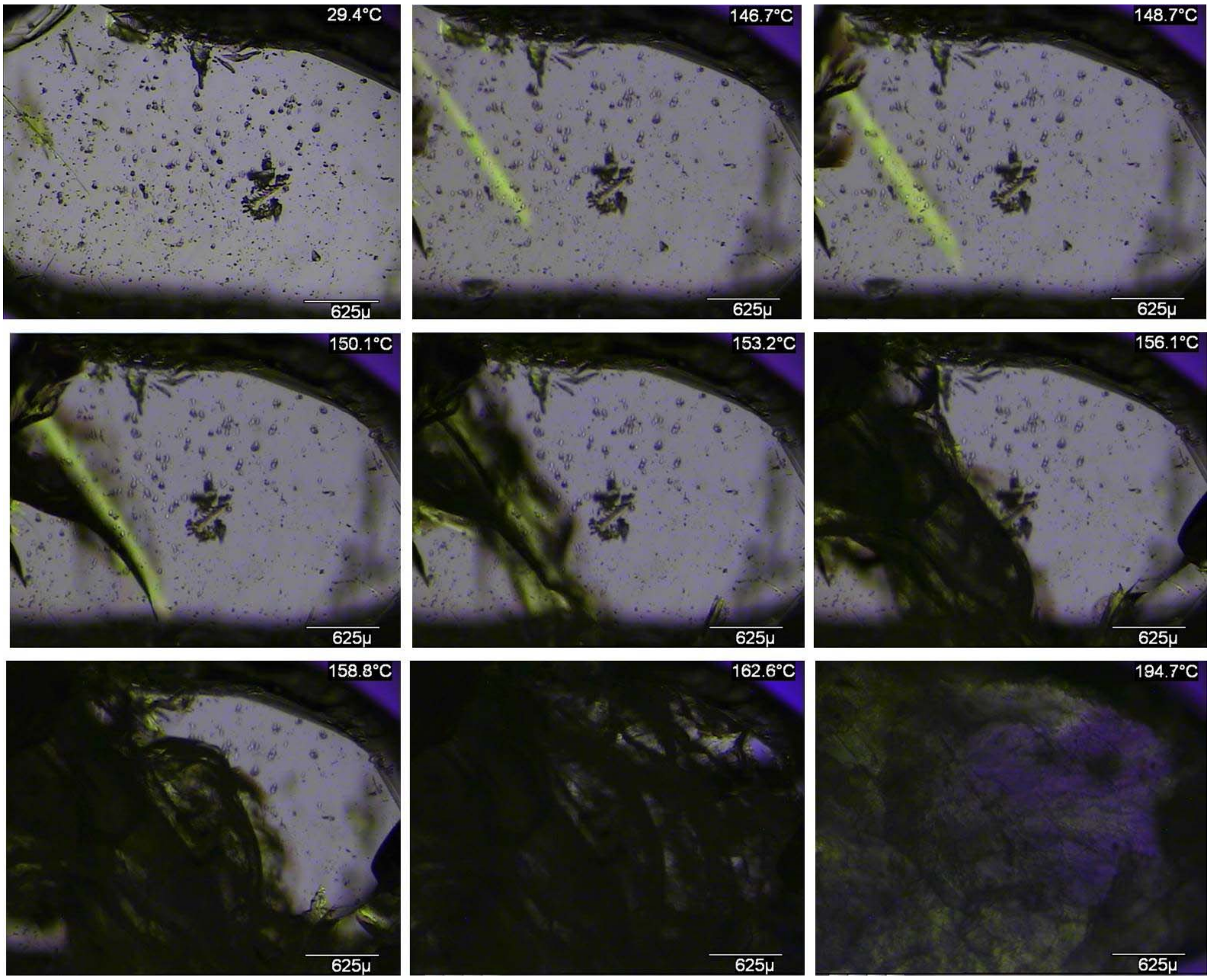

Figure S75 Optical micrographs of CBZ Form III large crystals at different temperatures under polarized light (heating rate of 10 ºC.min^{-1}).

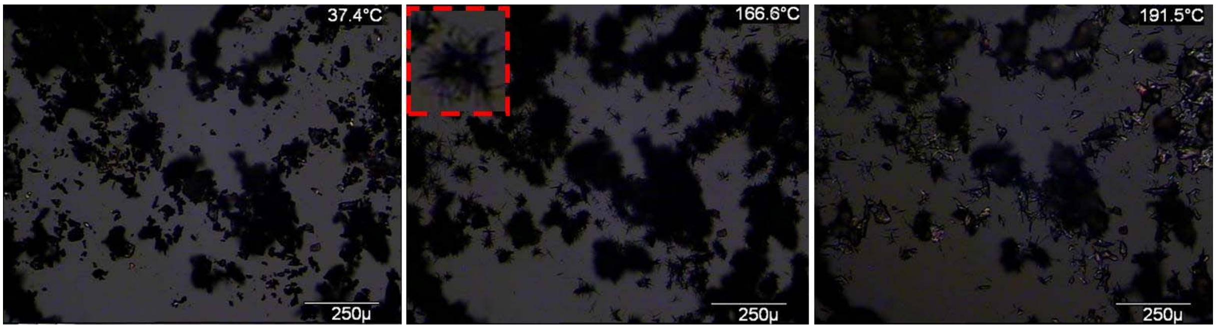

Figure S76 Optical micrographs of CBZ Form III small crystals at different temperatures under polarized light (heating rate of 10 ºC.min^{-1}). The detail is a zoomed area showing the whiskers.

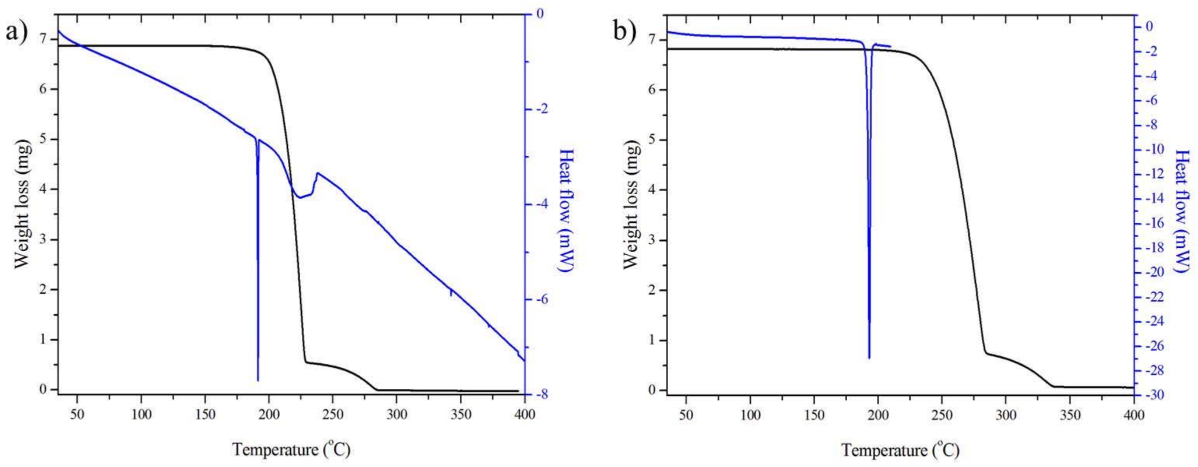

Figure S77DSC and TGA thermal characterisation of CBZ Form I. *a:* 1 °C.min^{-1}, perforated lid, N_2. *b*: 10 °C.min^{-1}, perforated lid, N_2.

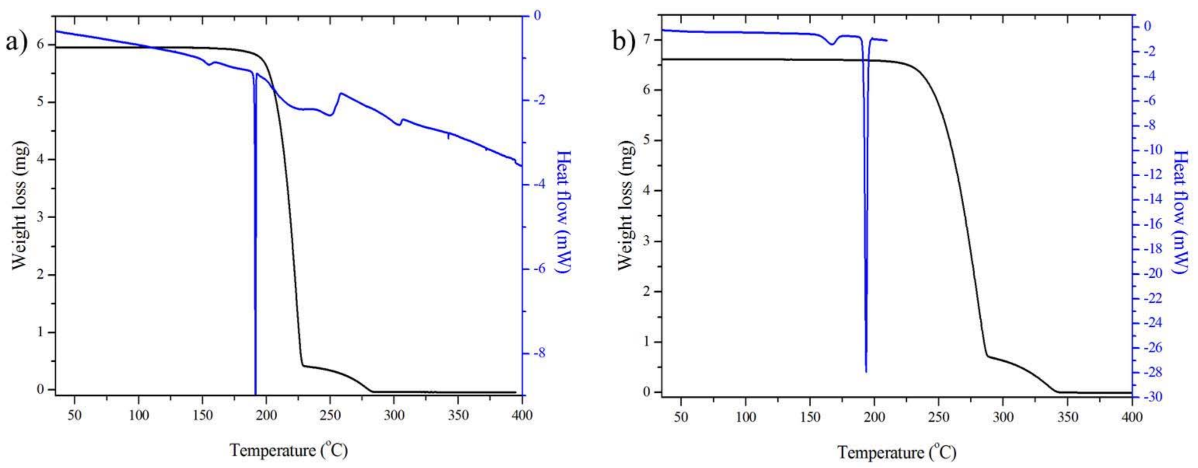

Figure S78DSC and TGA thermal characterisation of CBZ Form III. *a:* 1 °C.min^{-1}, perforated lid, N_2. *b*: 10 °C.min^{-1}, perforated lid, N_2.

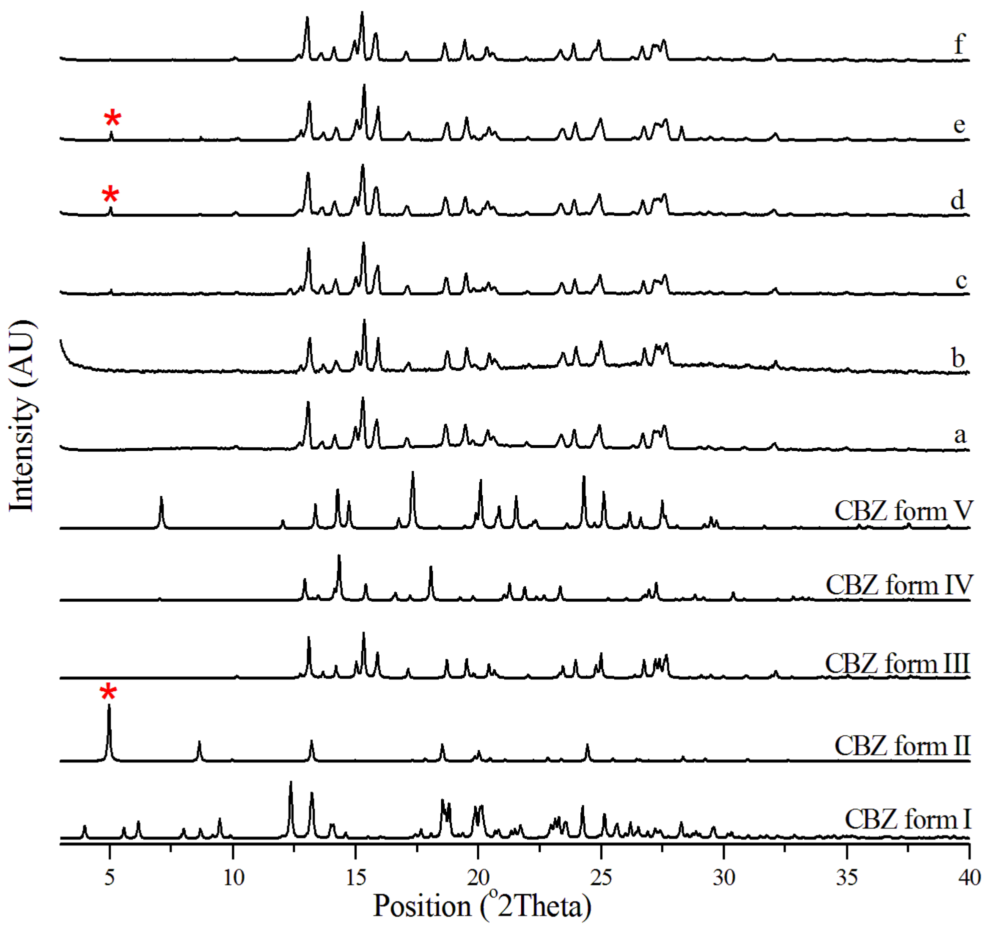

Figure S79PXRD patterns of the CBZ samples subjected to slurrying in 1-butanol at different temperatures for 72 hours. The starting material in *a*, *c*, and *e* was polymorph I, and the experimental temperatures were 25, 80 and 120 °C, respectively. The starting material in *b*, *d*, and *f* was polymorph III, and the experimental temperatures were 25, 80 and 120 °C, respectively. The calculated patterns of CBZ anhydrous polymorphs I, II, III, IV and V are added for comparison, and peaks of Form II are highlighted.

S4. References

Arlin, J.-B., Price, L. S., Price, S. L. & Florence, A. J. (2011). *Chem. Commun. (Camb).* **47**, 7074–7076.

Behme, R. J. & Brooke, D. (1991). *J. Pharm. Sci.* **80**, 986–990.

Childs, S. L., Wood, P. A., Rodriguez-Hornedo, N., Reddy, L. S. & Hardcastle, K. I. (2009). *Cryst. Growth Des.* **9**, 1869–1888.

Cruz-Cabeza, A. J., Day, G. M. & Jones, W. (2011). *Phys. Chem. Chem. Phys.* **13**, 12808–12816.

Cruz-Cabeza, A. J., Day, G. M. & Motherwell, W. D. S. (2006). *Cryst. Growth Des.* **5**, 1–31.

Cruz-Cabeza, A. J., Day, G. M., Motherwell, W. D. S. & Jones, W. (2007). *Cryst. Growth Des.* **7**, 100–107.

Fernandes, P., Bardin, J., Johnston, A., Florence, A. J., Leech, C. K., David, W. I. F. & Shankland, K. (2007). *Acta Crystallogr. Sect. E Struct. Reports Online*. **63**, o4269–o4269.

Fleischman, S. G., Kuduva, S. S., McMahon, J. a., Moulton, B., Bailey Walsh, R. D., Rodríguez-Hornedo, N. & Zaworotko, M. J. (2003). *Cryst. Growth Des.* **3**, 909–919.

Good, D. J. & Rodríguez-Hornedo, N. (2010). *Cryst. Growth Des.* **10**, 1028–1032.

Griesser, U. J., Szelagiewicz, M., Pitt, C. & Cianferani, S. (1999). *J. Therm. Anal. Calorim.* **57**, 45–60.

Grzesiak, A. L., Lang, M., Kim, K. & Matzger, A. J. (2003). *J. Pharm. Sci.* **92**, 2260–2271.

Harris, R. K., Ghi, P. Y., Puschmann, H., Apperley, D. C., Griesser, U. J., Hammond, R. B., Ma, C., Roberts, K. J., Pearce, G. J., Yates, J. R. & Pickard, C. J. (2005). *Org. Process Res. Dev.* **9**, 902–910.

Johnston, A., Florence, A. J. & Kennedy, A. R. (2005). *Acta Crystallogr. Sect. E Struct. Reports Online*. **61**, o1509–o1511.

Johnston, A., Johnston, B. F., Kennedy, A. R. & Florence, A. J. (2008). *CrystEngComm*. **10**, 23–25.

Khoo, J. Y., Shah, U. V., Schaepertoens, M., Williams, D. R. & Heng, J. Y. Y. (2013). *Powder Technol.* **236**, 114–121.

Krahn, F. U. & Mielck, J. B. (1989). *Int. J. Pharm.* **53**, 25–34.

Lang, M., Kampf, J. W. & Matzger, A. J. (2002). *J. Pharm. Sci.* **91**, 1186–1190.

Lipert, M. P. & Rodríguez-Hornedo, N. (2015). *Mol. Pharm.* **12**, 3535–3546.

Lohani, S., Zhang, Y., Chyall, L. J., Mougin-Andres, P., Muller, F. X. & Grant, D. J. W. (2005). *Acta Crystallogr. Sect. E Struct. Reports Online*. **61**, o1310–o1312.

Lowes, M. M. J., Caira, M. R., Lotter, A. P. & Van der Watt, J. (1987). *J. Pharm. Sci.* **76**, 744–752.

Nehm, S. J., Rodríguez-Spong, B. & Rodríguez-Hornedo, N. (2006). *Cryst. Growth Des.* **6**, 592–600.

O'Brien, L. E., Timmins, P., Williams, A. C. & York, P. (2004). *J. Pharm. Biomed. Anal.* **36**, 335–340.

Reboul, J. P., Cristau, B., Soyfer, J. C. & Astier, J. P. (1981). *Acta Crystallogr. Sect. B Struct. Crystallogr. Cryst. Chem.* **B37**, 1844–1848.

Rustichelli, C., Gamberini, G., Ferioli, V., Gamberini, M. C., Ficarra, R. & Tommasini, S. (2000). *J. Pharm. Biomed. Anal.* **23**, 41–54.

Schneider Rauber, G., Bond, A. D., Ho, R., Nere, N. K., Bordawekar, S., Sheikh, A. Y. & Jones, W. (2020*a*). Carbamazepine 1,4-dioxane solvate 2:1 structure report (identifier 1994551). The Cambridge Crystallographic Data Centre.

Schneider Rauber, G., Bond, A. D., Ho, R., Nere, N. K., Bordawekar, S., Sheikh, A. Y. & Jones, W. (2020*b*). Carbamazepine 1,4-dioxane solvate 2:1 structure report (identifier 1994552). The Cambridge Crystallographic Data Centre.

Schneider Rauber, G., Bond, A. D., Ho, R., Nere, N. K., Bordawekar, S., Sheikh, A. Y. & Jones, W. (2020*c*). Carbamazepine benzoquinone cocrystal 1:1 structure report (identifier 1994550). The Cambridge Crystallographic Data Centre.

Sousa, E. T., Da Silva, M. M., De Andrade, S. J., Cardoso, M. P., Silva, L. A. & De Andrade, J. B. (2012). *Thermochim. Acta*. **529**, 1–5.

Umeda, T., Ohnishi, N., Yokoyama, T., Kuroda, K., Kuroda, T., Tatsumi, E. & Matsuda, Y. (1984). *Yakugaku Zasshi*. **104**, 786–792.
